# Applicability of 1,6-Diphenylquinolin-2-one Derivatives as Fluorescent Sensors for Monitoring the Progress of Photopolymerisation Processes and as Photosensitisers for Bimolecular Photoinitiating Systems

**DOI:** 10.3390/polym11111756

**Published:** 2019-10-25

**Authors:** Monika Topa, Filip Petko, Mariusz Galek, Kamil Machowski, Maciej Pilch, Patryk Szymaszek, Joanna Ortyl

**Affiliations:** 1 Faculty of Chemical Engineering and Technology, Cracow University of Technology, Warszawska 24, 30-155 Cracow, Poland; 2 Photo HiTech Ltd., Bobrzyńskiego 14, 30-348 Cracow, Poland

**Keywords:** fluorescent molecular sensors, luminescence spectroscopy, photopolymerisation, cationic photopolymerisation, photosensitizer, photoinduced electron transfer, free-radical photopolymerisation, thiol-ene photopolymerisation

## Abstract

The applicability of new 1,6-diphenylquinolin-2-oneas derivatives as fluorescent molecular sensors for monitoring the progress of photopolymerisation processes by Fluorescence Probe Technique (FPT) has been tested. The progress of cationic, free-radical and thiol-ene photopolymerisation for commercially available monomers: triethylene glycol divinyl ether (TEGDVE), trimethylolpropane triacrylate (TMPTA) and trimethylpropane tris(3-mercaptopropropionate) (MERCAPTO) was monitored. It was found that new derivatives of 1,6-diphenylquinolin-2-one shifted their fluorescence spectra towards shorter wavelengths with the progress of polymerisation, which enabled monitoring the progress in terms of fluorescence intensity ratios as the progress indicator. Derivatives of 1,6-diphenylquinolin-2-one show sensitivity to changes in both polarity and viscosity in the surrounding microenvironment during photopolymerisation processes. Therefore, it was shown that they are good candidates to act as fluorescent sensors for monitoring the kinetics of very quick processes, such as photopolymerisation processes. Furthermore, the effect of the nature of substituents attached to the 1,6-diphenylquinolin-2-one ring on the characteristics of emission spectra was identified. Moreover, the sensitivity of fluorescent sensors was compared with commercially available model sensors, such as 7-diethylamino-4-methylcoumarin (Coumarin 1) and trans-2-(2′,5′-dimethoxyphenyl)ethenyl-2,3,4,5,6-pentafluorobenzene (25ST). Moreover, it was also proven that selected derivatives of 1,6-diphenylquinolin-2-one exhibit an accelerating effect on the progress of cationic photopolymerisation of vinyl monomers (TEGDVE). Thus, the new 1,6-diphenylquinolin-2-one derivatives can be successfully used both as molecular fluorescence sensors to monitor the progress of photopolymerisation processes and as diaryliodonium salt photosensitisers to initiate cationic photopolymerisation processes in a UV-A range of 365 nm.

## 1. Introduction

Modern chemical technologies require increasingly quicker and more accurate methods for monitoring the course of processes in real time, both during production (on-line), including, among others, monitoring the degree of polymerisation, and at the stage of preparation for production or quality control of the finished products (off-line) [1]. In industrial practice, quick photopolymerisation reactions are among the most in demand in on-line monitoring, due to the fact that the properties of the final product depend to a large extent on the conditions in which such photopolymerisation processes are carried out. It is, therefore, necessary to have a sufficiently quick and non-destructive measurement method applied, in order to monitor the progress of these processes in real time. 

Traditional methods of monitoring the photopolymerisation processes include discontinuous methods based on periodical measurements of chemical changes occurring in the polymerising composition. This group includes infrared spectroscopy (IR) [2], Photo-Acoustic Spectroscopy (PAS) [3] and Dynamic Mechanical Analysis (DMA) [4,5,6]. These methods are useful for the identification of residues of unreacted monomers, but are cumbersome, time-consuming and not sufficiently accurate. A different group of methods includes continuous analyses, which enable the monitoring of polymerisation in real time (on-line) during the exposure of the system to light [7,8]. Among the most popular and commonly used methods in this group is isothermal differential scanning calorimetry (photo-DSC) [9,10], which measures the thermal effect of the reaction. Isothermal differential scanning calorimetry measurements are simple and enable measurements in a wide range of temperatures [11]. Its greatest limitation, however, is that the calorimeter measures the thermal effect of the reaction, which makes this method unsuitable for identifying the conversion of the individual monomers included in the composition consisting of more than two monomers. Real-time infrared spectroscopy (real-time-FT-IR) is also a common method used to monitor the fading of absorption bands at a given wavenumber corresponding to the bands of a specific function group [12]. This method, because of its short response time, can be used to determine the conversion rates of very quickly polymerising systems [13]. It does, however, have, certain limitations, such as sensitivity to humidity in the room or measurement chamber, or sensitivity to the presence of oxygen. Furthermore, the thickness of the coating for kinetic measurement by real-time FT-IR should yield absorption in the linear range of the Beer’s law for the functional groups of interest, otherwise the accuracy for using this method will be reduced. From a practical point of view, low-viscosity liquid samples, which we want to use for monitoring the polymerisation kinetic using real-time FT IR, provide a problematic issue. For this type of sample, it is very important to maintain a uniform thickness throughout the entire run of the polymerisation process [14]. As such, these continuous methods are not suitable for applications involving production lines, where the test sample is usually moving in relation to the measuring instrument. Therefore, the application of fluorescence spectroscopy in industrial conditions has a number of advantages, vis-à-vis the traditional methods of monitoring the progress of photopolymerisation processes described above.

Fluorescence Probe Technology (FPT), which uses molecular fluorescence sensors reacting to changes in the surrounding microenvironment, is becoming increasingly popular [15]. For applications in the field of polymers, these sensors are used in the study of the kinetics of polymerisation processes [16]. FPT has a number of important advantages, such as a short response time (<10^−8^ s), which is why this method is perfect for studying very quick processes, such as photopolymerisation [17]. Additionally, it is a non-destructive, high-sensitivity method which is useful for monitoring the progress of photopolymerisation on-line and for the off-line testing of monomers and cured coatings to control the quality of substrates and finished products [18]. The FPT method is based on the measurement of changes in the characteristics of the emission spectrum of the probe against change in the characteristics of the environment, added in the amount of 0.01–0.5% to the tested system [19]. During the photopolymerisation process, due to increased system viscosity or reduced polarity, changes may occur in the fluorescence quantum yield of the probe, the maximum emission spectrum intensity, the fluorescence intensity or the fluorescence polarisation. 

Chemical compounds used as fluorescent sensors should, therefore, have the ability to change the characteristics of fluorescence as their environment changes. In this sense, sensors are compounds with photophysical and photochemical properties dependent on the physicochemical properties of the microenvironment [20]. Thus, fluorescent sensors usually react to changes in the microviscosity and/or polarity of the environment, acting as molecular sensors. As the process of monomer polymerisation progresses, a decrease in the polarity of the system is observed, due to the fact that more polar double bonds in the monomer are transformed into less polar single bonds in the polymer [21]. At the same time, the viscosity of the environment in which the fluorescent sensor is located increases dramatically during polymerisation processes. As a consequence, during polymerisation, a change in the position of the fluorescence spectrum of the sensor is observed, which, when added to the measuring environment, provides information on the progress of the monitored polymerisation reaction. A change in the intensity of fluorescence of the sensor’s emission spectrum is also a common phenomenon observed during polymerisation. Thus, monitoring both the changes in the position of the fluorescence spectrum and its intensity may provide information on the progress of polymerisation kinetics. 

To date, several types of fluorescent probes for monitoring polymerisation processes, which differ in their principles of operation, have been developed [22,23]. Depending on the type of monitored processes, however, a specific structure of the fluorescent sensor molecule is required to guarantee high sensor sensitivity. Therefore, the selection of an appropriate compound as a fluorescent sensor depends on the characteristics of the reaction environment in which the potential sensor will be used [24]. In order to use a fluorescent sensor to monitor a specific process, this should be sufficiently stable during photopolymerisation so as not to exhibit photodegradation before the completion of the monitored process [25]. In addition, fluorescent sensors should be soluble in the reaction environment and have a sufficiently high quantum yield of fluorescence emission to allow measurements at relatively low sensor concentrations. Other important parameters of the probes are their sensitivity and production cost. This is why the idea of designing versatile new sensors based on quinolin-2-one derivatives came to be. 

In this paper, we propose obtaining new derivatives of 1,6-diphenylquinolin-2-one. The reaction products obtained were described using spectroscopic methods, followed by the testing of their absorption and fluorescence characteristics. Spectroscopic studies addressed the question of the potential usefulness of the compounds developed for the role of molecular fluorescence probes in monitoring various types of photopolymerisation processes, including cationic, free radical and thiol-ene photopolymerisation with fluorescence spectroscopy. The sensitivity of the new fluorescent sensors was also compared with commercially available model molecular sensors. The studies showed that the selected obtained derivatives of 1,6-diphenylquinolin-2-one have an accelerating effect during the cationic photopolymerisation process and, furthermore, enable the occurrence of photopolymerisation at the wavelength in which a conventional iodonium photoinitiator in the form of diphenyliodonium salt is not absorbed. Therefore, further in this paper, additional research was carried out to determine the usefulness of the new 1,6-diphenylquinolin-2-one derivatives as iodonium salt sensitizers in initiating cationic photopolymerisation processes in the long-wave ultraviolet (UV-A) range at the wavelength of 365 nm.

## 2. Materials and Methods 

### 2.1. Materials 

**Figure 1:**
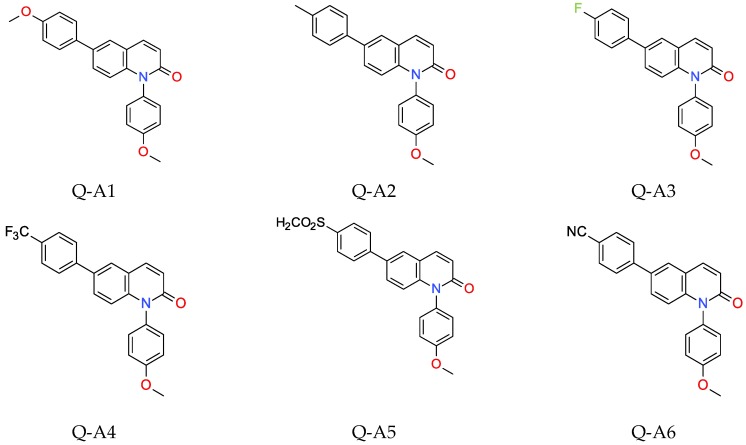
Structures of investigated molecular fluorescent sensors for photopolymerisation processes (series A).

**Figure 2:**
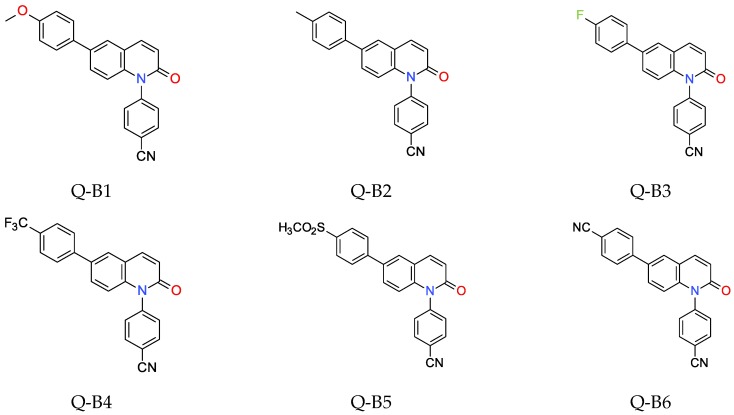
Structures of investigated molecular fluorescent sensors for photopolymerisation processes (series B).

The two series of 1,6-diphenylquinolin-2-one (Q-REF) derivatives were investigated in the role of photosensitizers using various photopolymerisation processes. Series A consisted of: 1,6-bis(4-methoxyphenyl)quinolin-2-one (Q-A1), 1-(4-methoxyphenyl)-6-(4-methylphenyl) quinolin-2-one (Q-A2), 1-(4-methoxyphenyl)-6-(4-fluorophenyl)quinolin-2-one (Q-A3), 1-(4-methoxyphenyl)-6-[4-(trifluoromethyl)phenyl]quinolin-2-one (Q-A4), 1-(4-methoxyphenyl) -6-(4-methylsulfonylphenyl)quinolin-2-one (Q-A5), and 4-[1-(4-methoxyphenyl)-2-oxo-6-quinolyl] benzonitrile (Q-A6). The second series B consisted of the following compounds: 4-[6-(4-methoxyphenyl)-2-oxo-1-quinolyl]benzonitrile (Q-B1), 4-[2-oxo-6-(p-tolyl)-1-quinolyl] benzonitrile (Q-B2), 4-[6-(4-fluorophenyl)-2-oxo-1-quinolyl]benzonitrile (Q-B3), 4-[2-oxo-6-[4-(trifluoromethyl)phenyl]-1-quinolyl]benzonitrile (Q-B4), 4-[6-(4-methylsulfonyl phenyl)-2-oxo-1-quinolyl]benzonitrile (Q-B5), and 4-[1-(4-cyanophenyl)-2-oxo-6-quinolyl]benzonitrile (Q-B6). The studied details of synthesis and physicochemical data of the derivatives of 1,6-diphenylquinolin-2-one are given in the Supporting Information. The structures of the compounds are shown in Figure 1 and Figure 2.

**Figure 3:**
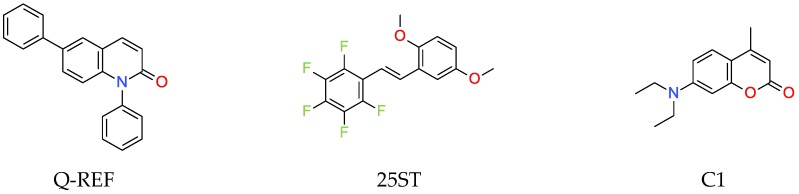
Structures of references of molecular fluorescent sensors.

As a reference, the molecular fluorescent sensors for monitoring the progress of photopolymerisation processes by FPT method were employed respectively: the amine-free fluorescent sensor trans-2-(2′,5′-dimethoxyphenyl)ethenyl-2,3,4, 5,6-pentafluorobenzene (25ST) for the monitoring of cationic photopolymerisation, which was provided by Photo HiTech Ltd. (Cracow, Poland), and 7-diethylamino-4-methylcoumarin (Coumarin 1, from Sigma Aldrich, Darmstadt, Germany) for the monitoring of free-radical and thiol-ene photopolymerisation processes (Figure 3).

Triethylene glycol divinyl ether (TEGDVE, from Sigma Aldrich, Darmstadt, Germany) was applied as a model vinyl ether monomer for the compositions photo-cured by cationic photopolymerisation for real-time FT-IR experiments. 3,4-epoxycyclohexylmethyl-3,4-epoxycyclohexane-carboxylate (S105, Lambson Ltd., Wetherby, UK) was applied as a model monomer for the cationic photopolymerisation processes for photo-DSC measurements. For the role of cationic photoinitiator, diphenyliodonium hexafluorophosphate (HIP, from Alfa Aesar) was used. Trimethylopropane triacrylate (TMPTA, from Sigma Aldrich, Darmstadt, Germany) and 2,2-dimethoxy-2-phenylacetophenone (DMPA, from Sigma Aldrich, Darmstadt, Germany) were employed as a methacrylate monomer and a free-radical photoinitiator for the compositions polymerized by a free-radical mechanism for FPT experiments. Furthermore, trimethylopropane triacrylate (TMPTA, from Sigma Aldrich, Darmstadt, Germany) and trimethylopropane tris(3-mercaptopropionate) (MERCAPTO, from Sigma Aldrich, Darmstadt, Germany) were utilized as a methacrylate and thiol monomers for the compositions polymerized by the thiol-ene mechanism. 2,2-dimethoxy-2-phenylacetophenone (DMPA, from Sigma Aldrich, Darmstadt, Germany) was applied as the photoinitiator in the thiol-ene polymerisation process for FPT experiments.

### 2.2. Spectral Measurements

Absorption spectra of the compounds were recorded in acetonitrile, using the SilverNova spectrometer (StellarNet, Inc., Tampa, FL, USA) in combination with a broadband tungsten-deuterium UV-Vis light source (from StellarNet, Inc., Tampa, FL, USA) and a quartz cuvette with 1.0 cm optical path. Acetonitrile used for the spectroscopic measurements was of analytical grade from Sigma Aldrich (Darmstadt, Germany).

Fluorescence measurements were carried out using the same miniature spectrometer. The spectral characteristics of the sensors were measured in acetonitrile at room temperature (22 °C) using 10-mm-thick quartz cells. As a source of excitation, the UV-LED 320 nm (UVTOP315-BL-TO39, Roithner Laser Technik GmbH, Wien, Austria) light was used. The fiber optic cable, used for transmission of light from the measurement site to the spectrometer, was made of PMMA optical fiber with a 2-mm core (from Fibrochem, Warsaw, Poland).

### 2.3. Steady State Photolysis

During the measurements, cuvettes with appropriate 1,6-diphenylquinolin-2-one derivatives in acetonitrile were irradiated by the UV-LED-365 M365L2 (from Thorlabs Inc., Tampa, FL, USA) emitting light with the wavelength at λ_max_ = 365 nm (~190 mW/cm^2^, current 0.7 A) during 30 min. The source of light was powered by a DC2200 regulated power supply (from Thorlabs Inc., Tampa, FL, USA). The UV-Vis spectra was recorded with the UV/Vis deuterium-halogen light source SL5 (from StellarNet, Inc., Tampa, FL, USA).

### 2.4. Preparation of Samples for Monitoring the Photopolymerisation Processes by FPT

The compositions for FPT measurements were prepared by dissolution of the photoinitiator and each fluorescent sensor in the monomer in such proportions as to obtain the concentration 1.0% by weight of the photoinitiator and 3.69 × 10^−3^ [mol/dm^3^] of the sensor. Before measurement, two drops of the composition were placed in the middle of a microscope slide (75 mm × 25 mm × 1 mm, from Thermo Scientific), equipped with two 0.09-mm-thick spacers located on the slide sides, and the slide was covered with another microscope slide to form a sandwich structure. The thickness of the samples was measured with an electronic microammeter. The slides were kept together using paper clips placed on their side.

### 2.5. Monitoring the Fluorescence Changes During Photopolymerisation by FPT Method

The measurement system was composed of a sample compartment, equipped with a specially designed sensor head where the sample was placed; a Peltier cell-based thermostatic head; a miniature CCD spectrometer (SilverNova from StellarNet, Inc., Tampa, FL, USA), interfaced to a microcomputer for data acquisition; and a UV LED emitting at the wavelength λ_max_ = 320 nm (UVTOP315-BL-TO39, Roithner LaserTechnik GmbH, Austria) incorporated into the sensor head. The structure of the sample compartment with the sensor head and the appearance of the thermostatic head were similar to those reported previously [26]. The UV light from the LED illuminated a spot of about 5 mm within the thin-layer sample. The light from the measurement site was transferred to the spectrometer using a PMMA fiber optic cable with a 2 mm core. The UV LED was supplied with a constant current of 23 mA from an appropriate stabilized constant current source. The sensor head was the same as the one described previously, however, it has been modernized with a thermostat system that guarantees the stability of environmental conditions during the monitoring of the photopolymerisation process. Therefore, all photopolymerisation processes were carried out at an ambient temperature (25 °C) using an ITC4020 thermostat (from Thorlabs Inc., Newton, NJ, USA). A photograph of the measurement system is shown in [27].

### 2.6. Monitoring the Photopolymerisation Processes by Real-Time FT-IR

The cationic photopolymerisation kinetic was investigated using the real-time FT-IR method along with a FT-IR i10 NICOLET^TM^ spectrometer with a horizontal adapter (from Thermo Scientific, Waltham, MA, USA). The compositions for photopolymerisation measurements were prepared by dissolution of each component in the monomer or mixture of monomers. All compositions were prepared in dark glass vials and stored in dark until used. The weight percent of the photoinitiating system is calculated from the monomer content. Because the decrease of absorption of the peak area is directly proportional to the number of polymerized groups, the degree of conversion of the function group was calculated by measuring the peak area at each time of the reaction by using Equation (1):

(1) $$C_{FT-IR}\left[\%\right]=\left(1-\frac{A_{After}}{A_{Before}}\right)*100\%$$

where: *A*_Before_ is an area of the absorbance peak characteristic for the used monomer and type of photopolymerisation before polymerisation process and *A*_After_ is an area of the same absorbance peak, but after polymerisation process. 

The values of the characteristic absorbance peak for studied monomers were given below for each type of photopolymerisation. The evaluation of vinyl group content was continuously followed under air at about 1620 cm^−1^ for 800 s. The light source for the real-time FT-IR method was the 365 nm M365L2 UV-LED diode (power 0.7 A, irradiance *I*_0_ = 190 mW/cm^2^, from Thorlabs Inc., Tampa, FL, USA) powered by a DC2200 regulated power supply (from Thorlabs Inc., Tampa, FL, USA). The UV-LED was started 10 s after the start of spectral registration. The distance between irradiation sources and formulations is 2.1 cm.

### 2.7. Monitoring the Photopolymerisation Processes by Photo-DSC

Photo-DSC studies were conducted with a Photo-DSC 204 F1 Phoenix^®^ from Netzsch -Gerätebau GmbH (Germany). For photopolymerisation processes, an OmniCure^®^ S2000 lamp (production by Excelitas Technologies^®^, Ontario, Canada) with a filter of 365 nm was used in combination with a glass fiber-filled double-core light guide (3 mm fiber diameter). For comparative studies, the light intensity was measured by using an OmniCure^®^ R2000 radiometer (production by Excelitas Technologies^®^, Ontario, Canada) and set to 1 [W/cm^2^] at the tip of the light guide. All measurements were conducted under an inert atmosphere (nitrogen flow of 20 [ml min^−1^]). The measurements were carried out in isothermal mode at 25 °C in the aluminium crucibles was 3 ± 0.5 mg. The heat flow of the reaction was recorded as a function of time. The determinants of the conversion (*C*_photo-DSC_) are the molecular weight, density and the theoretical enthalpy per mol of the functional epoxy group (Δ*H*_teoretical_) of the monomer.

### 2.8. Electrochemical Characteristic Determination of Oxidation and Reduction Potential

The oxidation and reduction potentials of the investigated 2,6-diphenylquinolin-2-one derivatives (E_ox_ vs Ag/AgCl) and the redox potentials (E_red_ vs Ag/AgCl) were measured in acetonitrile by cyclic voltammetry with tetrabutylammonium hexafluorophosphate (0.1 M) (from Sigma Aldrich) as a supporting electrolyte (Electrochemical Analyzer M161 and the Electrode Stand M164, from MTM-ANKO, Cracow, Poland). The working electrode was a platinum disk and the reference was a silver chloride electrode, Ag/AgCl; a scan rate of 0.1 V/s was used; ferrocene was used as a standard and the potentials were determined from half-peak potentials. The Gibbs free energy change Δ*G*et for an electron transfer between the components of the bimolecular photoinitiating system was calculated using the classical Equation (2)

(2) $$G_{et}=F\left[E_{ox}\left(D/D^{•+}\right)-E_{red}\left(A^{•-}/A\right)\right]-E_{00}-\left(Ze^{2}/\varepsilon a\right)$$

where: *E*_ox_ (D/D^•+^) is the oxidation potential of the electron donor; *E*_red_ (A^•−^/A) is the reduction potential of the electron acceptor; *E*_00_ is the excited state energy; and (*Z*e^2^/εa) is the electrostatic interaction energy for the initially formed ion pair. 

Parameter (*Z*e^2^/εa) is generally considered negligible in polar solvents. The excited state energy was determined from the excitation and emission spectra using Quanta Master^TM^ 40 spectrofluorometer (from Photon Technology International (PTI), currently a part of Horiba) at varied excitation wavelengths in the range of 200–800 nm.

## 3. Results

### 3.1. Spectroscopic Properties of 1,6-Diphenylquinolin-2-one Derivatives

**Figure 4:**
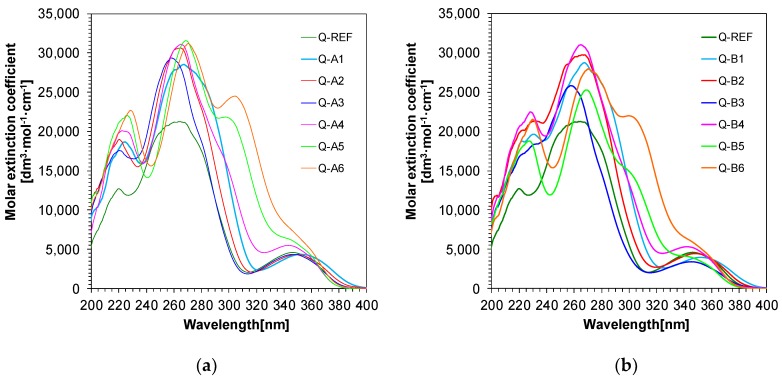
Spectroscopic properties of 1,6-diphenylquinolin-2-one derivatives: (**a**) UV-visible absorption spectra of the 1,6-diphenylquinolin-2-one derivatives in acetonitrile (series A); (**b**) UV-visible absorption spectra of the 1,6-diphenylquinolin-2-one derivatives in acetonitrile (series B).

When specifying the efficiency of new materials as molecular fluorescent sensors, the most essential aspect is to investigate the absorption and fluorescence characteristics of the developed systems. For this purpose, a spectroscopic analysis of 1,6-diphenylquinolin-2-one derivatives was carried out, which revealed that the absorption characteristics of all new compounds is up to 400 nm (Figure 4a,b). The fluorescence spectra of the studied compounds were also measured to examine their spectroscopic properties. All 1,6-diphenylquinolin-2-one derivatives demonstrated a sufficient fluorescence level (Table 1) for their spectral characteristics, which was easily measured at a probe concentration of ca. 0.1% by weight and a sample thickness of ca. 0.1 mm. This is highly important because when the studied compounds are used as molecular fluorescent sensors in FPT, the sensor should present the strongest fluorescence possible (i.e., not less than 1000 [absolute units] for the spectrometer) when excited with a light wavelength of at least 320 nm. The wavelength selection is not accidental, as when the excitation light with shorter wavelengths is used, their intensity is strongly attenuated by the microscope glass slides that are normally used for preparing thin-layered samples in FPT. In this case, the Stokes shift is identified, which is a complex function of electron density distribution in the excited and basic state of the analysed molecular sensors. The Stokes shift value changes depending on the electron-acceptor nature of the substituents attached to the 1,6-diphenylquinolin-2-one structure.

Molecular fluorescent sensors intended for practical applications in FPT should have the greatest Stokes shift possible to prevent the effect of the excitation light spectrum reflected from the studied sample from overlapping with the sensor fluorescence spectrum. Consequently, measures are taken to eliminate or minimise the effect so that it does not disturb the shape of the fluorescent spectrum of the sensor used for the tests. This is particularly important for sensors with low fluorescence intensity in the studied environment. Hence, from the point of view of fluorescence spectrum separation from the excitation light spectrum, all of the obtained 1,6-diphenylquinolin-2-one derivatives reveal correct spectroscopic characteristics. Spectroscopic data of 1,6-diphenylquinolin-2-one derivatives are presented in Table 1, while absorption characteristics are shown in Figure 4. 

**Figure 5:**
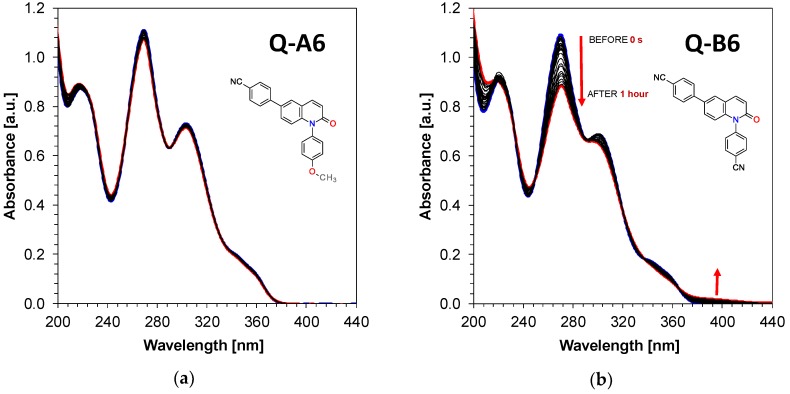
Steady state photolysis of: (**a**) 4-[1-(4-methoxyphenyl)-2-oxo-6-quinolyl]benzonitrile (Q-A6) under irradiation upon UV-LED at 365nm (135 mW/cm^2^); (**b**) 4-[1-(4-cyanophenyl)-2-oxo- 6-quinolyl]benzonitrile (Q-B6) under irradiation upon UV-LED at 365nm (135 mW/cm^2^).

**Figure 6:**
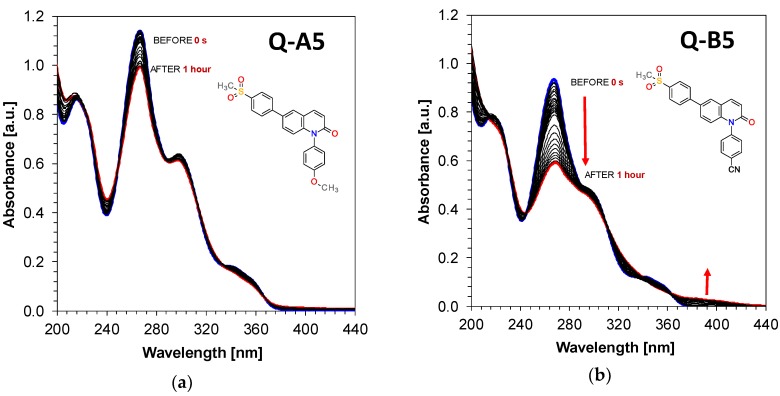
Steady state photolysis of: (**a**) 1-(4-methoxyphenyl)-6-(4-methylsulfonylphenyl) quinolin-2-one (Q-A5) under irradiation upon UV-LED at 365 nm (135 mW/cm^2^); (**b**) 4-[6-(4-methylsulfonylphenyl)-2-oxo-1-quinolyl]benzonitrile (Q-B5) under irradiation upon UV-LED at 365 nm (135 mW/cm^2^).

During spectroscopic investigation of the developed 1,6-diphenylquinolin-2-one derivatives, their photostability was also analyzed under continuous exposure to UV-LED-emitting light with a wavelength of 365 nm and intensity I_0_ = 135 [mW/cm^2^] for 1 hour. The performed analysis revealed that 1,6-diphenylquinolin-2-one derivatives are highly photostable during irradiation with UV-A light, whereby 1,6-diphenylquinolin-2-one derivatives from A series have slightly higher absorption stability characteristics during exposure to light than their equivalent derivative compounds from the B series. The data related to the photostability of the examined derivatives are presented in Figure 5 and Figure 6.

### 3.2. Applicability of 1,6-Diphenylquinolin-2-one Derivatives for On-line Progress Monitoring of Free-radical and thiol-ene Photopolymerisation Processes

Different features of the emitted fluorescence spectrum can be used for on-line monitoring of changes in the molecular fluorescent sensor environment, including fluorescence intensity changes at maximum emission intensity [28], changes in the fluorescence spectrum maximum position [29,30] etc. Nonetheless, from an application point of view, it is best to use the measurement of fluorescence intensity ratio (R), measured for two different wavelengths located on both sides of the molecular sensor fluorescence spectrum maximum for continuous quantitative monitoring of very fast processes, such as photopolymerisation [31]. An unquestionable advantage of using this parameter is the ability to measure the ratio intensity accurately, as was confirmed in subsequent studies [32]. This can be attributed to the fact that when the lengths of the waves arranged on both sides of the fluorescence spectrum maximum are appropriately selected, the fluorescence intensity ratio (R) is a linear function of the degree of conversion of monomer function groups [33,34,35]. 

**Figure 7:**
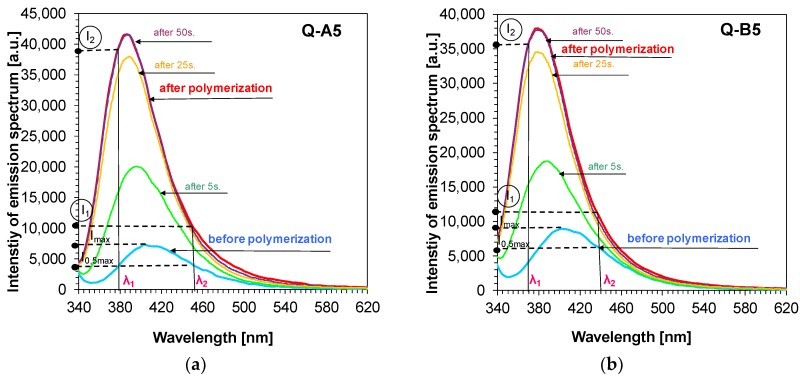
(**a**) Changes of fluorescence spectra of example Q-A5 sensor during free-radical photopolymerisation of TMPTA monomer under irradiation of 320 nm, (λ1, λ2 are monitoring wavelengths); (**b**) Changes of fluorescence spectra of example Q-B5 sensor during free-radical photopolymerisation of TMPTA monomer under irradiation of 320 nm, (λ1, λ2 are monitoring wavelengths).

Bearing that in mind, the fluorescence intensity ratio (R) was used for monitoring the free-radical photopolymerisation of the reference composition based on a TMPTA monomer. For 1,6-diphenylquinolin-2-one derivatives, this parameter was defined as the ratio of fluorescence intensity at the shorter wavelength (λ_1_) to the fluorescence intensity at the longer wavelength (λ_2_), which is presented in Figure 7 a and b for Q-A5 and Q-B5 derivatives. 

**Figure 8:**
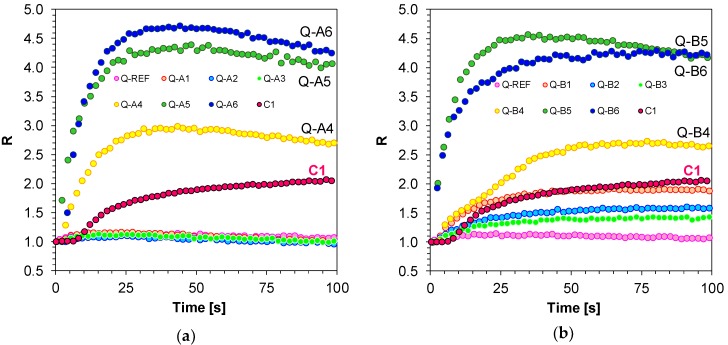
(**a**) Monitoring free-radical photopolymerisation of TMPTA monomer under 320 nm by FPT, using the 1,6-diphenylquinolin-2-one derivatives (series A: Q-A1–Q-A6) as the fluorescent sensors and Coumarin 1 (C1) as a reference probe; (**b**) Monitoring free-radical photopolymerisation of TMPTA monomer under 320 nm by FPT, using the 1,6-diphenylquinolin-2-one derivatives (Series B: Q-B1–Q-B6) as the fluorescent sensors and Coumarin 1 (C1) as a reference probe.

The study also included an analysis of the comparison between the sensitivity of the newly developed molecular fluorescent sensors and the sensitivity of well-known commercial sensors, such as Coumarin 1. To that end, the λ_1_ and λ_2_ wavelengths were selected individually for each 1,6-diphenylquinolin-2-one derivative. The length of the λ_1_ wave was selected so that it was located in the middle of the linear section of the fluorescence spectrum on the short wavelength side prior to polymerisation. The same method was used for selecting the length of the wave at longer wavelengths (λ_2_)_,_ whereby, in this case, the intensity at λ_2_ before polymerisation was the same as intensity at λ_1_. The method of determining the parameter as a ratio of fluorescence intensity is presented in Figure 7a,b. That is why, as a result of the correct determination of R parameters, all values for each analysed 1,6-diphenylquinolin-2-one derivative started with 1.0 and increased if the fluorescence spectrum shifted towards a shorter wavelength or remained stable if the spectrum did not shift (Figure 8a,b). 

**Figure 9:**
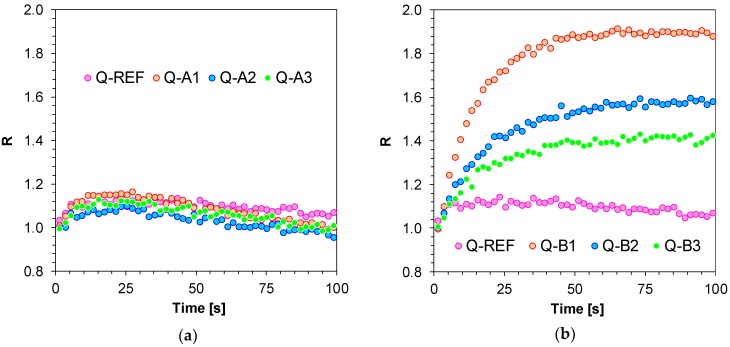
(**a**) Monitoring free-radical photopolymerisation of TMPTA monomer under 320 nm by FPT, using the low sensitivity fluorescent sensors based on 1,6-diphenylquinolin-2-one derivatives (Series A); (**b**) Monitoring free-radical photopolymerisation of TMPTA monomer under 320 nm by FPT, using the low sensitivity fluorescent sensors based on 1,6-diphenylquinolin-2-one derivatives (Series B).

The applicability of 1,6-diphenylquinolin-2-one derivatives as molecular sensors during free-radical photopolymerisation revealed that the studied compounds, depending on their structure and the nature of the electron-acceptor substituents embedded in the 1,6-diphenylquinolin-2-one chromophore, demonstrate different sensitivity, which is confirmed by the different ranges of R variation. Sensors with strong electron-acceptor-like groups, in the form of cyanide groups (-CN) (Q-A6 and Q-B6) or methane sulphate groups (-SO_2_CH_3_) (Q-A5 and Q-B5) and trifluoromethylene groups (-CF_3_) (Q-A4 and Q-B4) in their structure and attached to a phenyl ring embedded in the 1,6-diphenylquinolin-2-one chromophore at position 6 (Figure 8a,b) proved to be most sensitive. The sensitivity of the sensors is nearly 2.5 times higher than the sensitivity of the Coumarin 1 (C1) reference probe, which means that they are more suitable for monitoring the free-radical polymerisation processes than Coumarin 1. Sensors with electron-donor substituents attached to a phenyl ring in the 1,6-diphenylquinolin-2-one chromophore at position 6 (i.e., 1,6-bis(4-methoxyphenyl)quinolin-2-one (Q-A1), 1-(4-cyanophenyl)-6-(4-methoxyphenyl) quinolin-2-one (Q-B1), 1-(4-methoxyphenyl)-6-(4-methylphenyl)quinolin-2-one (Q-A2), 1-(4-cyanophenyl)-6-(4-methylphenyl)quinolin-2-one (Q-B2), 1-(4-methoxyphenyl)-6- (4-fluorophenyl)quinolin-2-one (Q-A3), and 1-(4-cyanophenyl)-6-(4-fluorophenyl)quinolin-2-one (Q-B3) sensors) revealed the lowest change in R value during acrylate monomer (TMPTA) polymerisation (Figure 9a,b). It can then be concluded that a strong delocalisation of charge when the probe is excited, amplified by the push-pull effect of electron-donor and electron-acceptor substituents arranged on the opposite sides of a coupled system of bonds, is necessary for the correct functioning of fluorescent sensors based on 1-(4-methoxyphenyl)-6-phenyl-quinolin-2-one or 4-(2-oxo-6-phenyl-1-quinolyl)benzonitrile chromophores. If in a 1,6-diphenylquinolin-2-one chromophore the substituents are of an electron-donor nature, like the 1,6-bis(4-methoxyphenyl)quinolin-2-one (Q-A1), 1-(4-cyanophenyl)-6-(4-methoxyphenyl) quinolin-2-one (Q-B1), 1-(4-methoxyphenyl)-6-(4-methylphenyl)quinolin-2-one (Q-A2), 1-(4-cyanophenyl)-6-(4-methylphenyl)quinolin-2-one (Q-B2), 1-(4-methoxyphenyl)- 6-(4-fluorophenyl)quinolin-2-one (Q-A3), and 1-(4-cyanophenyl)-6-(4-fluorophenyl)quinolin-2-one (Q-B3) sensors, delocalisation does not take place and the probe becomes insensitive to changes in its environmental polarity.

It was observed that the fluorescence intensity of all studied sensors increased significantly during the free-radical photopolymerisation of the monomer (Figures S14–S27 presented in the Supporting Information), which is testimony to the fact that all studied 1,6-diphenylquinolin-2-one derivatives are sensitive to environmental microviscosity changes. An increase in the fluorescence intensity of the studied sensors as the system viscosity rises, results from the higher quantum efficiency of fluorescence caused by a reduction in the competitive process of non-radiated dissipation of excited state energy. Thus, in an environment with high viscosity, an excited sensor molecule does not have as much freedom to dissipate excitation energy between the vibration and rotary states of the molecule as in the case of low-viscous solutions. 

In order to compare the sensitivity of the developed sensor quantitatively depending on the substituent type and position in the quinolin-2-one structure, the relative sensitivity parameter *S_rel_* was introduced.

(3) $$S_{rel}=\frac{\left[\frac{\left(R_{max}-R_{0}\right)}{R_{0}}\right]}{\left[\frac{\left(R_{max-ref}-R_{0-ref}\right)}{R_{0-ref}}\right]}$$

where: *R*_0_—*R* value for the analysed sensor in the composition before photopolymerisation; *R*_max_—*R* value for the analysed sensor in the composition after photopolymerisation; *R*_0 –ref_—*R* value for the reference sensor in the composition before photopolymerisation; and *R*_max-ref_—*R* for the reference sensor in a photo-cured composition.

The calculated sensitivity values differ for individual probes (Table 2), which clearly indicates that the sensitivity of the compounds depends on the nature of the substituent attached to the quinolinon-2-one ring. Nonetheless, much higher sensitivity as compared to the Coumarin 1 reference was observed for the compounds with electron-acceptor substituents located at position 4 of the phenyl ring attached to the quinolin-2-one chromophore for A and B series compounds. Thus, the type of substituent greatly affects their sensitivity when their physical and chemical characteristics change during free-radical photopolymerisation. 

**Figure 10:**
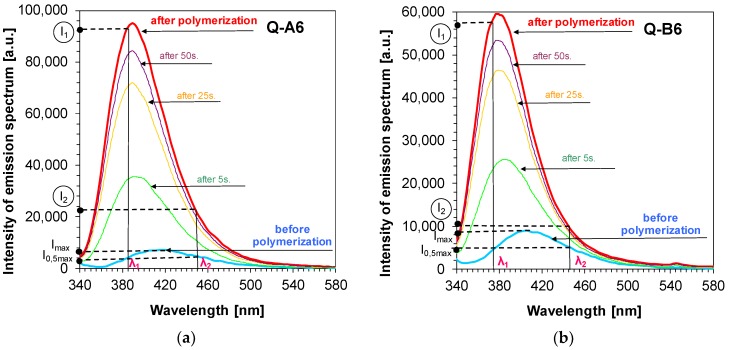
(**a**) Changes of fluorescence spectra of example Q-A6 sensor during thiol-ene photopolymerisation of TMPTA/MERCAPTO (0.5%/0.5% w/w) monomers under irradiation of 320 nm, (λ_1_, λ_2_ are monitoring wavelengths); (**b**) Changes of fluorescence spectra of example Q-B6 sensor during thiol-ene photopolymerisation of TMPTA/MERCAPTO (0.5%/0.5% w/w) monomers under irradiation of 320 nm, (λ_1_, λ_2_ are monitoring wavelengths).

Equivalent measurements by means of FPT and using the developed 1,6-diphenylquinolin-2-one derivatives were carried out for the thiol-ene photopolymerisation process of acrylate (TMPTA) and thiol (MERCAPTO) monomers (0.5/0.5% by weight). The R parameter was used for on-line monitoring of thiol-ene photopolymerisation progress, just like in the case of free-radical photopolymerisation (Figure 10).

**Figure 11:**
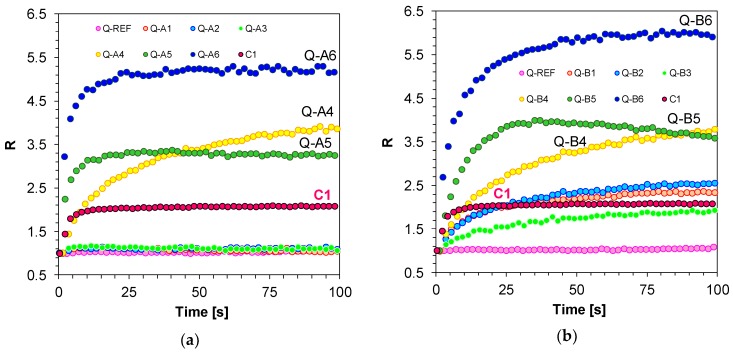
(**a**) Monitoring thiol-ene photopolymerisation of TMPTA/MERCAPTO (0.5%/0.5% w/w) monomers under 320 nm by FPT, using molecular fluorescent sensors based on 1,6-diphenylquinolin-2-one derivatives (0.1 wt.%) (Series A); (**b**) Monitoring thiol-ene photopolymerisation of TMPTA/MERCAPTO (0.5%/0.5% w/w) monomers under 320 nm by FPT, using molecular fluorescent sensors based on 1,6-diphenylquinolin-2-one derivatives (0.1 wt.%) (Series B).

Similar to free-radical photopolymerisation, a much higher sensitivity, as compared to the Coumarin 1 reference sensor (C1), was observed for compounds with electron-acceptor substituents located at position 4 of the phenyl ring found at position 6 of the quinolin-2-one chromophore (Figure 11). 

### 3.3. Applicability of the 1,6-Diphenylquinolin-2-one for On-line Progress Monitoring of Cationic Photopolymerisation of Monomers

**Figure 12:**
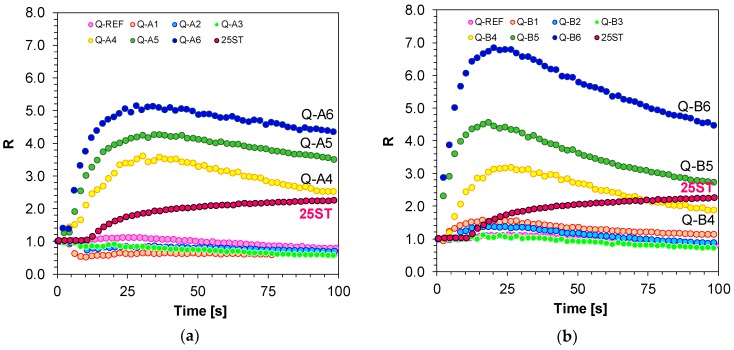
(**a**) Monitoring cationic photopolymerisation of TEGDVE monomer under 320 nm by FPT, using the 1,6-diphenylquinolin-2-one derivatives (series A: Q-A1–Q-A6) as the fluorescent sensors and 25ST as a reference sensor; (**b**) Monitoring cationic photopolymerisation of TEGDVE monomer under 320 nm by FPT, using the 1,6-diphenylquinolin-2-one derivatives (series B: Q-B1–Q-B6) as the fluorescent sensors and 25ST as a reference sensor.

An attempt was made to monitor cationic photopolymerisation of TEGDVE vinyl monomers with diphenyliodonium hexafluorophosphate (HIP) used as a photoinitiator. An appropriate 1,6-diphenylquinolin-2-one derivative in the amount of 3.0 × 10^−3^ [mol/dm^3^] was added to the composition as a fluorescent sensor. Based on the shape of the kinetic curves obtained with the FPT method (Figure 12a,b), it can be concluded that not all studied sensors enable tracing of the changes in the system by means of fluorescence intensity ratios. Similar to free-radical and thiol-ene photopolymerisation monitoring, the highest sensitivity was demonstrated by derivatives with electron-acceptor substituents attached to the phenyl ring located at position 6 of the quinolinon-2-one chromophore. The derivatives responded to the changes right from the beginning of cationic photopolymerisation, which was demonstrated by an increase in parameter R and the high slope of the R to time ratio. As reactive vinyl groups in the system disappeared with time, the photopolymerisation rate decreased gradually to vanish completely, which resulted in the kinetic curves reaching a plateau. The studied fluorescent sensors varied in their sensitivity, as suggested by a different range in R variation. The sensitivity of new quinolin-2-one derivatives was compared with the commercially available reference probe 25ST, which is used as a sensor to monitor cationic photopolymerisation progress by means of the R parameter [36]. 

### 3.4. Comparative Analysis of the Sensitivity of Investigated Molecular Fluorescent Sensors Depending on Their Structure

**Figure 13:**
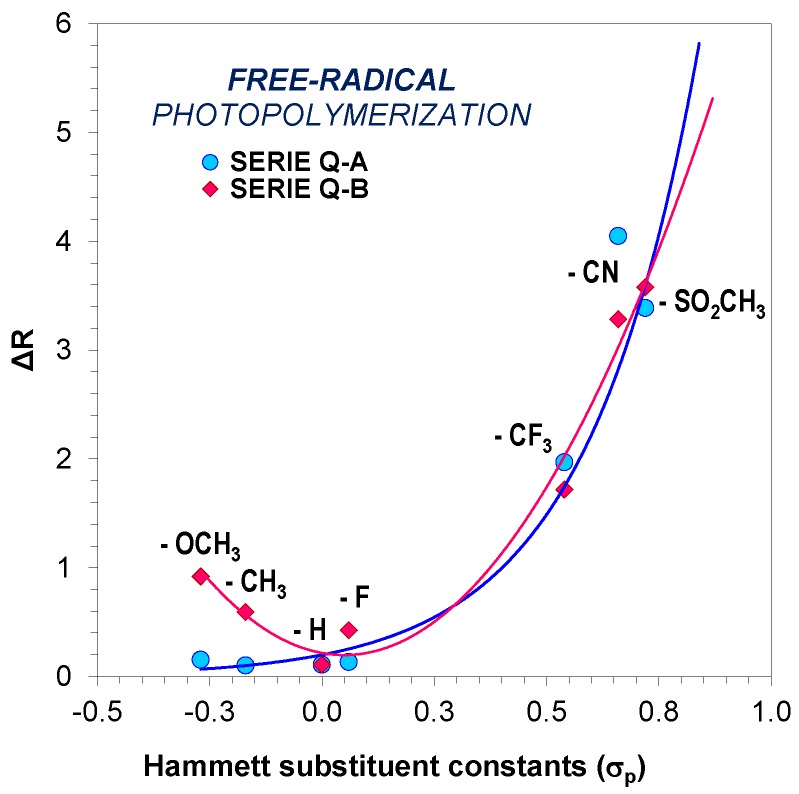
Effect of substituents on the sensitivity (ΔR) of 1,6-diphenylquinolin-2-one derivative (series A and B) probes in monitoring of free-radical photopolymerisation of the TMPTA monomer.

Upon obtaining results for three types of free-radical, thiol-ene and cationic photopolymerisation processes, the substituents were analysed in regard to the sensitivity of the studied 1,6-diphenylquinolin-2-one derivatives and the quantitative changes in their environment (Table 2). In order to explain the relation between the structure of these compounds and the ability of the fluorescent molecular probes to monitor free-radical polymerisation, the comparison was made of the relationship between the ratio span between the uncured and cured state of the monomer (Δ*R*) and the Hammett substituent constants values (σ_p_) (Figure 13). For this purpose, the Hammett equations were used, which are normally employed for assessing the impact of substituents on the kinetics or thermodynamics of chemical reactions [36]. 

The relationships between the sensitivity and the Hammett substituent constant (σ_p_) presented for free-radical photopolymerisation are not linear relationships. It was observed that the sensitivity of the fluorescent probes increases with an increase of the electron-withdrawing character of the substituent. This relationship indicates that sensitivity of the 1,6-diphenylquinolin-2-one derivatives to changes occurring in a polymerizing medium strongly depends on the type of substituent on the quinolin-2-one moiety. Nonetheless, it is clear that strongly electron-withdrawing –CN and –SO_2_(CH_3_)_2_ substituents exhibit a stronger influence on the fluorescence probe sensitivity than weak electron-withdrawing and electron-donating substituents.

### 3.5. Performance of 1,6-Diphenylquinolin-2-one Derivatives as Photosensitisers in Bimolecular Photoinitiating Systems for Cationic Photopolymerisation Processes of Different Types of Monomers

During studies on the possibility of using 1,6-diphenylquinolin-2-one derivatives as molecular sensors for cationic photopolymerisation monitoring, it was discovered that some of the developed compounds effectively accelerate the monitored photopolymerisation. For this reason, measurements were carried out concerning vinyl monomer cationic photopolymerisation monitoring at the UV-A range wavelength with an emission maximum of 365 nm. The commonly available iodonium salt in the form of diphenyliodonium hexafluorophosphate (HIP) was used as a photoinitiator during cationic polymerisation monitoring. The absorption characteristics of the photoinitiator fall within the UV-C and UV-B range with a maximum at λ_max_ = 242 nm [29,37]. This is why the initiator sensitivity is within the range of short UV light wavelengths, which is a serious process problem since photopolymerisation cannot take place at longer UV-A or visible light wavelengths when this kind of compound is used. This problem can be solved by using relevant photosensitisers, whose absorption characteristics are within the range of longer wavelengths than in the case of the iodonium photoinitiator. In this approach, cationic polymerisation at a longer wavelength above 350 nm, which is not absorbed by iodonium salt, can be initiated through photosensitisation by way of electron transfer. With an appropriately selected photosensitiser, a photoinduced electron transfer (PET) is possible. The PET is a non-standard endothermic energy transfer process in which an absorbed light quantum initiates electron transfer from the donor molecule (i.e., the sensibiliser) to the acceptor molecule, i.e., iodonium salt in this case. 

**Figure 14:**
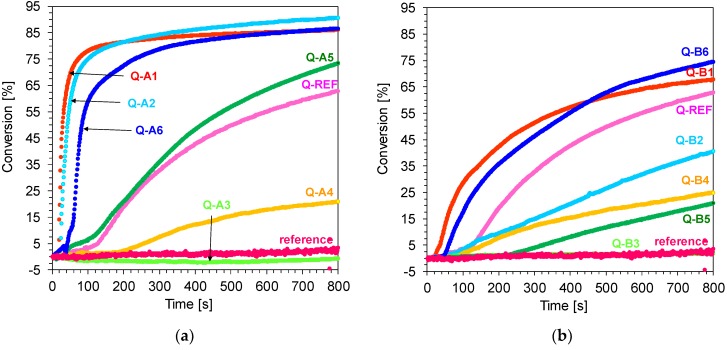
(**a**) Polymerisation profiles of TEGDVE (vinyl function conversion vs. irradiation time) upon exposure to the LED at 365 nm in the presence of different photoinitiating systems based on diphenyliodonium hexafluorophosphate (HIP, 1 wt.%) and 1,6-diphenylquinolin-2-one derivatives (0.1 wt.%) (series A). The irradiation starts at t = 10 s.; (**b**) Polymerisation profiles of TEGDVE (vinyl function conversion vs. irradiation time) upon exposure to the LED at 365 nm in the presence of different photoinitiating systems based on diphenyliodonium hexafluorophosphate (HIP, 1 wt.%) and 1,6-diphenylquinolin-2-one derivatives (0.1 wt.%) (series B). The irradiation starts at t = 10 s.

The real-time FT-IR method was used for cationic photopolymerisation monitoring in order to identify monomer final conversions. Band fading for vinyl monomers (TEGDVE) was observed at a wave number of about 1620 cm^−1^ during the process of cationic photopolymerisation. The wave number corresponds to the disappearance of double bonds in a monomer. The process was initiated by using UV-LED as a light source, which emitted electromagnetic radiation at a maximum wavelength of λ_max_ = 365 nm. Based on the recorded curves, it was demonstrated that the majority of the studied 1,6-diphenylquinolin-2-one derivatives effectively initiate vinyl monomer photopolymerisation by reaching conversion levels from 60% up to 80% (Figure 14). 

**Figure 15:**
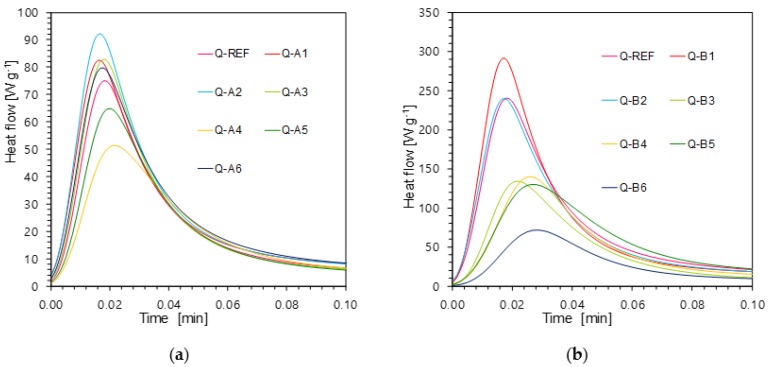
(**a**) Photopolymerisation heat flow curves of epoxy monomer S105 using different photoinitiating systems based on diphenyliodonium hexafluorophosphate (HIP, 1%) and 1,6-diphenylquinolin-2-one derivatives (0.1%) (series A); (**b**) Photopolymerisation heat flow curves of epoxy monomer S105 using different photoinitiating systems based on diphenyliodonium hexafluorophosphate (HIP, 1 wt.%) and 1,6-diphenylquinolin-2-one derivatives (0.1 wt.%) (series B).

The photoinduced ring-opening cationic photopolymerisation of a cycloaliphatic epoxide monomer (S105) in the presence of two-component photoinitiating systems based on 1,6-diphenylquinolin-2-one derivatives and diphenyliodonium hexafluorophosphate (HIP) in combinations of 0.1%/1% w/w was also investigated upon UV-LED exposure at 365 nm using photo-DSC equipment. Most of the investigated quinolin-2-one derivatives have the ability to initiate the process of photoinduced ring-opening polymerisation of the S105 monomer (Figure 15). 

**Figure 16:**
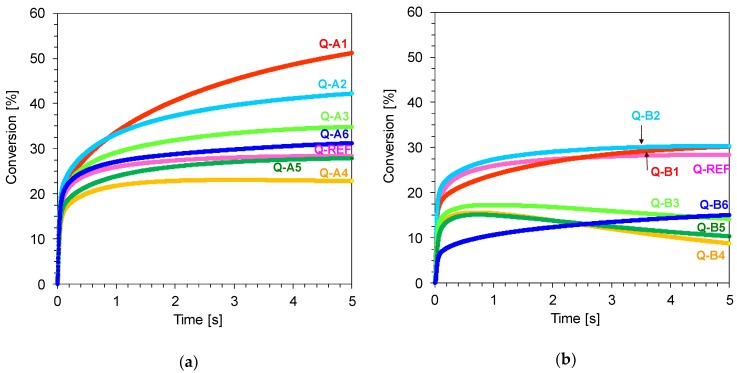
(**a**) Comparative photo-DSC conversion curves for cationic photopolymerisation of epoxy monomer S105 using different photoinitiating systems based on diphenyliodonium hexafluorophosphate (HIP, 1 wt.%) and 1,6-diphenylquinolin-2-one derivatives (0.1 wt.%) (series A); (**b**) Comparative photo-DSC conversion curves for cationic photopolymerisation of epoxy monomer S105 using different photoinitiating systems based on diphenyliodonium hexafluorophosphate (HIP, 1 wt.%) and 1,6-diphenylquinolin-2-one derivatives (0.1 wt.%) (series B).

Based on the recorded kinetic profiles, it was demonstrated that the highest conversion grades for series A and B were obtained for the initiating systems which included 1,6-diphenylquinolin-2-one derivatives with electro-donor substituents in the form of methoxy and methyl groups connected at position 4 of the phenyl ring located at position 6 of the quinolin-2-one chromophore. Nevertheless, it should be highlighted that bimolecular systems based on A series compounds, i.e., 1-(4-methoxyphenyl)-6-phenyl-quinolin-2-one derivatives (Q-A1), show higher conversion values for the cycloaliphatic epoxy monomer (S105) (Figure 16).

This means that the best bimolecular photoinitiating systems were the ones which, besides iodonium salt, contained 1,6-diphenylquinolin-2-one that has electron-donor substituents at positions 1 and 6 of the quinolin-2-one chromophore (e.g., Q-A1, Q-B1, Q-A2, Q-B2, Q-A3 ad Q-B3).

Cyclic voltammograms (CV) of the sensors (Figures S56–S68 included in the Supporting Information) were measured to confirm the possibility of electron transfer between a fluorescent molecular sensor and iodonium salt. The electrochemically identified oxidation potentials of the studied compound half-waves and the calculated values of the free energy change (Δ*G*_et_) are given in Table 3.

The data in Table 3 indicate that with an increase of electron-withdrawing substituents attached into 1,6-diphenylquinolin-2-one chromophore, there is an increase in the oxidation potential of sensitizers. Moreover, with the increase of electron-withdrawing substituents, the energy of singlet and triplet excited states of the quinolin-2-one goes down, because both the absorption and the fluorescence spectra shift towards longer wavelengths. Nevertheless, the above results indicate that the compounds can act as photosensitises and the electron transfer process from excited singlet or triplet sensibiliser states to iodonium salt (HIP) is possible due to the fact that the free energy change (Δ*G*_et_) is negative. However, the free enthalpy change of the electron transfer (Δ*G*_et_) goes down (to obtain more negative value) with an increase of the electron-donating character of the substituent, and consequently, the rate of the electron transfer goes up, increasing the magnitude of electron transfer efficiency. It can be observed that the electron transfer from all excited 1,6-diphenylquinolin-2-one derivatives to iodonium salt is acceptable from a thermodynamic point of view. It was demonstrated that depending on the oxidation potential of 1,6-diphenylquinolin-2-one derivatives, the value of the calculated free energy change (Δ*G*_et_) varies depending on the structure of the studied sensor (Table 3).

## 4. Conclusions

Based on this study, it was demonstrated that depending on the type of substituent in a 1,6-diphenylquinolin-2-one molecule, the compounds reveal different sensitivity to the changes in their microenvironment. Among the studied 1,6-diphenylquinolin-2-one derivatives, the best characteristics pertaining to probe sensitivity and monomer conversion were exhibited by the derivatives which have electron-acceptor substituents at position 4 of the phenyl ring attached to the quinolin-2-one chromophore. A hypsochromic shift towards shorter waves was observed for all compounds in the group during the analysed photopolymerisation processes. Such behaviour enables photopolymerisation monitoring by means of the fluorescence intensity ratio (R). The use of R as a polymerisation progress indicator eliminates the impact of such parameters as excitation light intensity changes, the measured sample thickness, the distance of the measurement head to the sample, and probe concentration in the composition, on the R measurement results. This is why the sensors 1,6-bis(4-methoxyphenyl)quinolin-2-one (Q-A1), 1-(4-cyanophenyl)-6-(4-methoxyphenyl) quinolin-2-one (Q-B1), 1-(4-methoxyphenyl)-6-(4-methylphenyl)quinolin-2-one (Q-A2), 1-(4-cyanophenyl)-6-(4-methylphenyl)quinolin-2-one (Q-B2), 1-(4-methoxyphenyl)-6- (4-fluorophenyl)quinolin-2-one (Q-A3), and 1-(4-cyanophenyl)-6-(4-fluorophenyl)quinolin-2-one (Q-B3) are suitable for off-line and on-line applications. The compounds are completely new and constitute universal sensors for monitoring the progress of different photopolymerisation types. Owing to easy synthesis and the lower cost of raw materials, they are an interesting alternative to the currently known fluorescent sensors, namely Coumarin 1.

The study revealed that some derivatives of 1,6-diphenylquinolin-2-one accelerate cationic photopolymerisation. It was confirmed that photoinduced electron transfer (PET) is the mechanism responsible for this phenomenon. It was demonstrated that depending on the oxidation potential of 1,6-diphenylquinolin-2-one derivatives, the value of the calculated free energy change (ΔG_et_) varies depending on the structure of the studied sensor. The most effective bimolecular initiating systems are those which use A series derivatives of 1-(4-methoxyphenyl)-6-phenyl-quinolin-2-one with electron-donor substituents in the phenyl ring located at position 6 of the quinolin-2-one chromophore.

## Figures and Tables

**Table 1:** Spectral characteristics of the 1,6-diphenylquinolin-2-one derivatives studied.

Serie	Acronym	R_1_	R_2_	Absorption		Emission @λ_ex_ =320 nm		Δν Stoke’s Shift [cm^−1^]
				λ_max-ab_^a^ [nm]	ɛ_max_^b^ [dm^3^·mol^-1^·cm^-1^]	λ_max-fluo_^c^ [nm]	I_max_^d^ [rel.u.]	
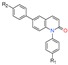	Q-REF	–H	–H	346	4600	419	2800	5035
	Q-A1	–OCH_3_	–OCH_3_	352	4370	431	2400	5207
	Q-A2	–OCH_3_	–CH_3_	349	4350	424	1660	5068
	Q-A3	–OCH_3_	–F	347	4360	408	1310	4309
	Q-A4	–OCH_3_	–CF_3_	343	5500	404	1200	4402
	Q-A5	–OCH_3_	–SO_2_CH_3_	350	5700	422	1860	4875
	Q-A6	–OCH_3_	–CN	350	6740	417	2040	4591
	Q-B1	–CN	–OCH_3_	352	3970	440	3350	5682
	Q-B2	–CN	–CH_3_	348	4460	420	2020	4926
	Q-B3	–CN	–F	345	3410	423	1830	5345
	Q-B4	–CN	–CF_3_	342	5330	414	460	5085
	Q-B5	–CN	H_3_CO_2_S–	340	4140	423	1830	5771
	Q-B6	–CN	–CN	340	6510	415	6690	5315

^a^ position of absorption maximum for the long-wavelength band [nm]. ^b^ molar extinction coefficient measured at λ_max-ab_ [dm^3^ mol^−1^ cm^−1^]. ^c^ position of maximum fluorescence intensity [nm]. ^d^ intensity of the fluorescence at λ_max-fluo_.

**Table 2:** Spectroscopic data of 1,6-diphenylquinolin-2-one derivatives during the photopolymerisation processes.

**Sensor**	**λ _max-BEFORE_ [nm]**	**Intensity @λ_max-BEFORE_ [a.u.]**	**λ_max-AFTER POL_ [nm]**	**Intensity @λ_max-AFTER_ [a.u.]**	**|ΔI_max_| [a.u.]**	**ΔI_max_^a^ [%]**	**Δλ_max_ [nm]**	**Relative Sensitivity ^b^**
**Free-radical photopolymerisation process of TMPTA under 320 nm**								
Q-REF	418	3800	418	2769	1031	27	0	0.10
Q-A1	435	6122	439	4631	1492	24	-4	0.14
Q-A2	423	3605	428	2931	675	19	-5	0.10
Q-A3	422	3384	424	2769	615	18	-2	0.13
Q-A4	412	5117	376	10,978	5861	115	36	1.83
Q-A5	403	7315	387	41,684	34,369	470	17	3.17
Q-A6	399	45,037	386	268,699	223,663	497	13	5.66
Q-B1	442	6543	431	7331	789	12	11	0.86
Q-B2	426	4434	414	4868	434	10	12	0.55
Q-B3	423	3330	416	3203	127	4	7	0.40
Q-B4	401	10,385	374	20,060	9674	93	27	1.69
Q-B5	403	8962	384	37,048	28,086	313	19	3.38
Q-B6	400	10,747	386	63,463	52,716	490	14	4.29
C1-ref.	399	45,037	386	268,699	223,663	497	13	1.00
**Sensor**	**λ_max-BEFORE_ [nm]**	**Intensity λ_max-BEFORE_ [a.u.]**	**λ_max-AFTER_ [nm]**	**Intensity @λ_max-AFTER_ [a.u.]**	**|ΔI_max_| [a.u.]**	**ΔI_max_^a^ [%]**	**Δλ_max_ [nm]**	**Relative Sensitivity ^b^**
**Thiol-ene photopolymerisation process of TMPTA/MERCAPTO (50/50 %w/w) under 320 nm**								
Q-REF	419	1858	421	1522	336	18	−2	0.07
Q-A1	437	1741	431	1249	492	28	7	0.13
Q-A2	438	2015	430	1682	333	17	8	0.12
Q-A3	419	1812	421	1543	269	15	−2	0.14
Q-A4	414	3696	386	9116	5420	147	28	2.65
Q-A5	402	1383	390	13,428	12,044	871	12	2.14
Q-A6	418	7190	389	95,047	87,857	1222	29	3.89
Q-B1	436	2718	425	5092	2375	87	11	1.25
Q-B2	424	2619	411	4951	2332	89	13	1.41
Q-B3	410	2156	399	3238	1082	50	11	0.85
Q-B4	411	3262	386	7366	4104	126	25	2.53
Q-B5	386	9456	375	53,911	44,456	470	11	2.74
Q-B6	405	8995	386	57,519	48,524	539	19	4.56
C1-ref.	431	378,864	422	39,5815	16,951	4	8	1.00
**Sensor**	**λ_max-BEFORE_ [nm]**	**Intensity λ_max-BEFORE_ [a.u.]**	**λ_max-AFTER_ [nm]**	**Intensity @λ_max-AFTER_ [a.u.]**	**|ΔI_max_| [a.u.]**	**ΔI_max_^a^ [%]**	**Δλ_max_ [nm]**	**Relative Sensitivity ^c^**
**Cationic photopolymerisation process of TEGDVE monomer under 320 nm**								
Q-REF	423	3047	437	10,841	20,727	680	14	0.01
Q-A1	431	701	432	3485	2784	397	1	0.01
Q-A2	439	506	441	2736	2229	440	−2	0.00
Q-A3	437	514	437	2389	1875	365	−1	0.00
Q-A4	414	662	386	6548	5885	889	−28	2.13
Q-A5	413	1195	391	23,632	22,437	1878	−22	2.65
Q-A6	414	1295	391	14,775	13,480	1041	−23	3.38
Q-B1	439	975	440	4958	3983	408	1	0.46
Q-B2	432	753	422	3736	2984	396	−9	0.32
Q-B3	420	672	426	2649	1977	294	6	0.09
Q-B4	420	861	386	4448	3587	417	−34	1.78
Q-B5	407	1436	386	14,149	12,713	885	−20	2.88
Q-B6	403	2298	386	24,556	22,258	969	−17	4.74
25ST -ref.	448	132184	438	114,582	17,602	13	−10	1.00

^a^ changes in fluorescence intensity expressed as a percentage in relation to the initial value before polymerisation. ^b^ Relative sensitivity S_rel_ as a reference sensor was used Coumarin 1. ^c^ Relative sensitivity S_rel_ as a reference sensor was used 25ST probe.

**Table 3:** Functional group conversions of vinyl monomer for TEGDVE and epoxy monomer for S105 and using photoinitiating system based on diphenyliodonium hexafluorophosphate (HIP 1 wt.%) and 1,6-diphenylquinolin-2-one derivatives (0.1 wt.%) in the role of photosensitizers at 365 nm exposure.

Fluorescent Sensor/Photosensitizer	ε @365_ab-365_ [dm^3^·mol^-1^·cm^-1^]	*E*_ox_^1/2^ [mV]	*E*_00(S)_ [eV]	Δ*G*_et(S)_ [eV]	*E*_00 (T)_ [eV]	Δ*G*_et (T)_ [eV]	^1^ Conversion of TEGDVE @365 nm	^2^ Conversion of S105 @365 nm
1,6-diphenylquinolin-2-one -reference								
Q-REF	2966	1595	319	−1.07	2.61	−0.31	62.88	28.43
**SERIES A**								
Q-A1	3710	1379	309	−1.18	2.58	−0.50	86.23	51.22
Q-A2	3224	1519	315	−1.10	2.59	−0.23	90.68	42.21
Q-A3	2838	1589	318	−1.07	2.60	−0.39	0.07	34.86
Q-A4	2824	1663	322	−1.04	2.61	−0.64	20.97	23.11
Q-A5	3027	1675	323	−1.04	2.62	−0.24	73.43	27.91
Q-A6	3596	1678	323	−1.03	2.62	−0.25	86.62	31.26
**SERIES B**								
Q-B1	3366	1425	308	−1.12	2.54	−0.41	67.86	30.22
Q-B2	3169	1598	315	−1.03	2.57	−0.27	40.82	30.34
Q-B3	2062	1673	318	−0.98	2.58	−0.21	1.90	17.28
Q-B4	2540	1763	323	−0.95	2.63	−0.16	40.82	15.62
Q-B5	1863	1778	324	−0.94	2.61	−0.13	20.94	15.16
Q-B6	2751	1773	324	−0.95	2.61	−0.13	74.61	15.09

*E*_ox_^1/2^—the electrochemically determined oxidation half-wave potentials (vs. Ag/AgCl) of the photosensitizer (the electron donor). *E*_red_^1/2^—the electrochemically determined reduction half-wave potentials (vs. Ag/AgCl) of the HIP (the electron acceptor) - E_red_^1/2^_HIP_ = -0.68V vs. SCE (-0.72V vs. Ag/AgCl) [38,39]. *E*_00_—the excitation energy of the co-initiator, which is referred to as singlet excitation energy. Δ*G*_et_—the enthalpy of free electron transfer (calculated from the Rhema-Weller formula). ^1^ conversion was calculated using the real-time FT-IR. ^2^ conversion was calculated using the Photo-DSC.

## Supplementary Materials

The following are available online at https://www.mdpi.com/2073-4360/11/11/1756/s1: Scheme S1–S2: Preparation and characterization of quinolin-2-one derivatives, Table S1: Spectroscopic characterization of quinolin-2-one derivatives. Figure S1–S26: ^1^H NMR and ^13^C NMR spectra of synthesized compounds. Figure S27–S39: Emission and excitation spectra for the determination of the excited singlet state energy for investigated of 1,6-diphenylquinolin-2-one derivatives in acetonitrile. Figure S40–S53: Applicability of 1,6-diphenylquinolin-2-one derivatives for on-line monitoring progress of free-radical photopolymerisation processes. Figure S54–S67: Applicability of 1,6-diphenylquinolin-2-one derivatives for on-line monitoring progress of thiol-ene photopolymerisation processes. Figure S68–S81: Applicability of 1,6-diphenylquinolin-2-one derivatives for on-line monitoring progress of cationic photopolymerisation processes. Figure S82–S94: Cyclic voltammetry curves showing oxidation processes of 1,6-diphenylquinolin-2-one derivatives in acetonitrile. Table S2: The optimized structures and HOMO and LUMO orbitals of investigated 1,6-diphenylquinolin-2-one derivatives free molecules determined with the use of uB3LYP/6-31G* level of theory. Figure S95–S107: Applicability of the 1,6-diphenylquinolin-2-one for on-line monitoring progress of cationic photopolymerisation of vinyl monomer.
