# Supplementary material for: Applicability of 1,6-Diphenylquinolin-2-one Derivatives as Fluorescent Sensors for Monitoring the Progress of Photopolymerisation Processes and as Photosensitisers for Bimolecular Photoinitiating Systems
Source: Polymers (Basel). 2019 Oct 25;11(11):1756. doi: 10.3390/polym11111756 (PMC6918307; doi:10.3390/polym11111756)
Supplement: Supplementary file 1 [file polymers-11-01756-s001.pdf]

Supplementary Materials

Applicability of 1,6-Diphenylquinolin-2-one Derivatives as Fluorescent Sensors for Monitoring the Progress of Photopolymerisation Processes and as Photosensitisers for Bimolecular Photoinitiating Systems

Monika Topa [1], Filip Petko [2], Mariusz Galek [2], Kamil Machowski [2], Maciej Pilch [1], Patryk Szymaszek [1] and Joanna Ortyl [1,2,*]

[1] Faculty of Chemical Engineering and Technology, Cracow University of Technology, Warszawska 24, 30-155 Cracow, Poland; mtopa@chemia.pk.edu.pl (M.T.); pilchmac@gmail.com (M.P.); szymaszek.patryk@gmail.com (P.S.)

[2] Photo HiTech Ltd., Bobrzyńskiego 14, 30-348 Cracow, Poland; filip.petko@photohitech.com (F.P.); mariusz.galek@photohitech.com (M.G.); kamil.machowski@photohitech.com (K.M.)

* Correspondence: jortyl@chemia.pk.edu.pl; Tel.: +48-(12)628-31-36

Table of contents:

1. Preparation and characterization of quinolin-2-one derivatives ………………. **S3**
1.1. Materials and methods …………………………………………………………... **S3**
1.2. Synthesis of quinolin-2-one derivatives……………………...…………………... **S3**

2. Spectroscopic properties of the 1,6-diphenylquinolin-2-one derivatives……… **S21**
2.1. Emission and excitation spectra for the determination of the excited singlet state energy for investigated of 1,6-diphenylquinolin-2-one derivatives in acetonitrile ……………………………………………………………………… **S21**

3. Applicability of 1,6-diphenylquinolin-2-one derivatives for on-line monitoring progress of photopolymerization processes by FPT method …… **S24**
3.1. Applicability of 1,6-diphenylquinolin-2-one derivatives for on-line monitoring progress of free-radical photopolymerization processes ………… **S24**
3.2. Applicability of 1,6-diphenylquinolin-2-one derivatives for on-line monitoring progress of thiol-ene photopolymerization processes ………….. **S28**
3.3. Applicability of 1,6-diphenylquinolin-2-one derivatives for on-line monitoring progress of cationic photopolymerization processes …………….. **S31**

4. Cyclic voltammetry curves showing oxidation processes of 1,6-diphenyl-quinolin-2-one derivatives in acetonitrile ……………………………………… **S35**

5. The optimized structures and HOMO and LUMO orbitals of investigated 1,6-diphenylquinolin-2-one derivatives free molecules determined with the use of uB3LYP/6-31G* level of theory ………………………………………………. **S37**

6. Applicability of the 1,6-diphenylquinolin-2-one for on-line monitoring progress of cationic photopolymerization of vinyl monomer ………………… **S41**

1. Preparation and Characterization of Quinolin-2-one Derivatives

1. 1. Materials and Methods

Reagents for synthesis were purchased from Sigma-Aldrich or Alfa Aesar and used without further purification.

Structure and purity of obtained products were confirmed by NMR and LC-MS analysis. ^{1}H NMR and ^{13}C NMR spectra were recorded in *d*$_6$-DMSO on JNM-ECZR500 RS1 500 MHz (JEOL) spectrometer. Chemical shifts are reported in parts per million (δ) and referenced to residual protonated solvent peak (δ = 2.50 ppm).

LC-MS analyses were obtained on Shimadzu LCMS-2020 with ESI ionization method. Acetonitrile was used as eluent.

1. 2. Synthesis of Quinolin-2-one Derivatives

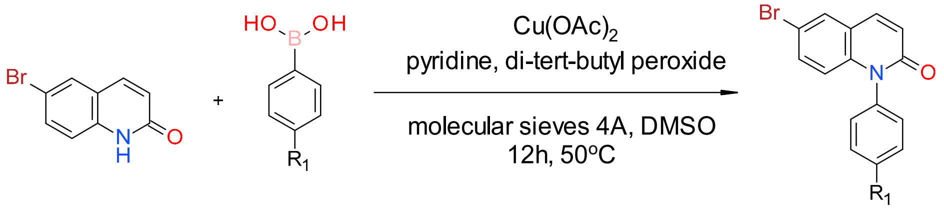

Where: R_1 = -OCH_3, -CN

Synthetic procedure of step 1

6-bromoquinolin-2-one (22 mmol, 1eq), the corresponding phenylboronic acid derivative (56 mmol, 2.5 eq), $Cu(OAc)_2$ (4.070 g, 22 mmol, 1eq), pyridine (3.810 g, 48 mmol , 2,15eq), di-tert-butyl peroxide (19,680 g, 135 mmol, 6eq), 4A molecular sieves (8,300 g) and DMSO (200 ml) were mixed together and heated at 50 ° C for 12 hours. The cooled reaction mixture was filtered through Celite, to the filtrate water was added and extracted with ethyl acetate. The combined organic layers were washed with brine, dried over Na_2SO_4 and purified by column chromatography (SiO_2, hexane / ethyl acetate), purity confirmed by TLC analysis using a hexane/ethyl acetate eluent. Yields in the range of 80 (for derivative with R_1 = OCH_3) and 95% (for derivative with R_1 = CN) were obtained.

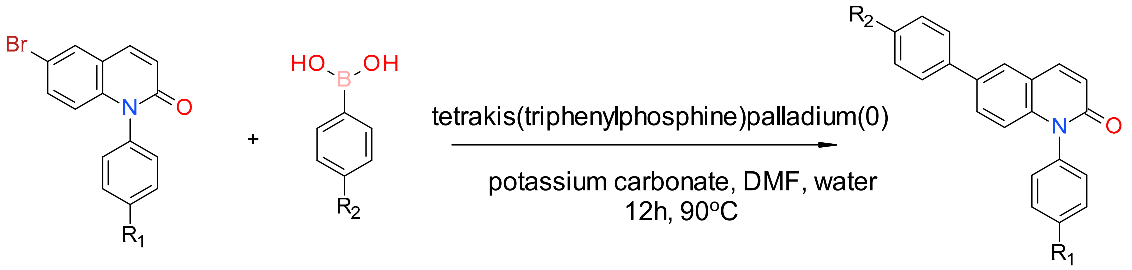

Where: R_1 = -OCH_3, -CN

R_2 = -OCH_3, -CH_3, -SCH_3, -CF_3, -F, -SO_2CH_3, -CN, -H

Synthetic procedure of step 2

Product from the previous step (2.7 mmol, 1eq), corresponding phenylboronic acid (3.2 mmol, 1.2eq), tetrakis(triphenylphosphine)palladium(0) (0.190 g, 0.2 mmol), 0.06eq) K_2CO_3 (1.1110 g, 8 mmol, 3eq), DMF (12 mL), H_2O (1 mL) were mixed and heated for 12 hours at 90 ° C. Then water was added and extracted with ethyl acetate. The combined organic layers were washed with brine, dried over Na_2SO_4, the solvent was evaporated. Product was purified by column chromatography (SiO_2, hexane / ethyl acetate) orthe residue was crystallized from methanol (yield of 50 to 90%).

STRUCTURE	SPECTROSCOPIC DATA	YIELD
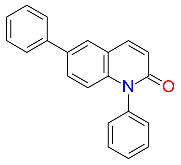<br>**Q-REF**	*1,6-difenylochinolin-2-one*<br>**^{1}H NMR (500 MHz, d_6-DMSO) δ [ppm]** 8,12-8,11 (d, J = 4,92 Hz, 1H), 8,11-8,10 (d, J = 6,90 Hz), 7,75-7,72 (dd, J = 8,84, 2,22 Hz, 1H), 7,69-7,63 (m, 4H), 7,59-7,55 (tt, J = 7,42, 2,22 Hz, 1H), 7,48-7,45 (t, 7,77 Hz, 2H), 7,38-7,34 (m, 3H), 6,74-6,72 (d, J = 9,53 Hz, 1H), 6,59-6,58 (d, J = 8,80 Hz, 1H)<br><br>**^{13}C NMR (125 MHz, *d_6*-DMSO) δ [ppm]** 161,6; 140,9; 140,7; 139,4; 138,1; 134,6; 130,6; 129,55; 129,54; 129,49; 129,3; 128,0; 127,0; 126,9; 122,5; 120,8; 116,4<br><br>MS (ESI) m/z(%): 298 ($[M+H]^+$, 99%)	90%
SERIE A		
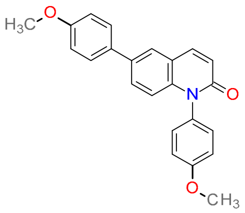<br>**Q-A1**	*1,6-bis(4-methoxyphenyl)quinolin-2-one*<br>**^{1}H NMR (500 MHz, d_6-DMSO) δ [ppm]** 8,08-8,05 (d, J = 9,67 Hz, 1H), 8,03-8,03 (d, J = 2,20 Hz, 1H), 7,71-7,68 (dd, J = 8,85, 2,20 Hz, 1H), 7,64-7,60 (m, 2H), 7,27-7,23 (m, 2H), 7,18-7,14 (m, 2H), 7,05-7,01 (m, 2H), 6,71 (d, J = 9,61 Hz, 1H), 6,63-6,61 (d, J = 9,00 Hz, 1H), 3,85 (s, 3H), 3,79 (s, 3H)<br><br>**^{13}C NMR (125 MHz, *d_6*-DMSO) δ [ppm]** 161,8; 159,7; 159,4; 140,7; 140,6; 134,3; 131,8; 130,6; 130,5; 129,1; 128,1; 126,1; 122,4; 120,8; 116,5; 115,7; 115,0; 56,0; 55,7<br><br>MS (ESI) m/z(%): 258 ($[M+H]^+$, 100%)	85%

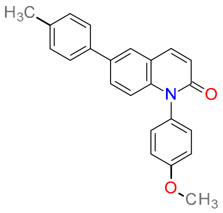<br>**Q-A2**	*1-(4-methoxyphenyl)-6-(4-methylphenyl)quinolin-2-one*<br>**^{1}H NMR (500 MHz, d_6-DMSO) δ [ppm]** 8,09-8,07 (m, 2H), 7,74-7,71 (dd, J = 8,74, 2,00 Hz, 1H), 7,60-7,58 (d, J = 8,12 Hz, 2H), 7,29-7,27 (d, J = 8,10 Hz, 2H), 7,27-7,24 (m, 2H), 7,17-7,15 (m, 2H), 6,71-6,69 (d, J = 9,70 Hz, 1H), 6,65-6,62 (d, J = 8,85 Hz, 1H), 3,82 (s, 3H), 2,30 (s, 3H)<br><br>**^{13}C NMR (125 MHz, *d_6*-DMSO) δ [ppm]**161,8; 159,7; 140,9; 140,8; 137,3; 136,5; 134,4; 130,5; 130,4; 130,1; 129,2; 126,8; 126,5; 122,5; 120,8; 116,5; 115,7; 56,0; 21,2<br><br>MS (ESI) m/z(%): 342 ([M+H]$^{+}$, 95%)	74%
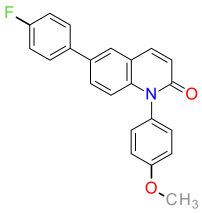<br>**Q-A3**	*1-(4-methoxyphenyl)-6-(4-fluorophenyl)quinolin-2-one*<br>**^{1}H NMR (500 MHz, d_6-DMSO) δ [ppm]** 8,09-8,06 (m, 2H), 7,74-7,71 (m, 3H), 7,33-7,29 (m, 2H, 7,27-7,25 (m, 2H), 7,17-7,15 (m, 2H), 6,73-6,70 (d, J = 9,60 Hz, 1H), 6,65-6,63 (d, J = 8,76 Hz, 1H), 3,82 (s, 3H)<br><br>**^{13}C NMR (125 MHz, *d_6*-DMSO) δ [ppm]** 163,3; 161,8; 161,4; 159,7; 141,0; 140,7; 136,0; 135,9; 133,5; 130,5; 130,49; 129,4; 129,0; 128,95; 126,8; 122,6; 120,8; 116,5; 116,4; 116,2; 115,7; 56,0<br><br>MS (ESI) m/z(%): 346 ([M+H]$^{+}$, 99%)	50%
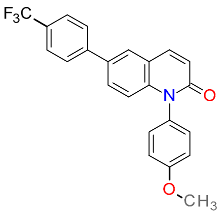<br>**Q-A4**	*1-(4-methoxyphenyl)-6-[4-(trifluoromethyl)phenyl)]quinolin-2-one*<br>**^{1}H NMR (500 MHz, d_6-DMSO) δ [ppm]** 8,21-8,20 (d, J = 2,12 Hz, 1H), 8,12-8,09 (d, J = 9,60 Hz, 1H), 7,93-7,91 (d, J = 8,24 Hz, 2H), 7,84-7,81 (m, 3H), 7,28-7,26 (m, 2H), 7,18-7,16 (m, 2H), 6,75-6,73 (d, J = 9,60 Hz, 1H), 6,70-6,68 (d, J = 9,00 Hz, 1H), 3,82 (s, 3H)<br><br>**^{13}C NMR (125 MHz, *d_6*-DMSO) δ [ppm]** 161,8; 159,8; 143,4; 141,7; 140,7; 132,7; 130,5; 130,4; 129,6; 128,4; 128,1; 127,7; 127,5; 126,4; 126,3; 126,0; 123,8; 122,7; 120,8; 116,7; 115,8; 56,0<br><br>MS (ESI) m/z(%): 396 ([M+H]$^{+}$, 98%)	55%
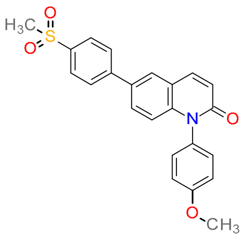<br>**Q-A5**	*1-(4-methoxyphenyl)-6-(4-metylsulfonylfenylo)quinolin-2-one*<br>**^{1}H NMR (500 MHz, d_6-DMSO) δ [ppm]** δ 8,23-8,23 (d, J = 1,93 Hz, 1H), 8,12-8,10 (d, J = 9,74 Hz, 1H), 8,03-7,98 (m, 2H), 8,01-7,96 (m, 2H), 7,85-7,83 (dd, J = 12,30, 2,10 Hz, 1H), 7,28-7,26 (m, 2H), 7,18-7,16 (m, 2H), 6,76-6,73 (d, J = 9,80 Hz, 1H), 6,70-6,68 (d, J = 8,90 Hz, 1H), 3,82 (s, 3H), 3,22 (s, 3H)	85%

	**^{13}C NMR (125 MHz, *d_6*-DMSO) δ [ppm]** 161,8; 159,8; 144,4; 141,9; 140,7; 140,0; 132,5; 130,5; 1530,4; 129,7; 128,3; 127,8; 127,7; 122,8; 120,9; 116,7; 115,8; 56,0; 41,1<br><br>MS (ESI) m/z(%): 406 ([M+H]$^+$, 100%)	
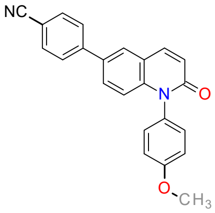<br>**Q-A6**	*1-(4-methoxyphenyl)-6-(4-nitrylofenylo)quinolin-2-one*<br>**^{1}H NMR (500 MHz, d_6-DMSO) δ [ppm]** 8,22-8,22 (d, J = 1,60 Hz, 1H), 8,10-8,08 (d, J = 9,60 Hz, 1H), 7,94-7,92 (m, 2H), 7,90-7,88 (m, 2H), 7,84-7,81 (dd, J = 8,70, 1,60 Hz, 1H), 7,27-7,25 (m, 2H), 7,18-7,15 (m, 2H), 6,75-6,73 (d, J = 9,60 Hz, 1H), 6,69-6,67 (d, J = 8,94 Hz, 1H), 3,86 (s, 3H)<br><br>**^{13}C NMR (125 MHz, *d_6*-DMSO) δ [ppm]**161,8; 159,8; 143,9; 141,9; 140,7; 133,4; 132,3; 130,5; 130,4; 129,5; 127,7; 127,6; 122,8; 120,8; 119,4; 116,7; 115,8; 110,4; 56,0<br><br>MS (ESI) m/z(%): 353 ([M+H]$^+$, 99%)	86%
	SERIE B	
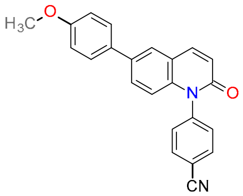<br>**Q-B1**	*1-(4-cyanophenyl)-6-(4-methoxyphenyl)quinolin-2-one*<br>**^{1}H NMR (500 MHz, d_6-DMSO) δ [ppm]** 8,14-8,11 (m, 3H), 8,08-8,07 (d, J = 2,30 Hz, 1H), 7,71-7,69 (dd, J = 8,90, 2,27 Hz, 1H), 7,66-7,62 (m, 4H), 7,05-7,03 (m, 2H), 6,74-6,72 (d, J = 9,40 Hz, 1H), 6,57-6,55 (d, J = 8,90 Hz, 1H), 3,75 (s, 3H)<br><br>**^{13}C NMR (125 MHz, *d_6*-DMSO) δ [ppm]** 161,3; 159,5; 142,5; 141,3; 139,6; 134,8; 134,7; 131,7; 131,2; 129,3; 128,1; 126,4; 122,3; 120,8; 118,9; 116,2; 115,0; 112,4; 55,7<br><br>MS (ESI) m/z(%): 353 ([M+H]$^+$, 100%)	76%
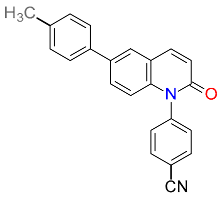<br>**Q-B2**	*1-(4-cyanophenyl)-6-(4-methylphenyl)quinolin-2-one*<br>**^{1}H NMR (500 MHz, d_6-DMSO) δ [ppm]** 8,10-8,06 (m, 4H), 7,69-7,66 (dd, J = 8,85, 2,07 Hz, 1H), 7,61-7,59 (m, 2H), 7,55-7,54 (m, 2H), 7,25-7,23 (d, J = 8,14 Hz, 2H), 6,70-6,68 (d, J = 9,50 Hz, 1H), 6,549-6,52 (d, J = 8,78 Hz, 1H), 2,30 (s, 3H)<br><br>**^{13}C NMR (125 MHz, *d_6*-DMSO) δ [ppm]** 161,4; 142,5; 141,3; 139,8; 137,4; 136,4; 134,8; 134,8; 134,7; 131,2; 130,2; 129,4; 128,6; 126,7; 122,3; 120,8; 118,9; 116,2; 112,4; 21,2<br><br>MS (ESI) m/z(%): 337 ([M+H]$^+$, 98%)	82%
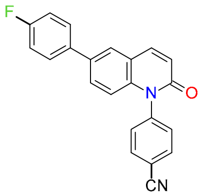<br>**Q-B3**	*1-(4-cyanophenyl)-6-(4-fluorophenyl)quinolin-2-one*<br>**^{1}H NMR (500 MHz, d_6-DMSO) δ [ppm]** 8,14-8,11 (m, 4H), 7,75-7,71 (m, 3H), 7,66-7,64 (m, 2H), 7,34-7,29 (m, 2H), 7,76-7,74 (d, J = 9,56 Hz, 1H), 6,60-6,57 (d, J = 8,86 Hz, 1H)<br><br>**^{13}C NMR (125 MHz, *d_6*-DMSO) δ [ppm]** 163,4; 161,5;	63%

	161,4; 142,4; 142,2; 140,0; 135,8; 135,75; 134,8; 133,9; 131,2; 129,6; 129,1; 129,0; 127,0; 122,4; 120,8; 118,9; 116,5; 116,3; 116,2; 112,4<br><br>MS (ESI) m/z(%): 341 ($[M+H]^+$, 99%)	
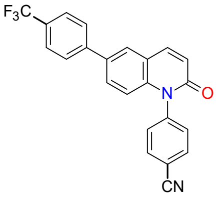<br><br>**Q-B4**	*1-(4-cyanophenyl)-6-(4-trifluoromethyl-phenyl)quinolin-2-one*<br>**^{1}H NMR (500 MHz, d_6-DMSO) δ [ppm]** 8,25-8,24 (d, J = 2,11 Hz, 1H), 8,16-8,12 (m, 3H), 7,94-7,92 (m, 2H), 7,85-7,89 (m, 3H), 7,67-7,65 (m, 2H), 6,78-6,76 (d, J = 9,64 Hz, 1H), 6,64-6,62 (d, J = 8,76 Hz, 1H)<br><br>**^{13}C NMR (125 MHz, *d_6*-DMSO) δ [ppm]** 161,4; 143,3; 143,25; 142,2; 141,2; 140,7; 133,2; 131,2; 129,8; 128,2; 127,8; 127,7; 126,5; 126,5; 126,0; 123,8; 122,6; 120,9; 118,9; 116,4; 112,5<br><br>MS (ESI) m/z(%): 391 ($[M+H]^+$, 100%)	59%
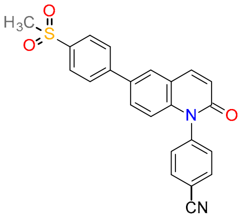<br><br>**Q-B5**	*1- (4-cyanophenyl) -6- (4-methylsulfonyl-phenyl)quinolin-2-one*<br>**^{1}H NMR (500 MHz, d_6-DMSO) δ [ppm]** 8,27-8,26 (d, J = 2,00 Hz, 1H), 8,16-8,13 (m, 3H), 8,03-8,01 (m, 2H), 7,98-7,96 (m, 2H), 7,84-7,82 (dd, J = 8,83, 2,20 Hz, 1H), 7,67-7,65 (m, 2H), 6,79-6,77 (d, J = 9,55 Hz, 1H), 6,64-6,63 (d, J = 8,83 Hz, 1H), 3,26 (s, 3H)<br><br>**^{13}C NMR (125 MHz, *d_6*-DMSO) δ [ppm]** 161,4; 144,2; 142,3; 141,2; 140,8; 134,8; 132,9; 131,2; 129,9; 128,3; 127,9; 127,8; 122,6; 120,9; 118,9; 116,5; 112,5; 44,1<br><br>MS (ESI) m/z(%): 401 ($[M+H]^+$, 99%)	81%
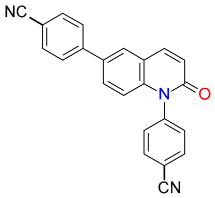<br><br>**Q-B6**	*1,6-bis (4-cyanophenyl)quinolin-2-one*<br>**^{1}H NMR (500 MHz, d_6-DMSO) δ [ppm]** 8,28-8,27 (d, J = 2,22 Hz, 1H), 8,15-8,13 (m, 3H), 7,97-7,91 (m, 4H), 7,85-7,82 (dd, J = 8,82, 2,30 Hz, 1H), 7,67-7,65 (m, 2H), 6,79-6,76 (d, J = 9,44 Hz, 1H), 6,64-6,62 (d, J = 8,77 Hz, 1H)<br><br>**^{13}C NMR (125 MHz, *d_6*-DMSO) δ [ppm]** 161,4; 143,3; 142,4; 140,7; 134,8; 133,2; 131,2; 129,8; 128,5; 128,2; 127,7; 127,6; 126,5; 126,4; 126,0; 123,8; 122,6; 120,9; 118,9; 116,4; 112,5<br><br>MS (ESI) m/z(%): 348 ($[M+H]^+$, 100%)	77%

^{1}HNMR and ^{13}CNMR spectra of synthesized compounds

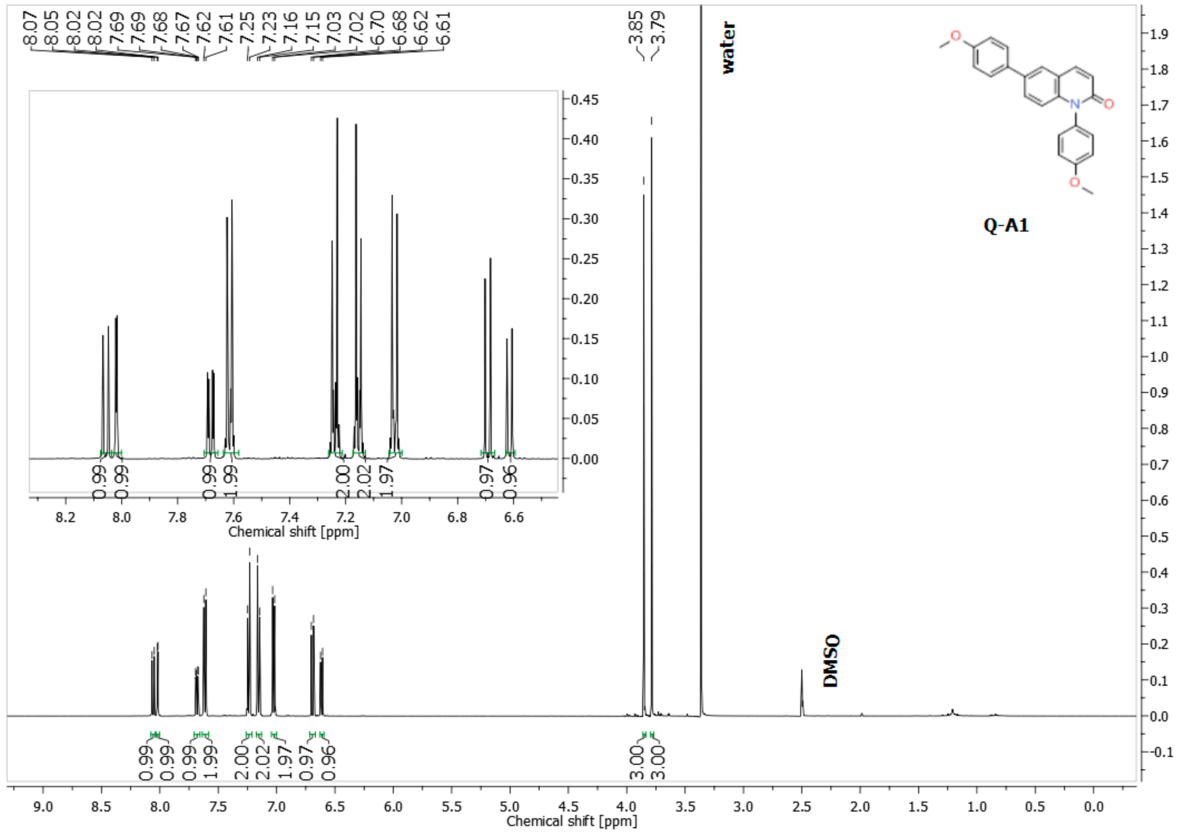

Figure S1. ^{1}HNMR of 1,6-bis(4-methoxyphenyl)quinolin-2-one (Q-A1).

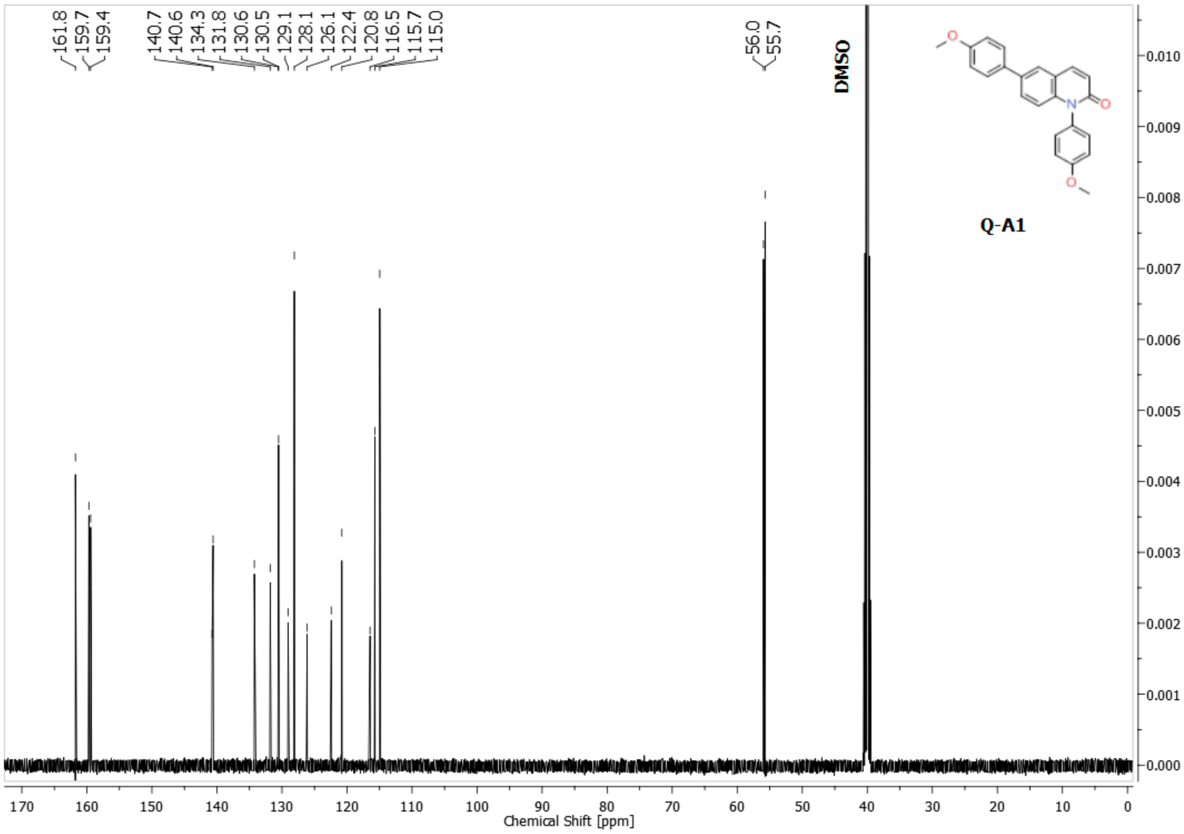

Figure S2. ^{13}CNMR of 1,6-bis(4-methoxyphenyl)quinolin-2-one (Q-A1).

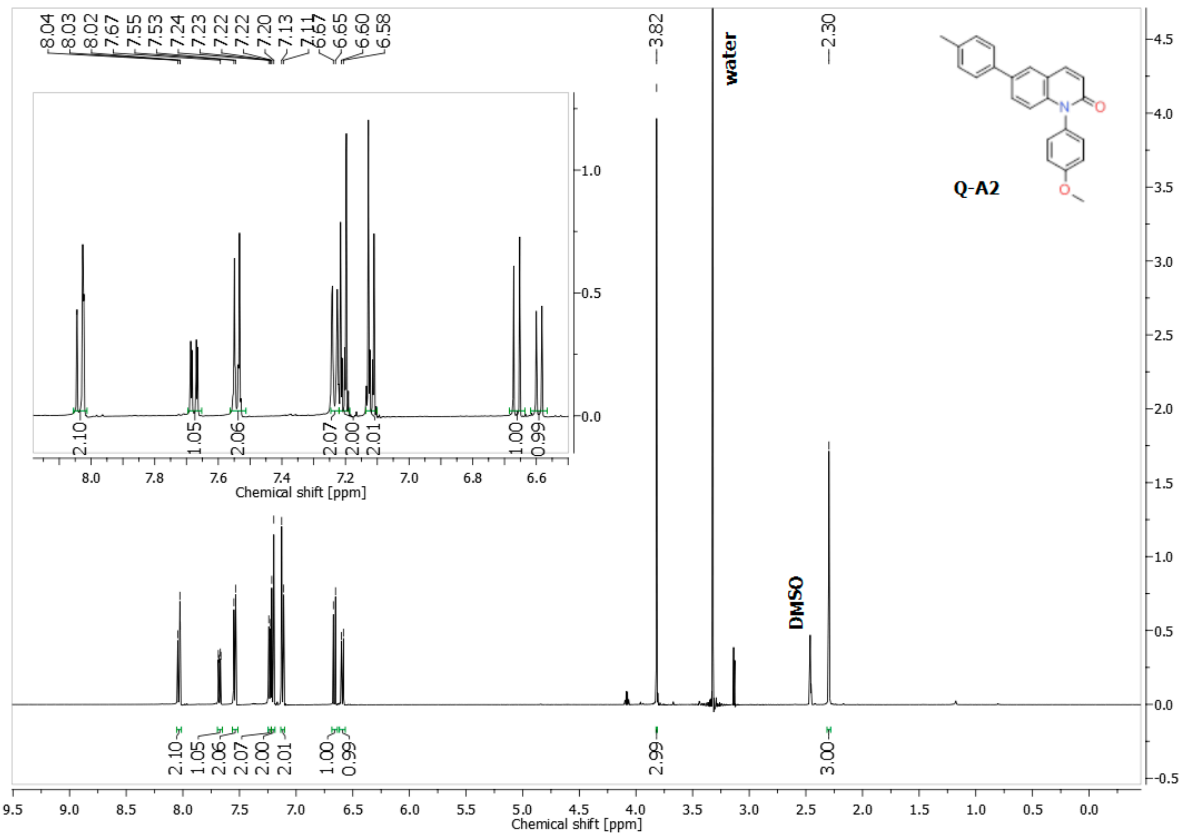

Figure S3: ^{1}HNMR of 1-(4-methoxyphenyl)-6-(4-methylphenyl)quinolin-2-one (Q-A2).

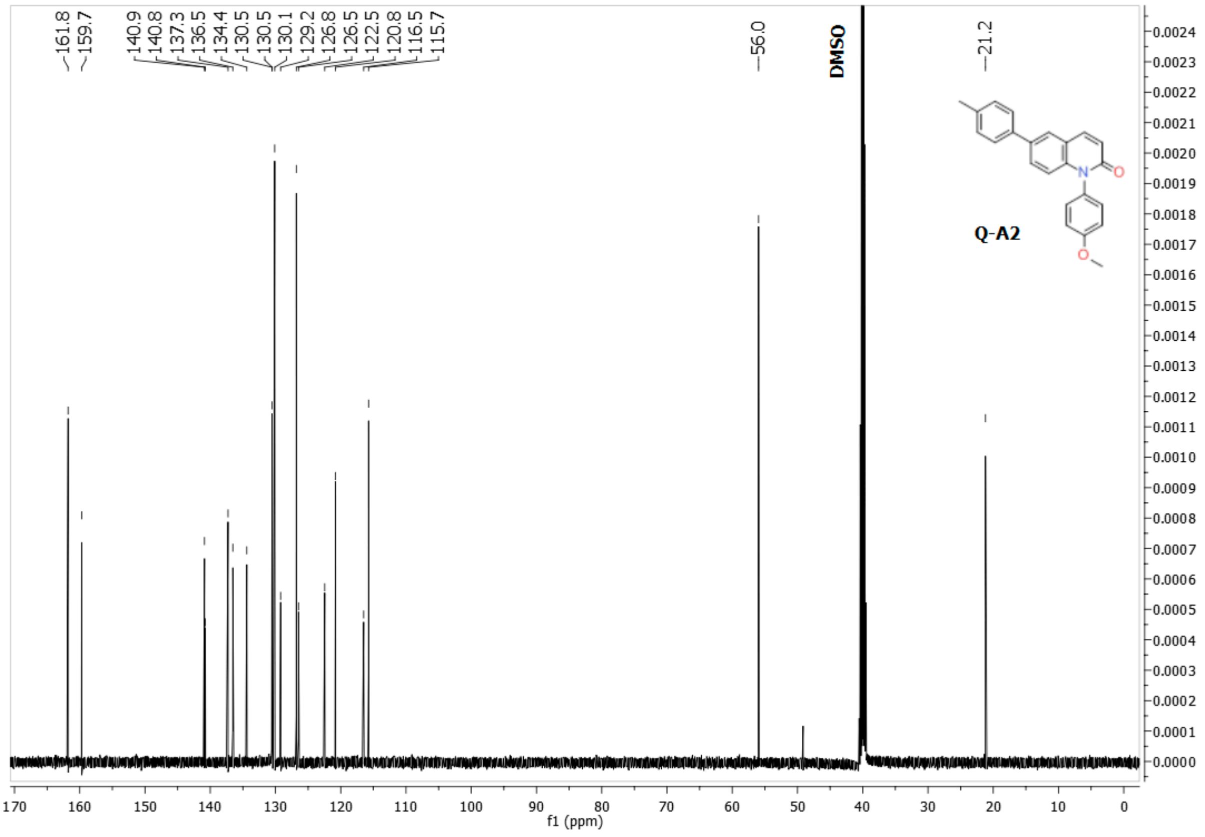

Figure S4. ^{13}CNMR of 1-(4-methoxyphenyl)-6-(4-methylphenyl)quinolin-2-one (Q-A2).

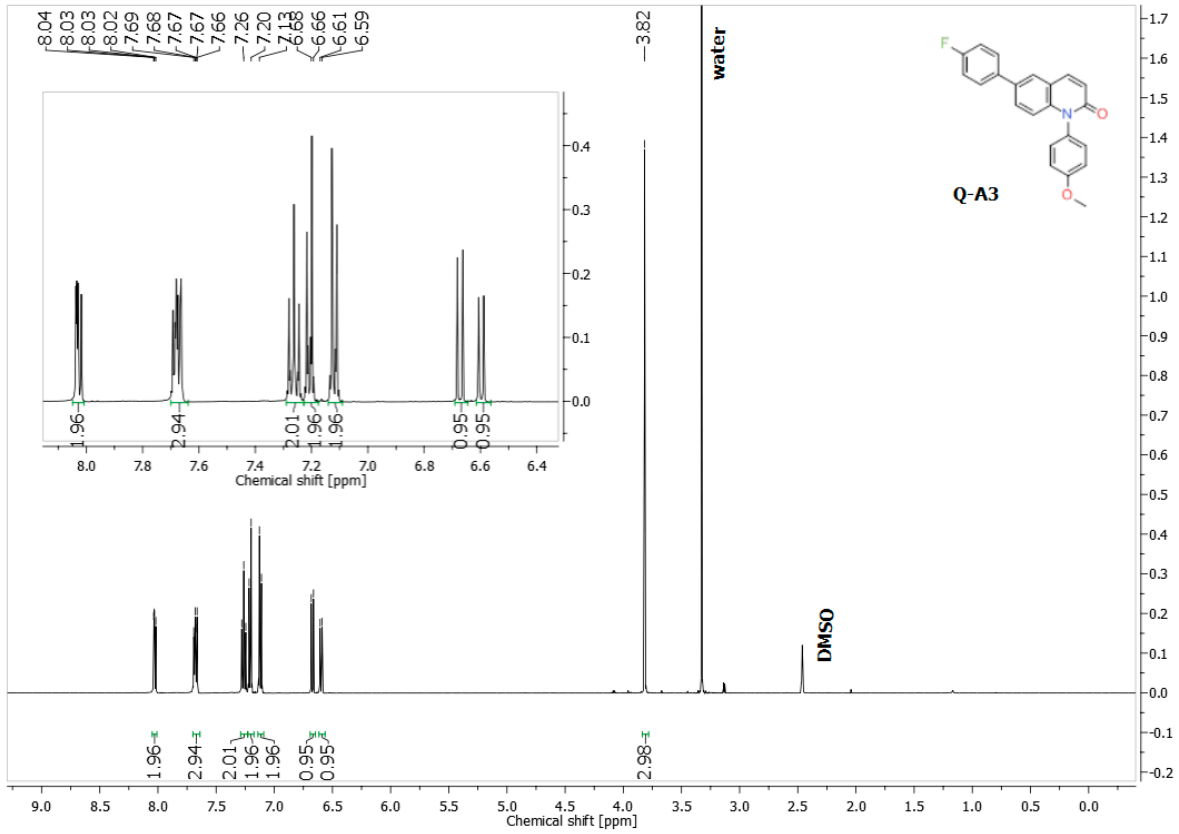

Figure S5. ^{1}HNMR of 1-(4-methoxyphenyl)-6-(4-fluorophenyl)quinolin-2-one (Q-A3).

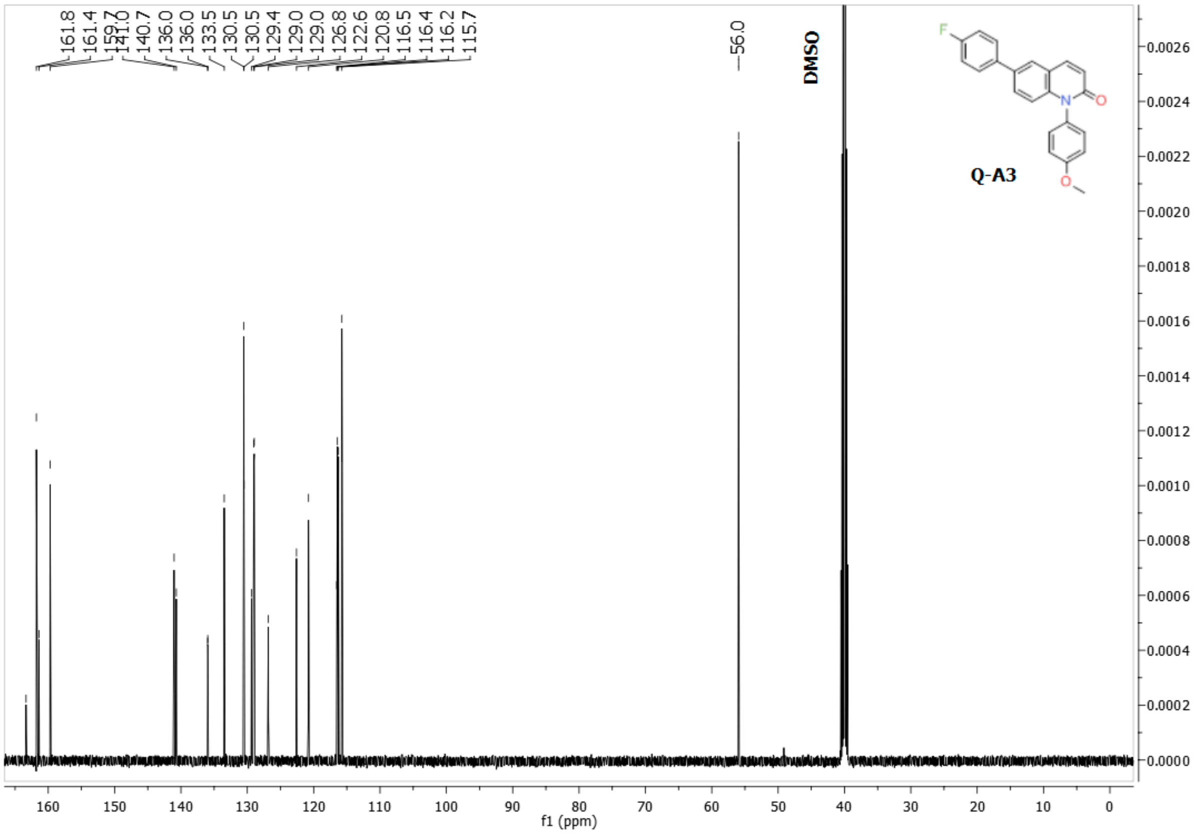

Figure S6. ^{13}CNMR of 1-(4-methoxyphenyl)-6-(4-fluorophenyl)quinolin-2-one (Q-A3).

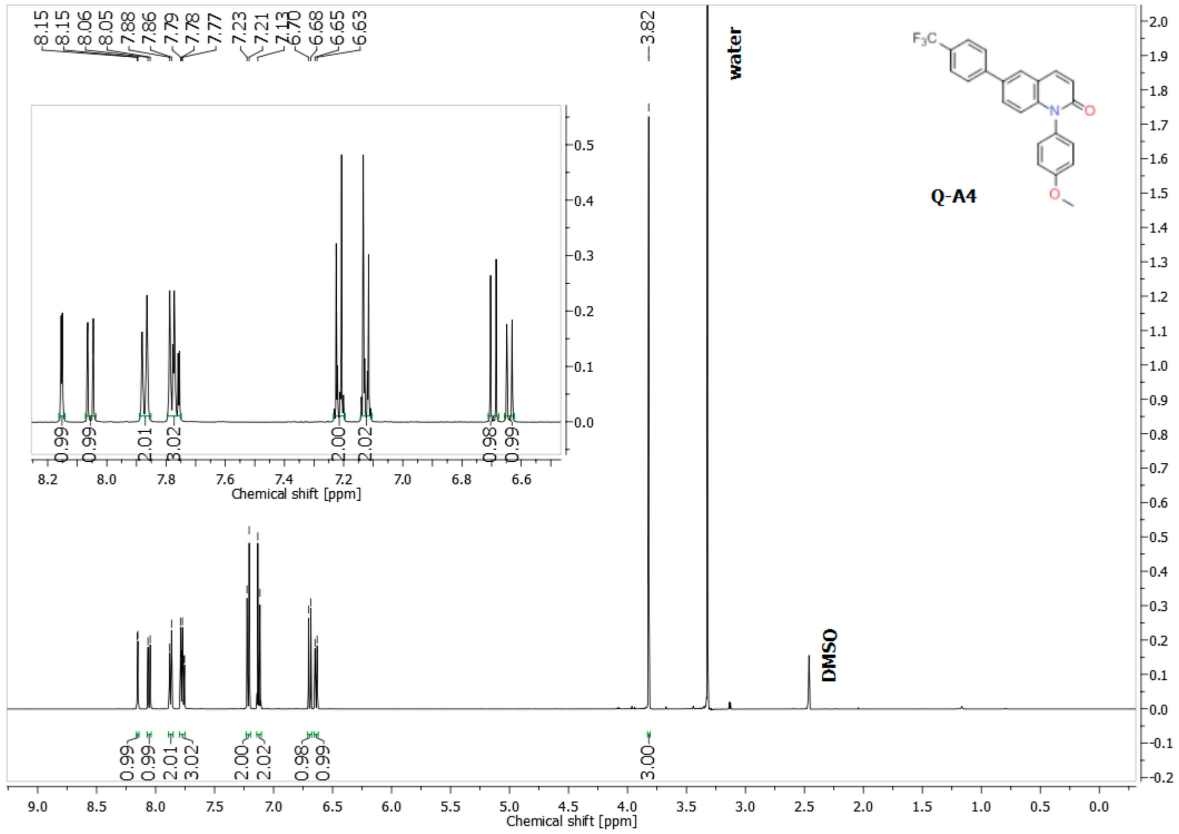

Figure S7. ^{1}HNMR of 1-(4-methoxyphenyl)-6-[4-(trifluoromethyl)phenyl)]quinolin-2-one (Q-A4).

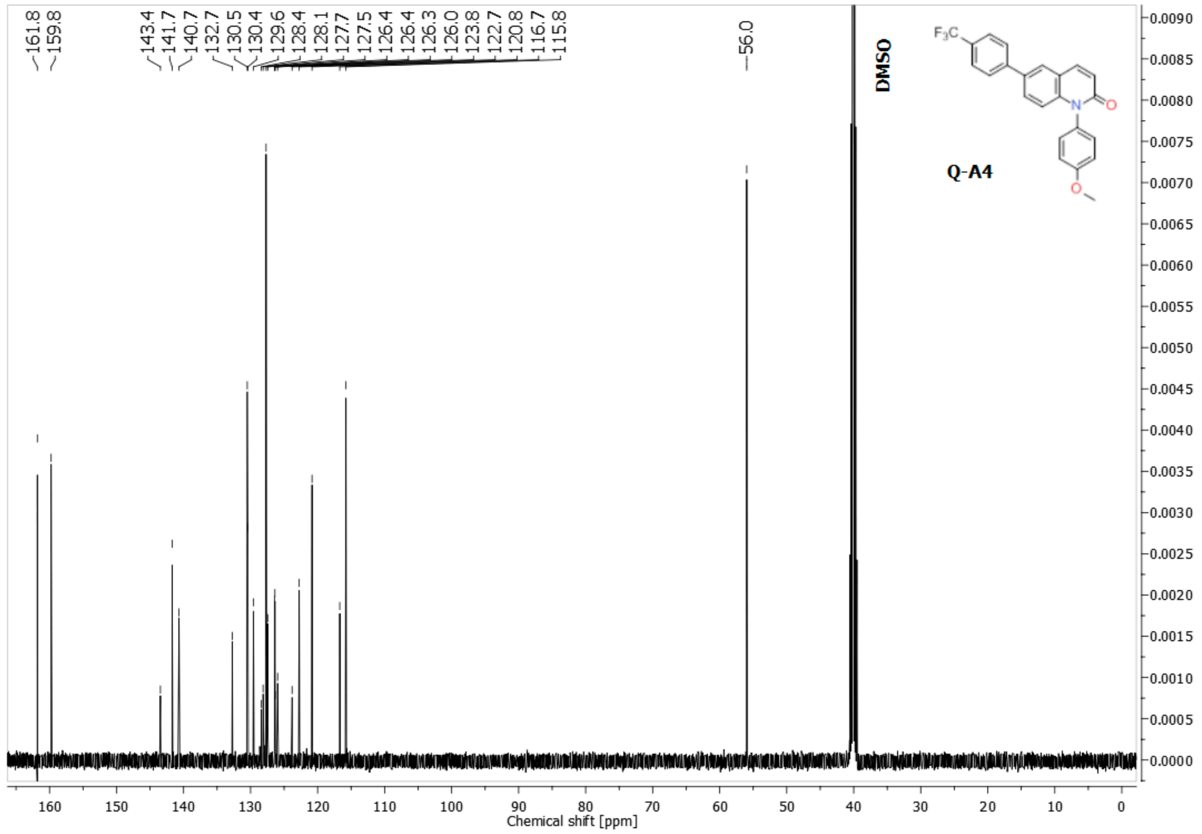

Figure S8. ^{13}CNMR of 1-(4-methoxyphenyl)-6-[4-(trifluoromethyl)phenyl)]quinolin-2-one (Q-A4).

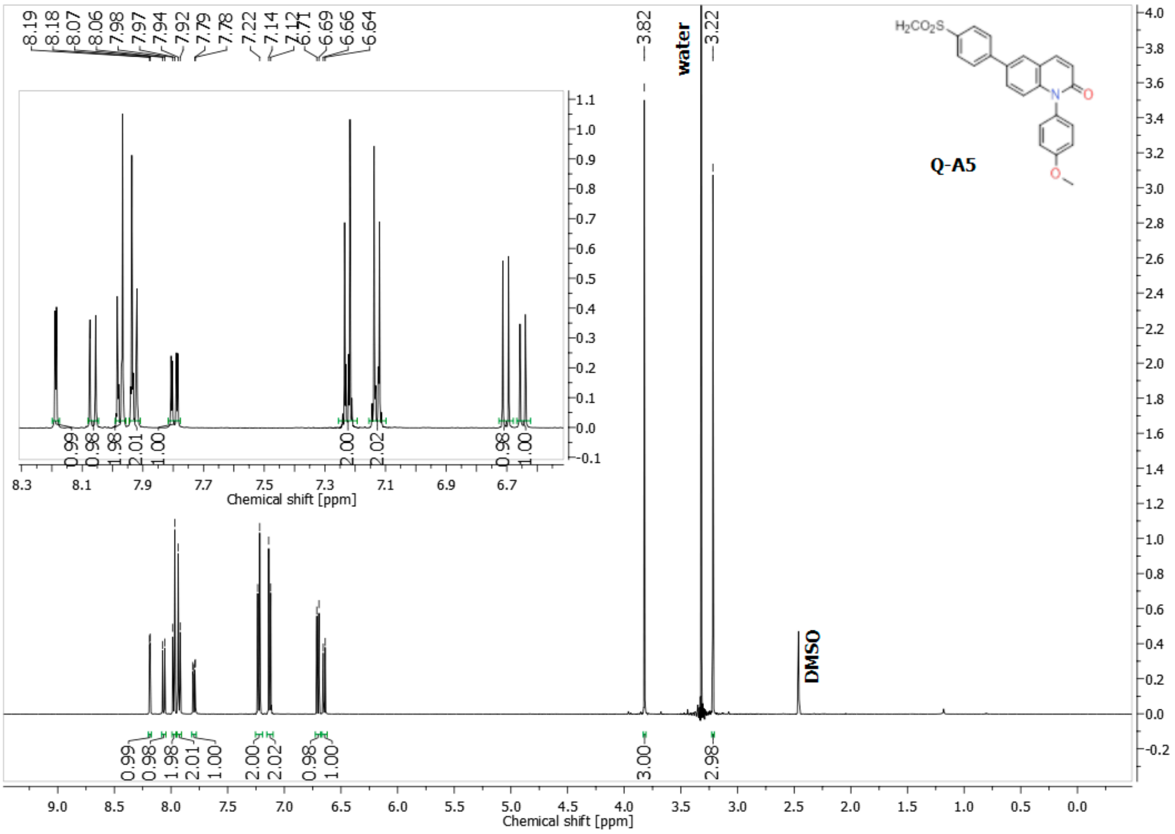

Figure S9. ^{1}HNMR of 1-(4-methoxyphenyl)-6-(4-metylsulfonylfenylo)quinolin-2-one (Q-A5).

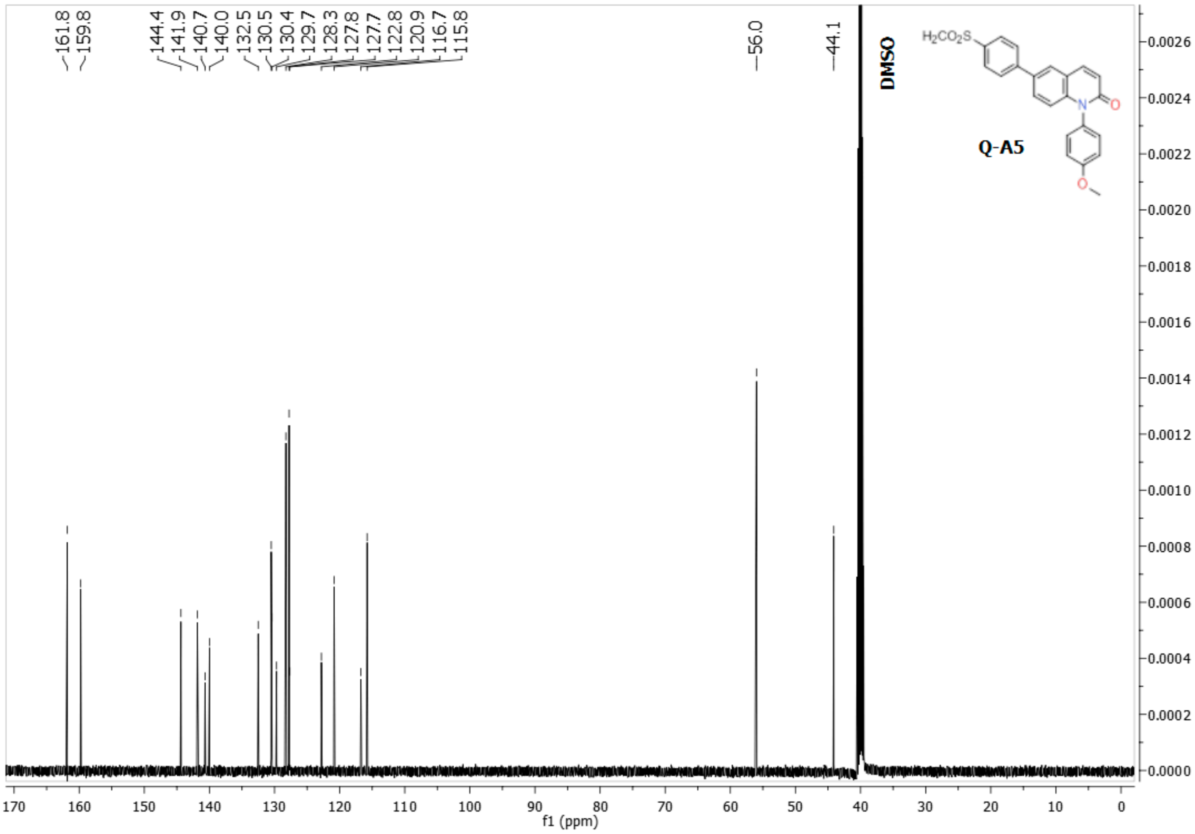

Figure S10. ^{13}CNMR of 1-(4-methoxyphenyl)-6-(4-metylsulfonylfenylo)quinolin-2-one (Q-A5).

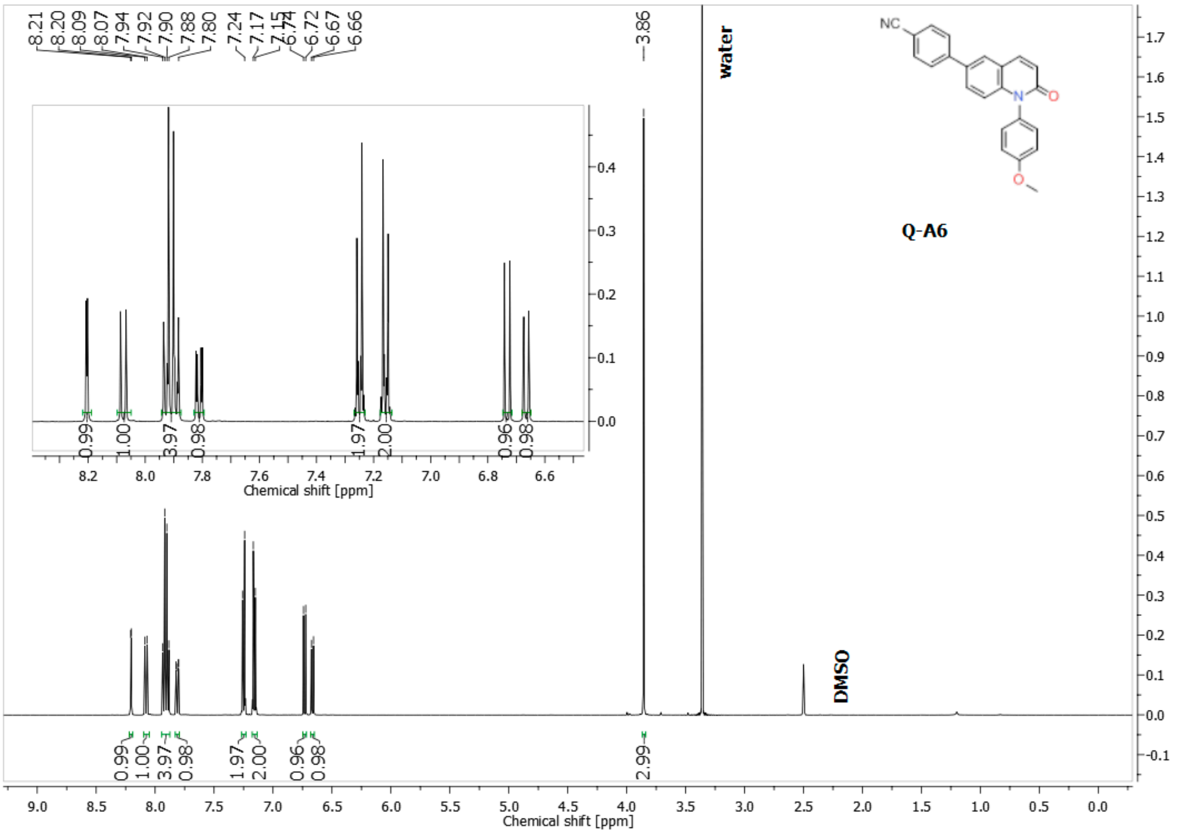

Figure S11. ^{1}HNMR of 1-(4-methoxyphenyl)-6-(4-nitrylofenylo)quinolin-2-one (Q-A6).

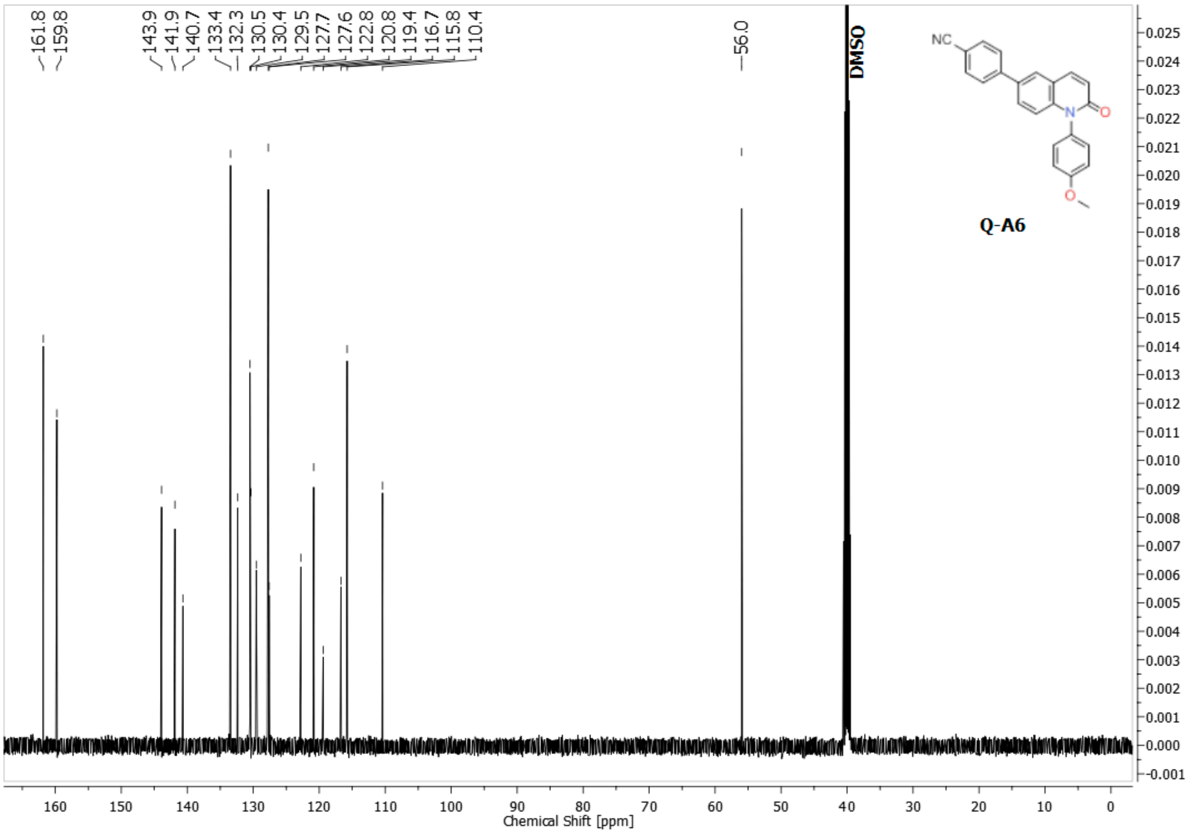

Figure S12. ^{13}CNMR of 1-(4-methoxyphenyl)-6-(4-nitrylofenylo)quinolin-2-one (Q-A6).

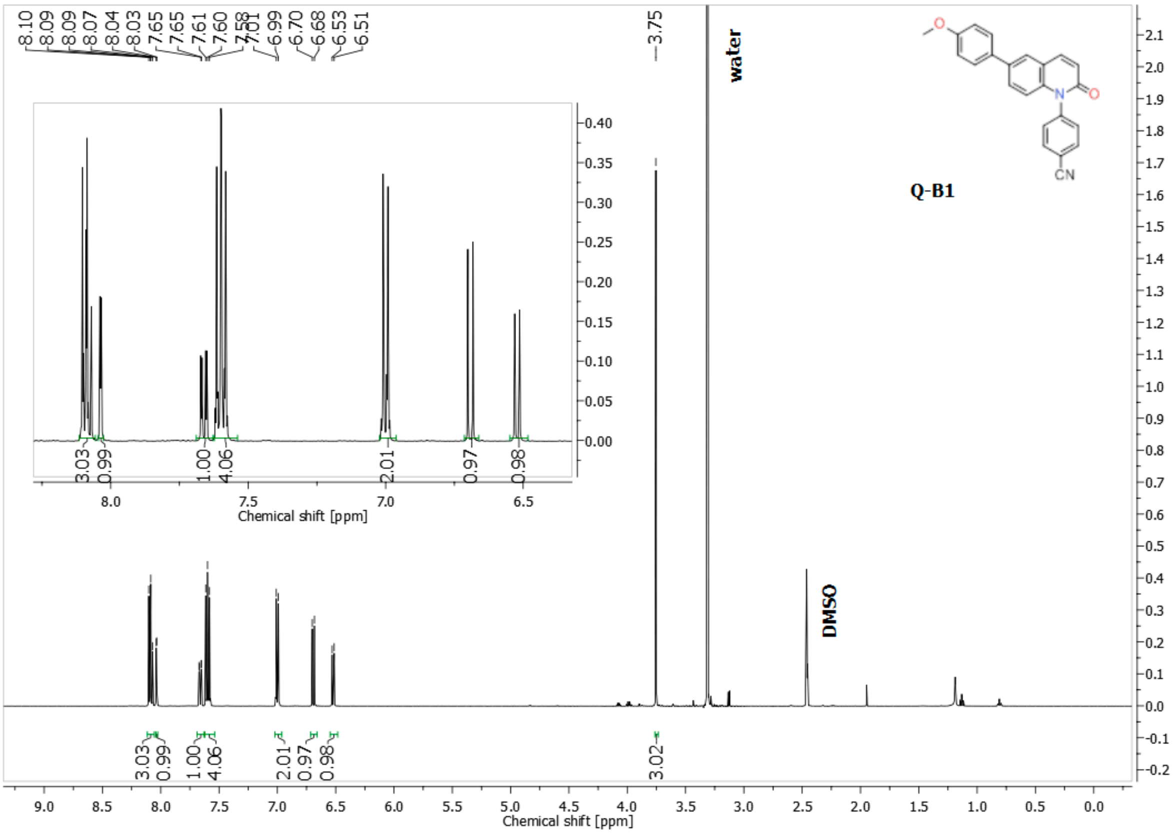

Figure S13. ^{1}HNMR of 1-(4-cyanophenyl)-6-(4-methoxyphenyl)quinolin-2-one (Q-B1).

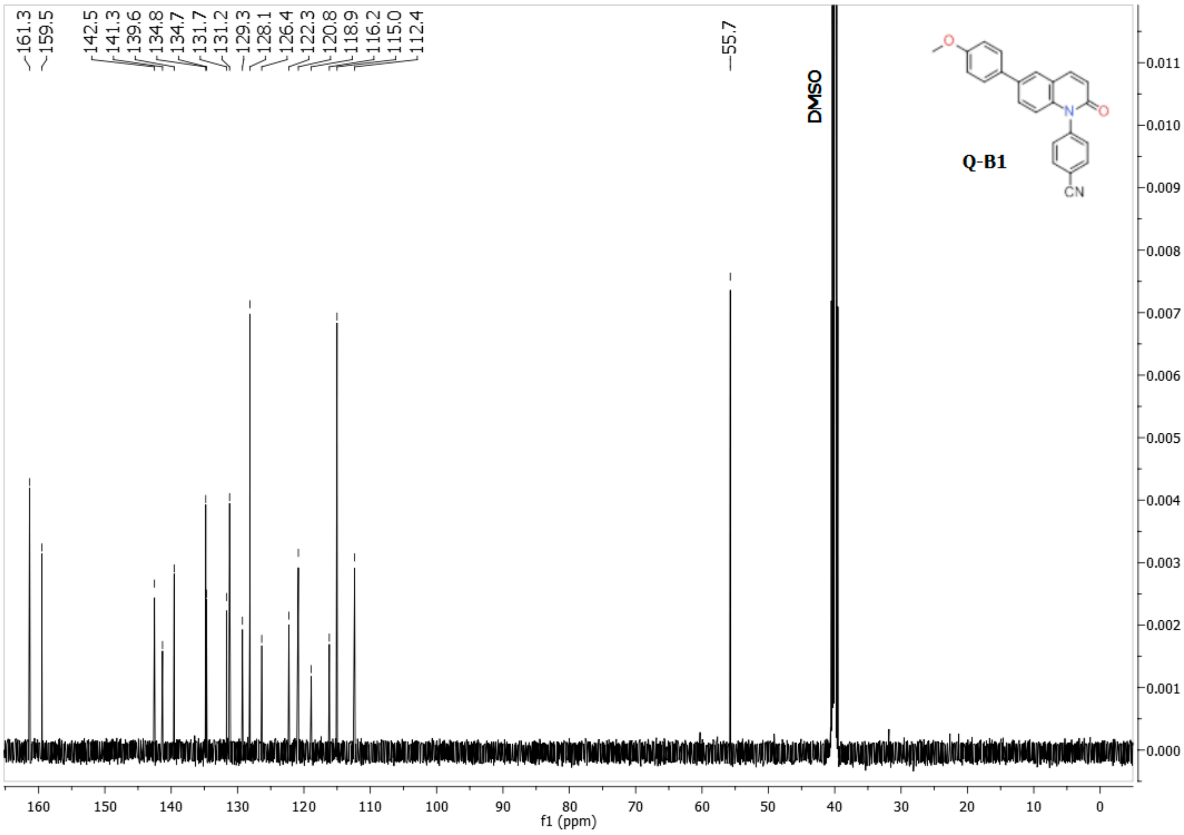

Figure S14. ^{13}CNMR of 1-(4-cyanophenyl)-6-(4-methoxyphenyl)quinolin-2-one (Q-B1).

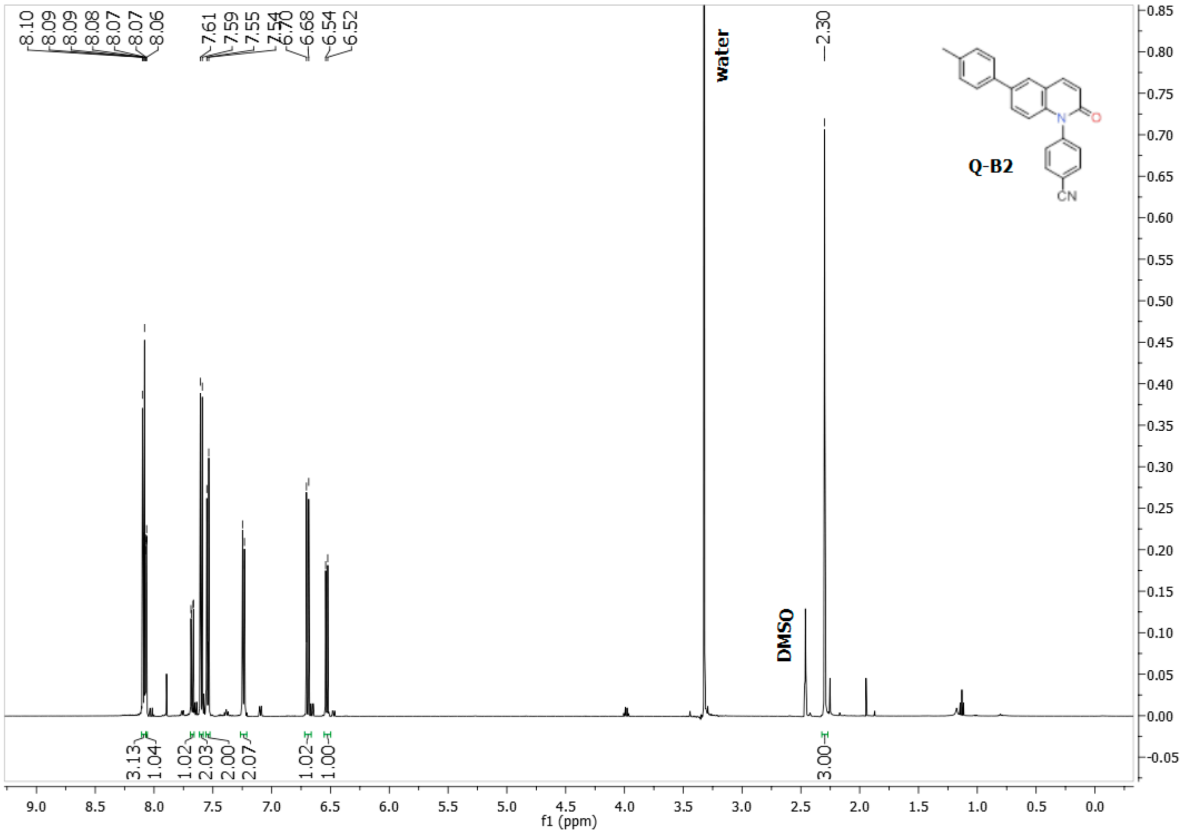

Figure S15. ^{1}HNMR of 1-(4-cyanophenyl)-6-(4-methylphenyl)quinolin-2-one (Q-B2).

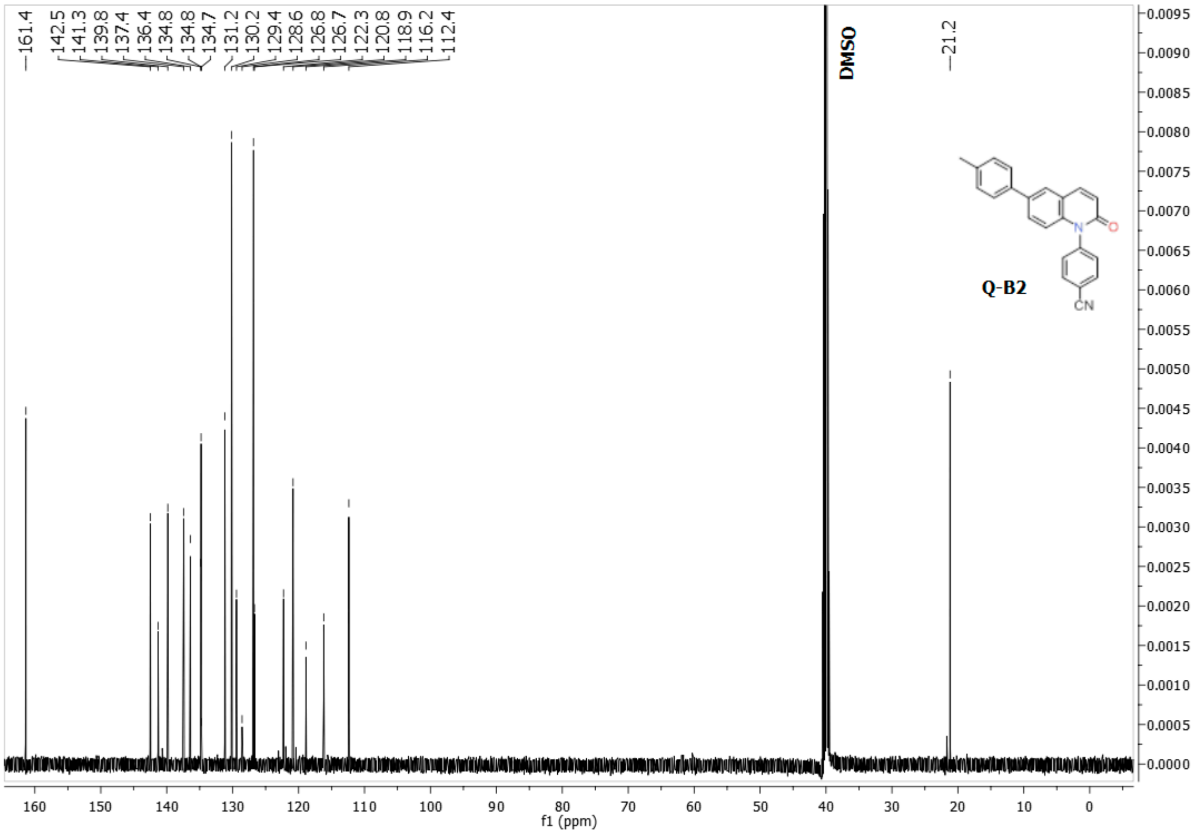

Figure S16. ^{13}CNMR of 1-(4-cyanophenyl)-6-(4-methylphenyl)quinolin-2-one (Q-B2).

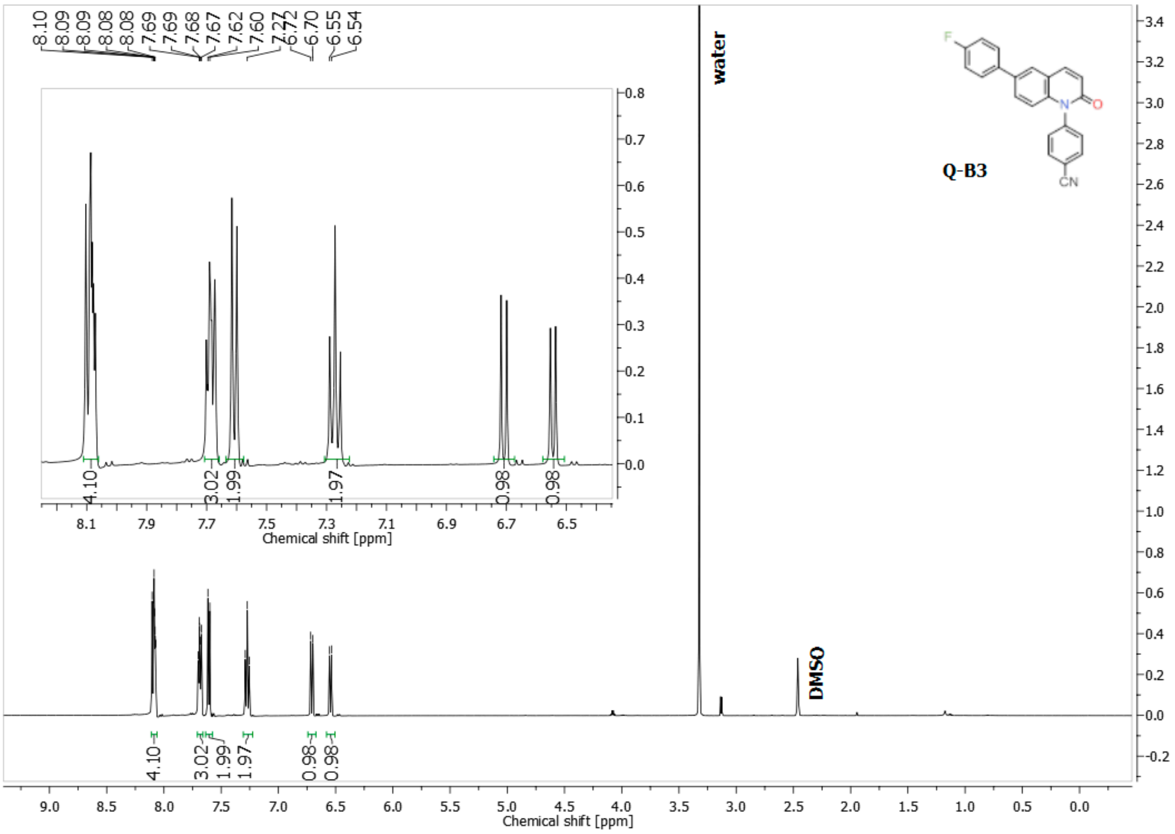

Figure S17. ^{1}HNMR of 1-(4-cyanophenyl)-6-(4-fluorophenyl)quinolin-2-one (Q-B3).

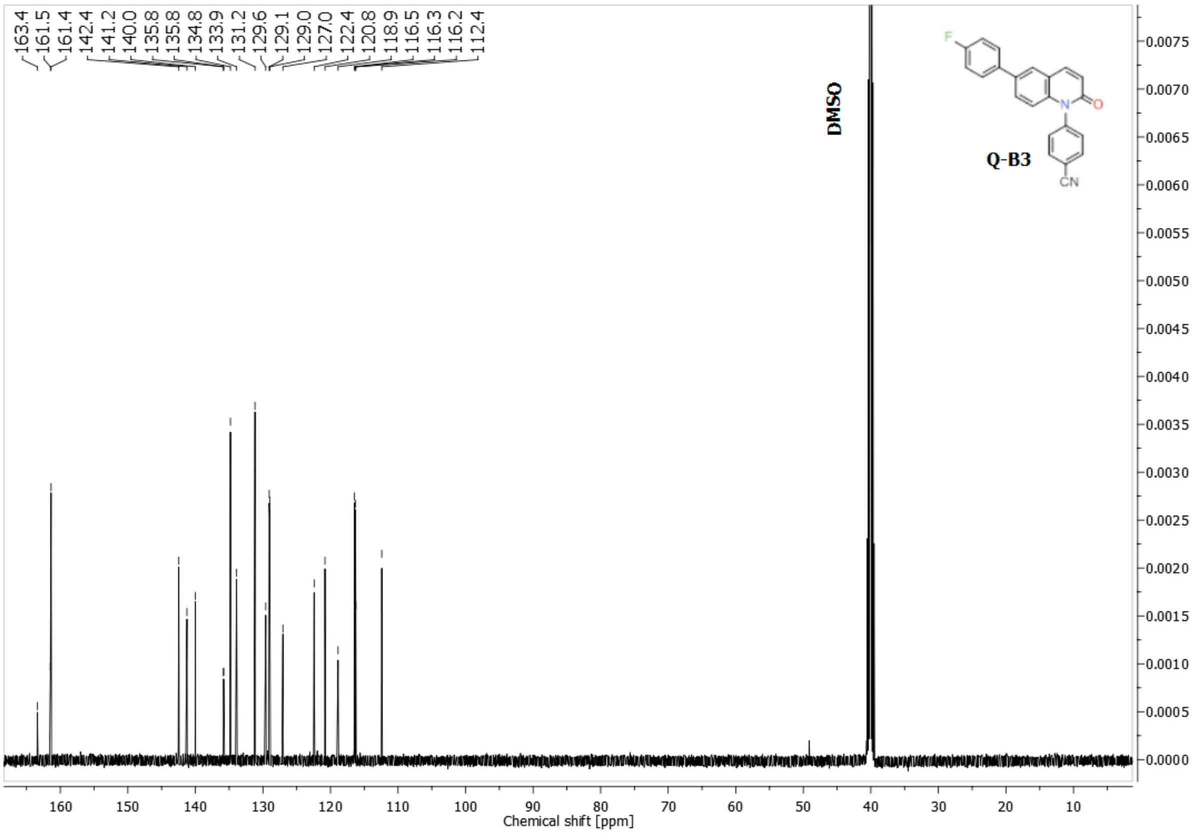

Figure S18. ^{13}CNMR of 1-(4-cyanophenyl)-6-(4-fluorophenyl)quinolin-2-one (Q-B3).

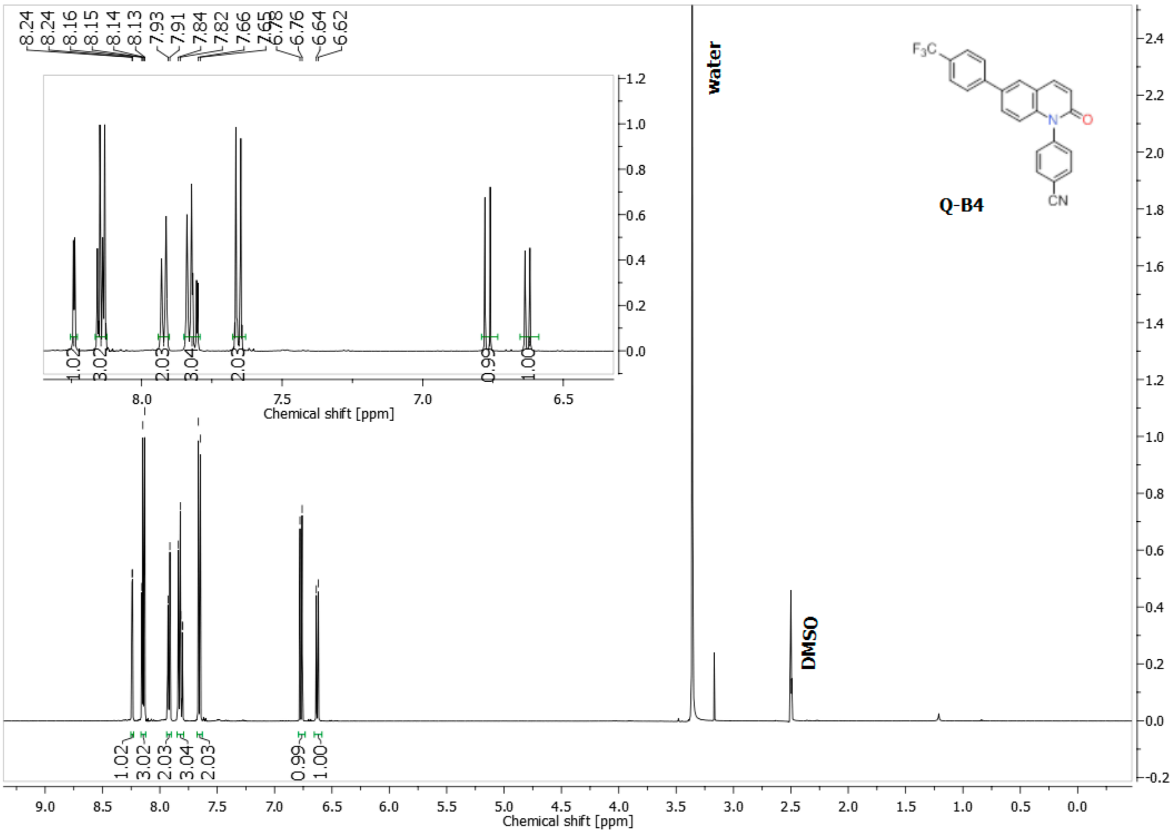

Figure S19. ^{1}HNMR of 1-(4-cyanophenyl)-6-(4-trifluoromethyl-phenyl)quinolin-2-one (Q-B4).

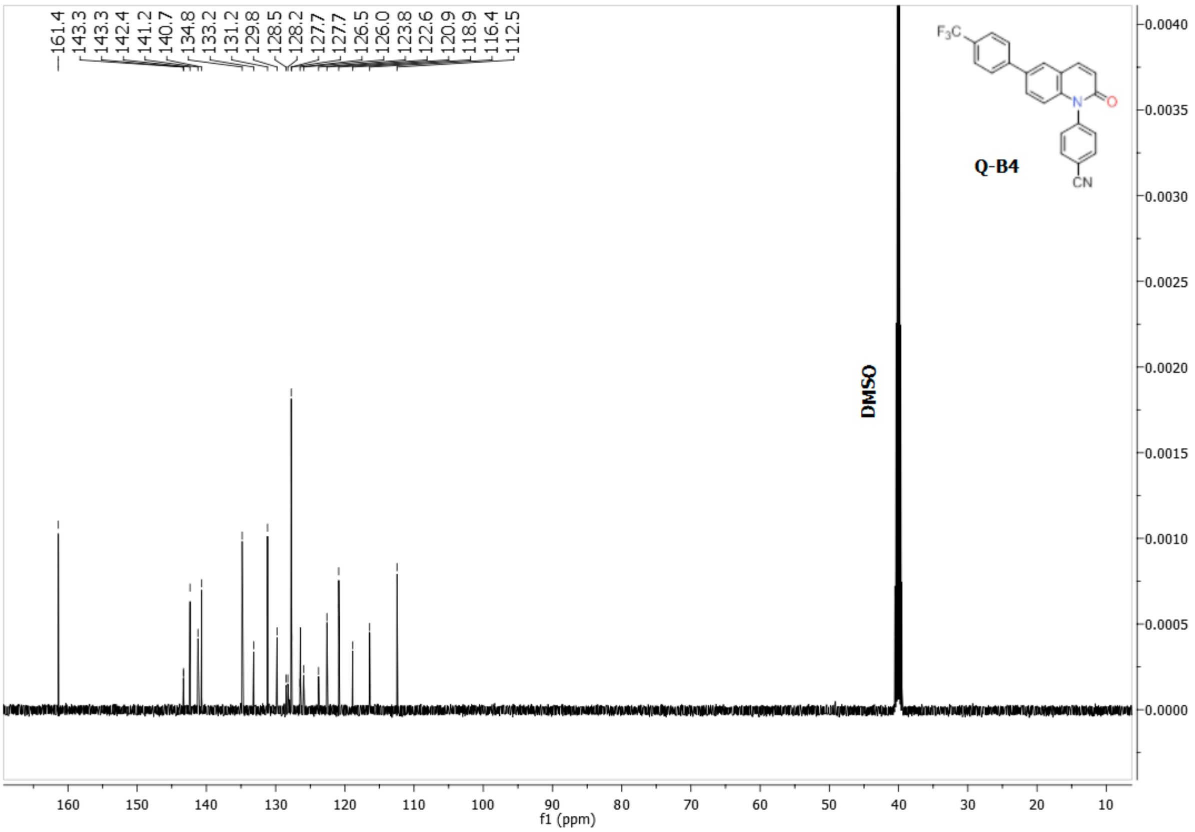

Figure S20. ^{13}CNMR of 1-(4-cyanophenyl)-6-(4-trifluoromethyl-phenyl)quinolin-2-one (Q-B4).

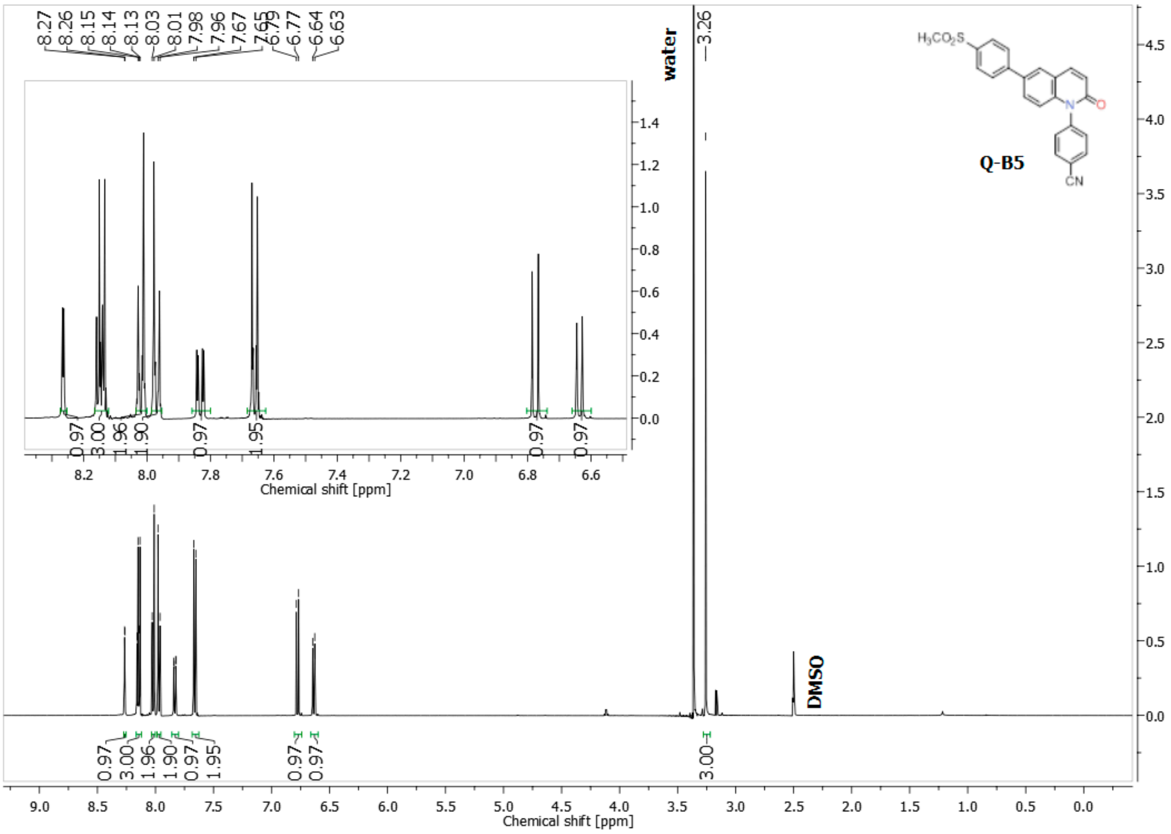

Figure S21. ^{1}HNMR of 1-(4-cyanophenyl) -6-(4-methylsulfonyl-phenyl)quinolin-2-one (Q-B5).

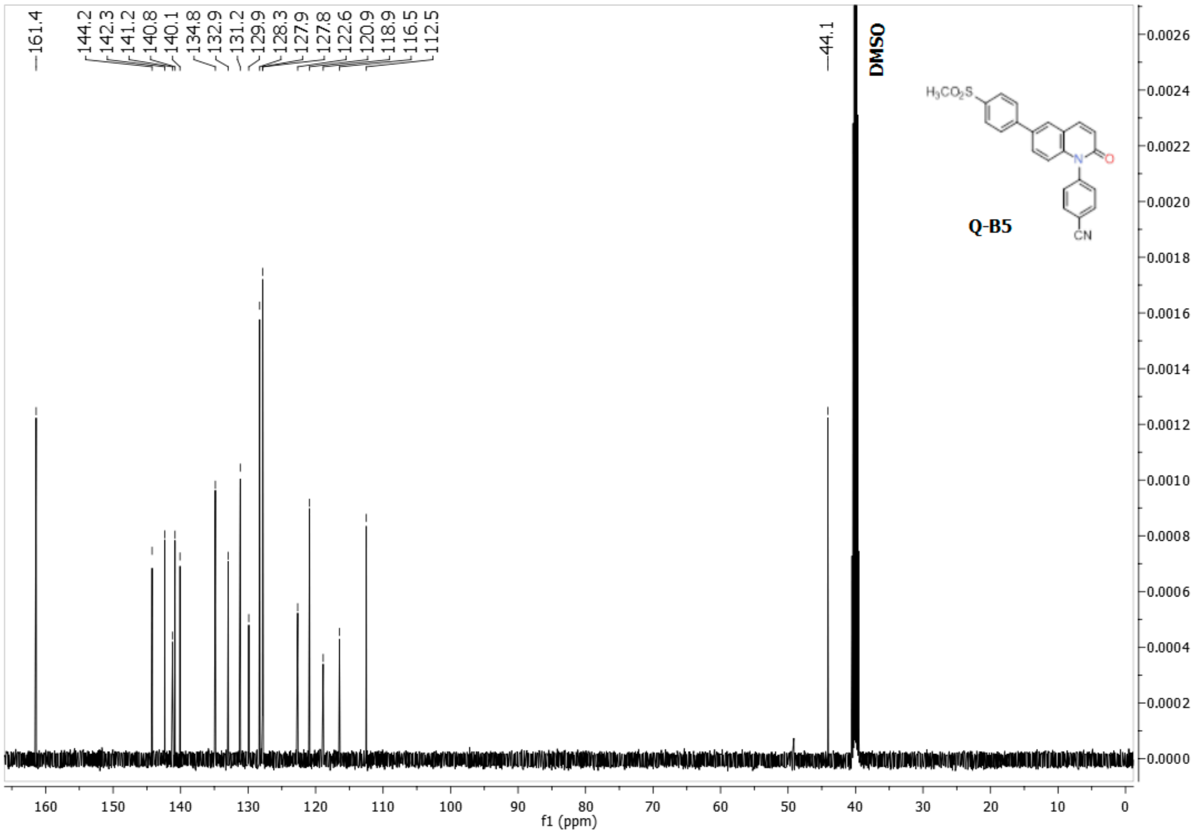

Figure S22. ^{13}CNMR of 1-(4-cyanophenyl) -6-(4-methylsulfonyl-phenyl)quinolin-2-one (Q-B5).

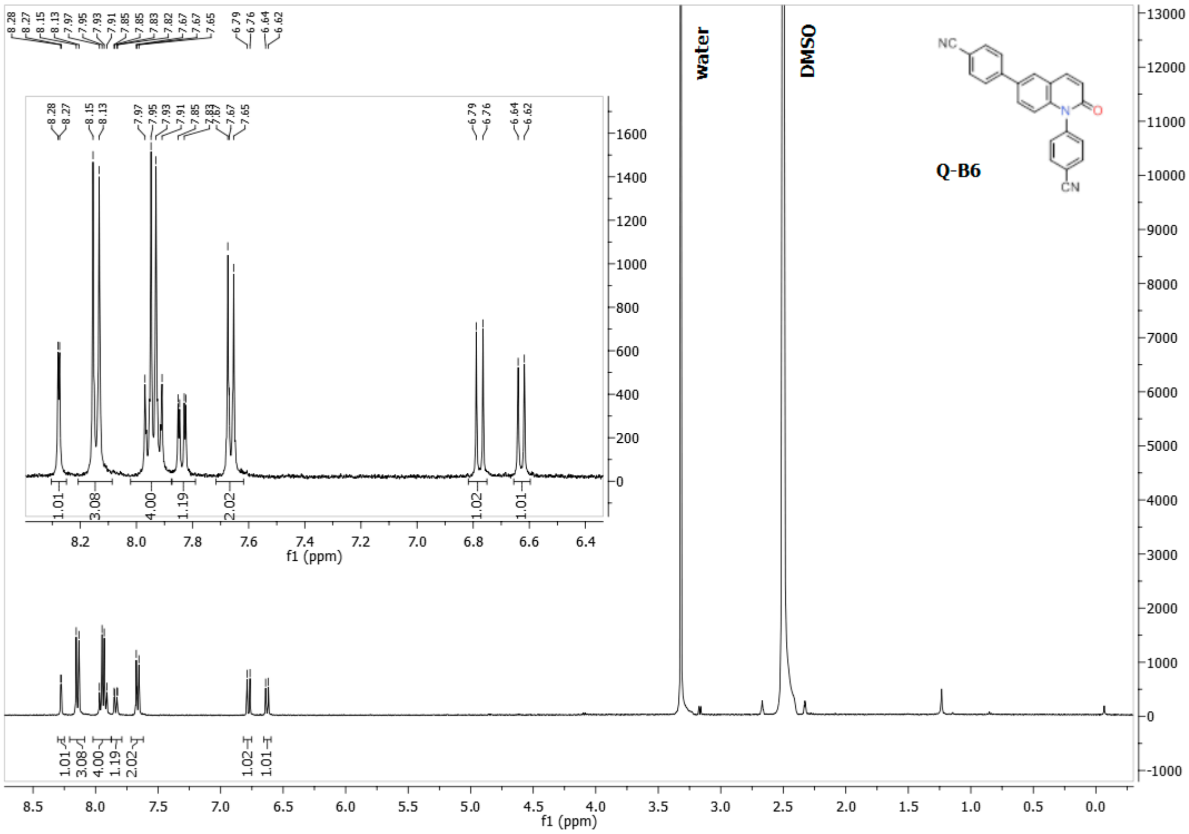

Figure S23. ^{1}HNMR of 1,6-bis (4-cyanophenyl)quinolin-2-one (Q-B6).

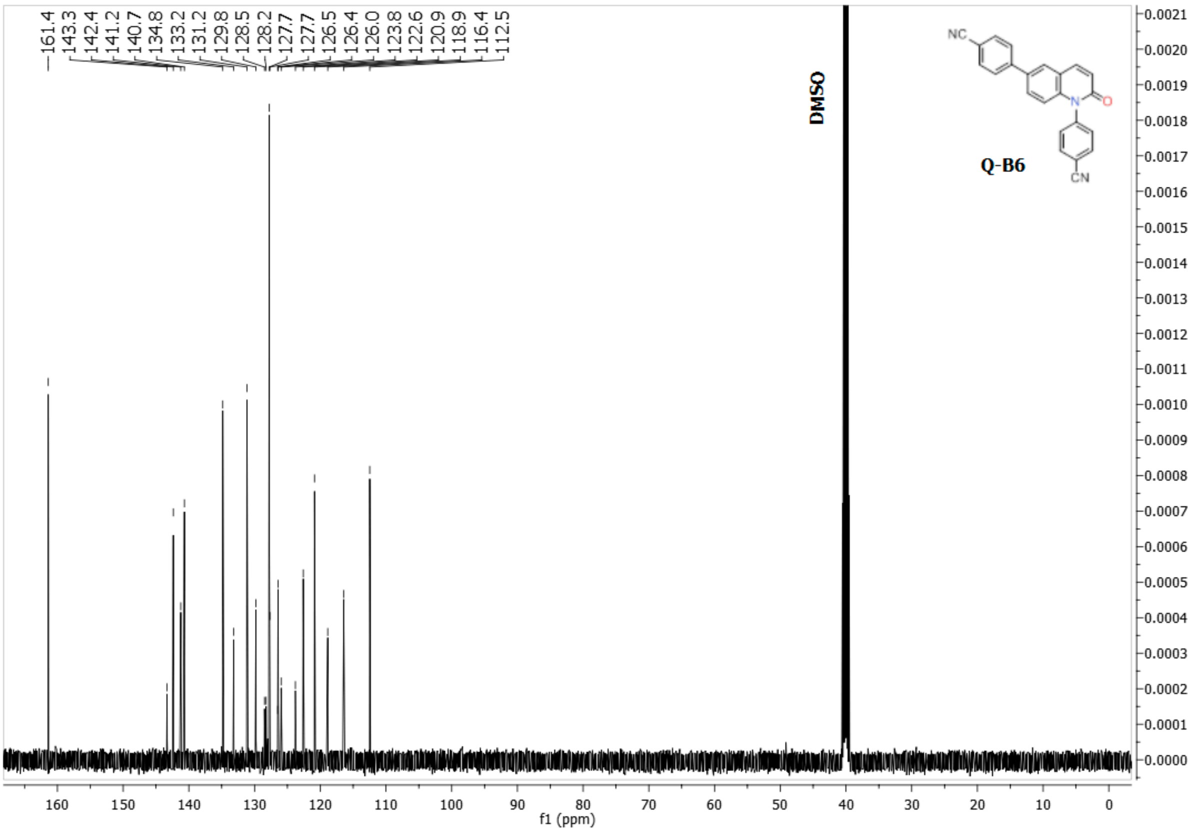

Figure S24. ^{13}CNMR of 1,6-bis (4-cyanophenyl)quinolin-2-one (Q-B6).

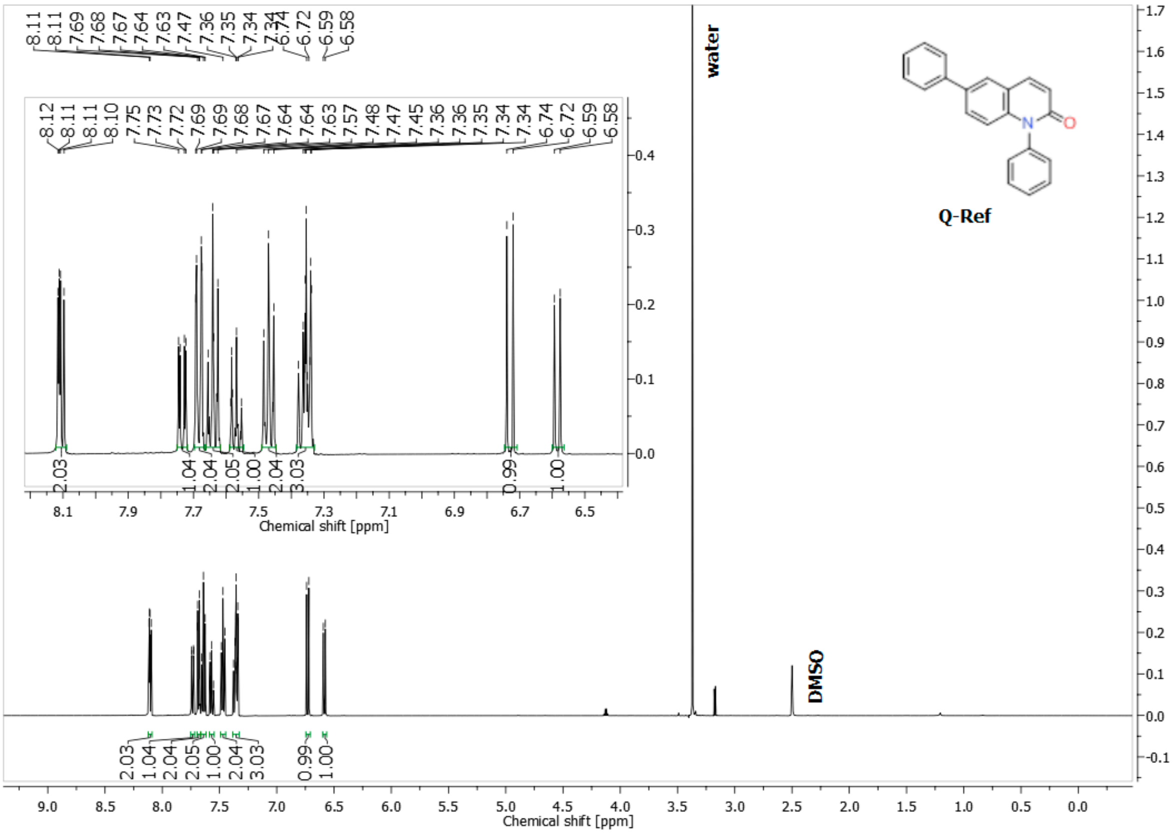

Figure S25. ^{1}HNMR of 1,6-diphenylquinolin-2-one (Q-B6).

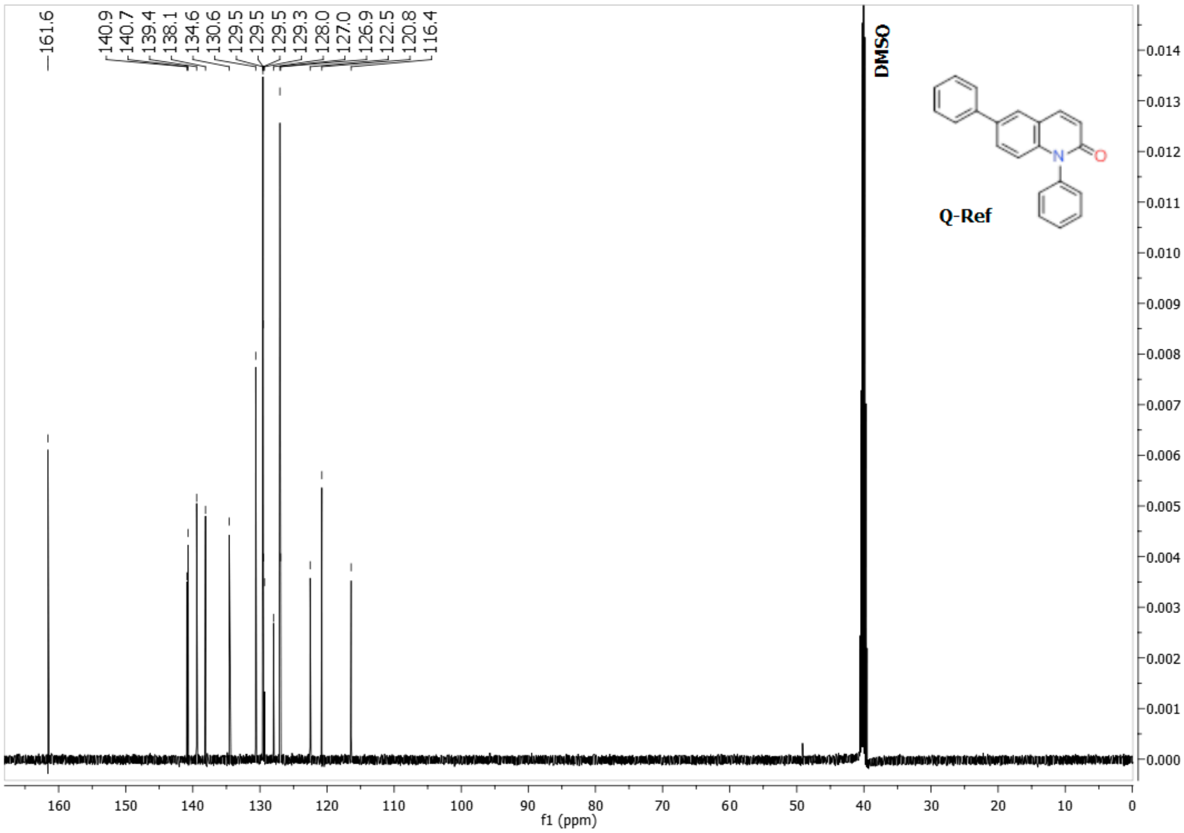

Figure S26. ^{13}CNMR of 1,6-diphenylquinolin-2-one (Q-B6).

2. Spectroscopic Properties of the 1,6-Diphenylquinolin-2-one Derivatives

2. 1. Emission and Excitation Spectra for the Determination of the Excited Singlet State Energy for Investigated of 1,6-Diphenylquinolin-2-one Derivatives in Acetonitrile

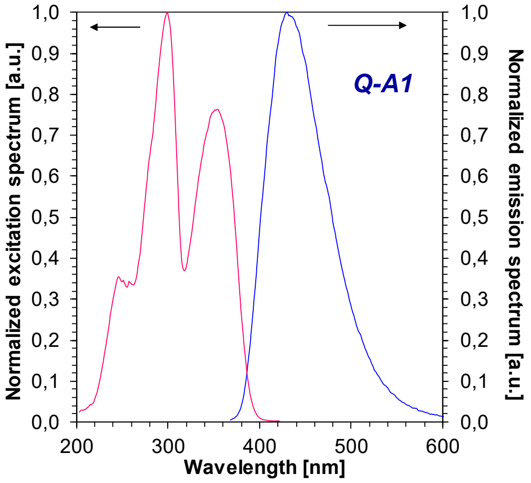

Figure S27. Emission and excitation spectra for the determination of the

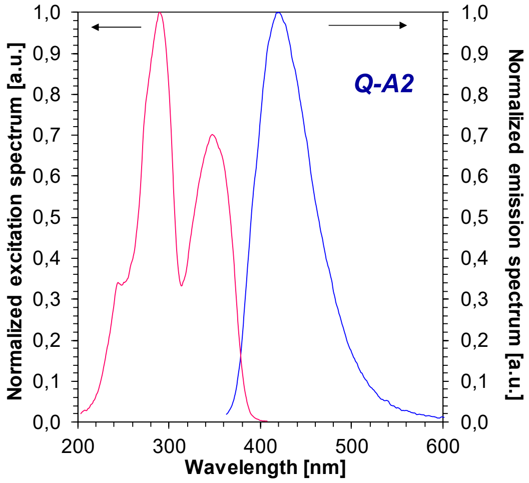

Figure S28. Emission and excitation spectra for the determination of the

excited singlet state energy for Q-A1 derivative.

excited singlet state energy for Q-A2 derivative.

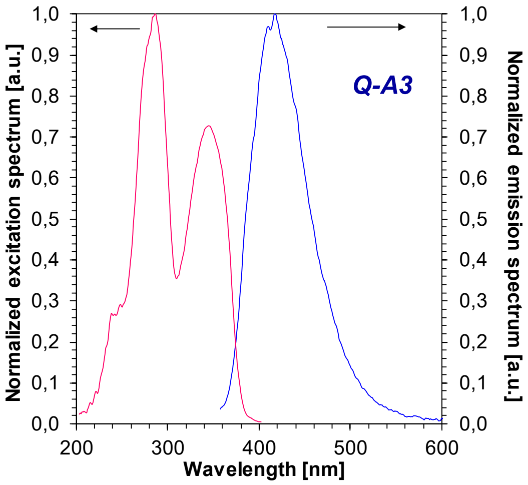

Figure S29. Emission and excitation spectra for the determination of the excited singlet state energy for Q-A3 derivative.

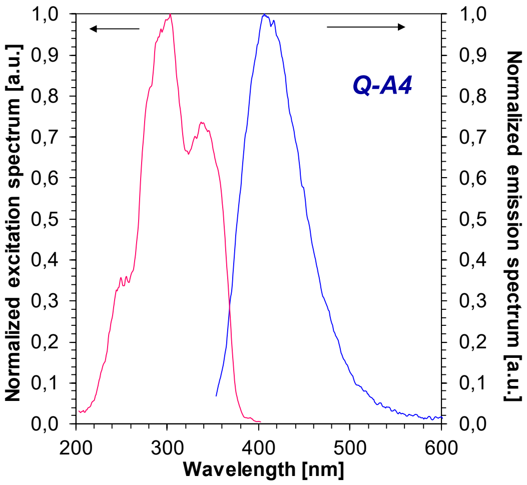

Figure S30. Emission and excitation spectra for the determination of the excited singlet state energy for Q-A4 derivative.

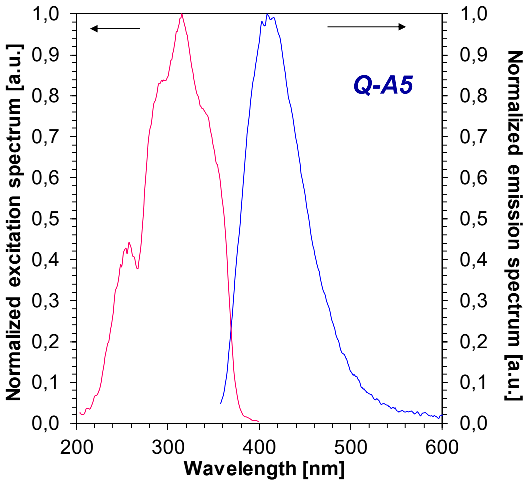

Figure S31. Emission and excitation spectra for the determination of the excited singlet state energy for Q-A5 derivative.

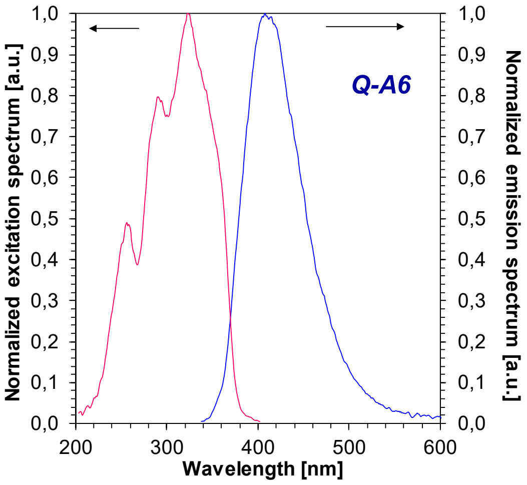

Figure S32. Emission and excitation spectra for the determination of the excited singlet state energy for Q-A6 derivative.

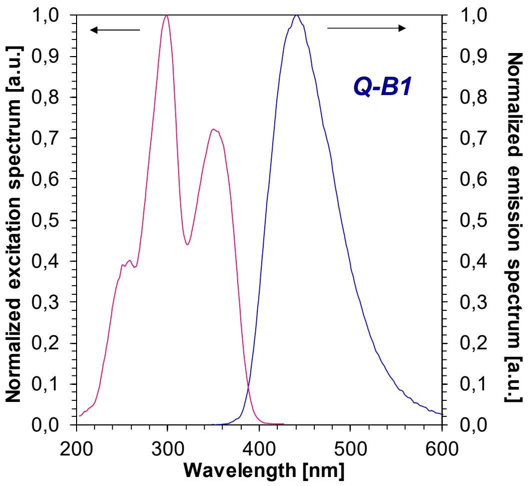

Figure S33. Emission and excitation spectra for the determination of the excited singlet state energy for Q-B1 derivative.

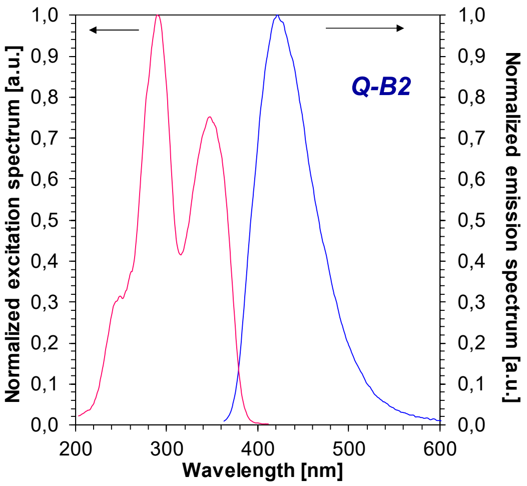

Figure S34. Emission and excitation spectra for the determination of the excited singlet state energy for Q-B2 derivative.

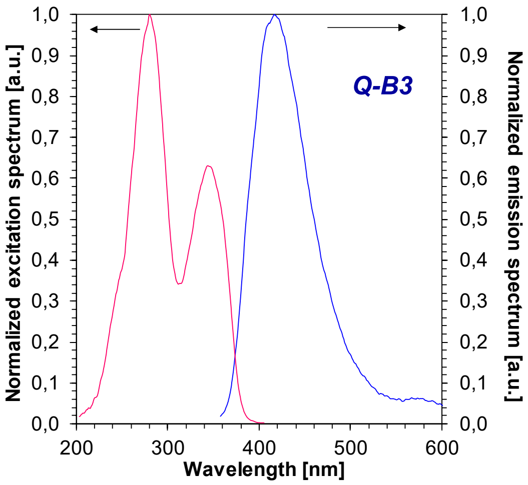

Figure S35. Emission and excitation spectra for the determination of the excited singlet state energy for Q-B3 derivative.

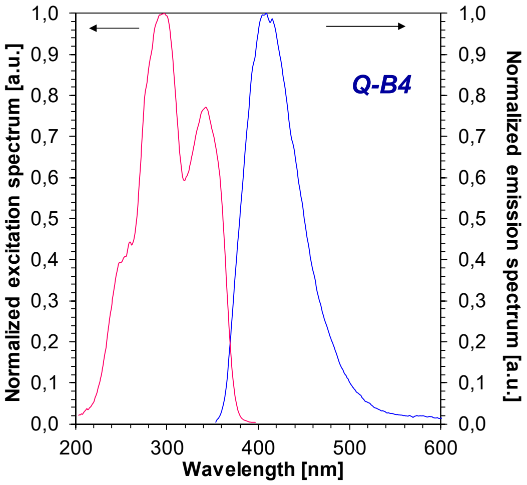

Figure S36. Emission and excitation spectra for the determination of the excited singlet state energy for Q-B4 derivative.

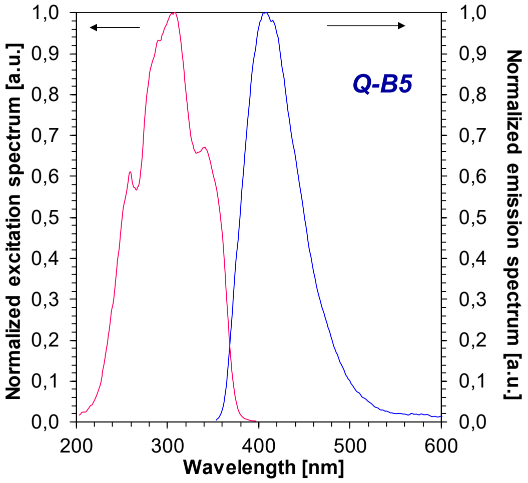

Figure S37. Emission and excitation spectra for the determination of the excited singlet state energy for Q-B5 derivative.

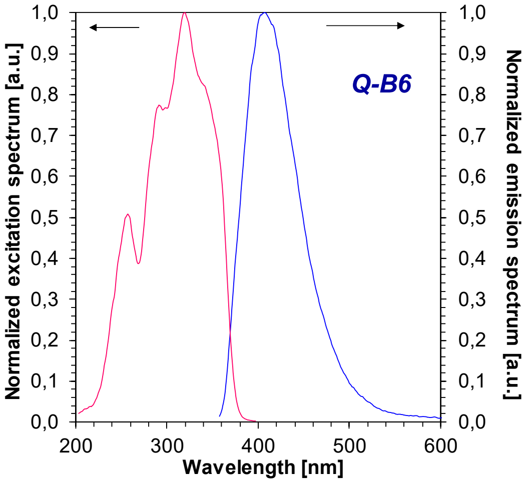

Figure S38. Emission and excitation spectra for the determination of the excited singlet state energy for Q-B6 derivative.

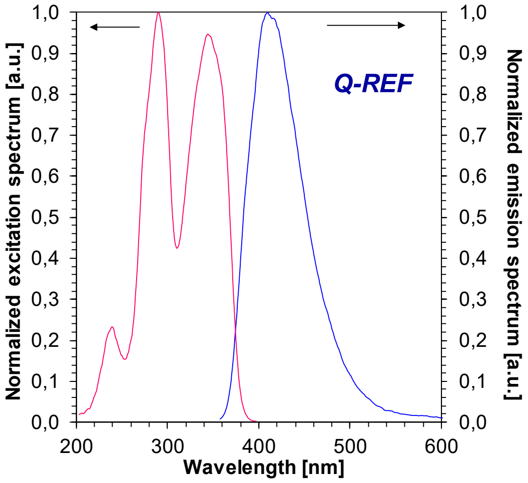

Figure S39. Emission and excitation spectra for the determination of the excited singlet state energy for Q-REF derivative.

3. Applicability of 1,6-Diphenylquinolin-2-one Derivatives for On-line Monitoring Progress of Photopolymerization Processes by FPT Method

3. 1. Applicability of 1,6-Diphenylquinolin-2-one Derivatives for On-line Monitoring Progress of Free-radical Photopolymerization Processes.

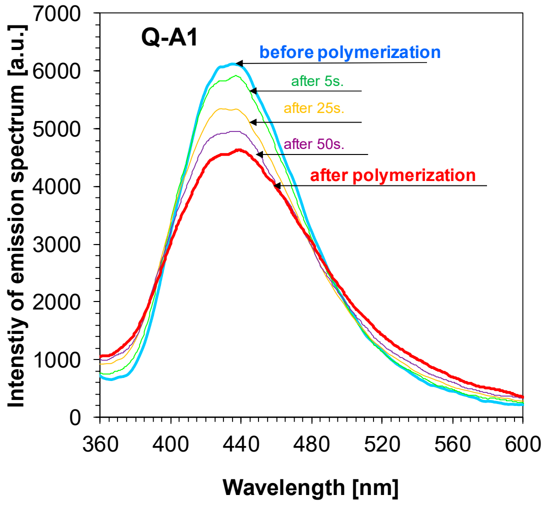

Figure S40. Changes of fluorescence spectra for probe Q-A1 during free radical photopolymerization of TMPTA monomer under irradiation 320 nm.

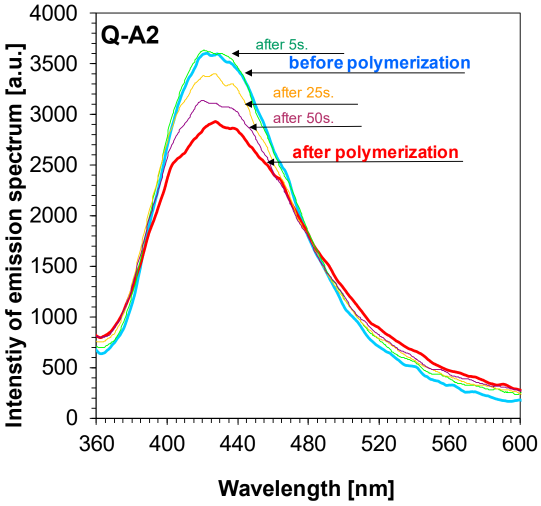

Figure S41. Changes of fluorescence spectra for probe Q-A2 during free radical photopolymerization of TMPTA monomer under irradiation 320 nm.

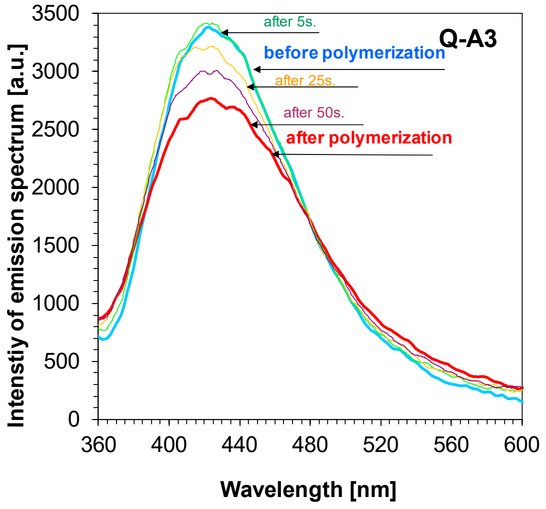

Figure S42. Changes of fluorescence spectra for probe Q-A3 during free radical photopolymerization of TMPTA monomer under irradiation 320 nm.

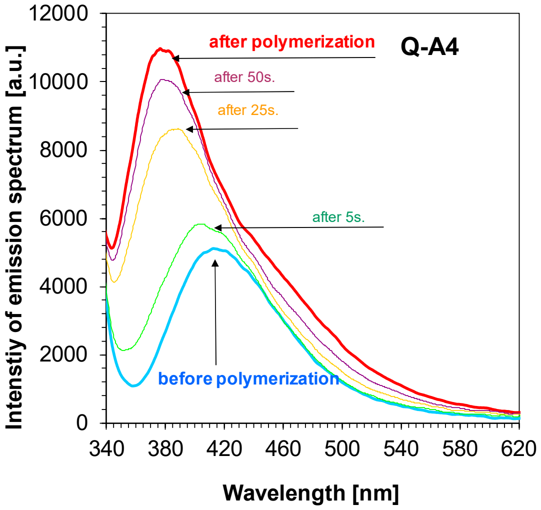

Figure S43. Changes of fluorescence spectra for probe Q-A4 during free radical photopolymerization of TMPTA monomer under irradiation 320 nm.

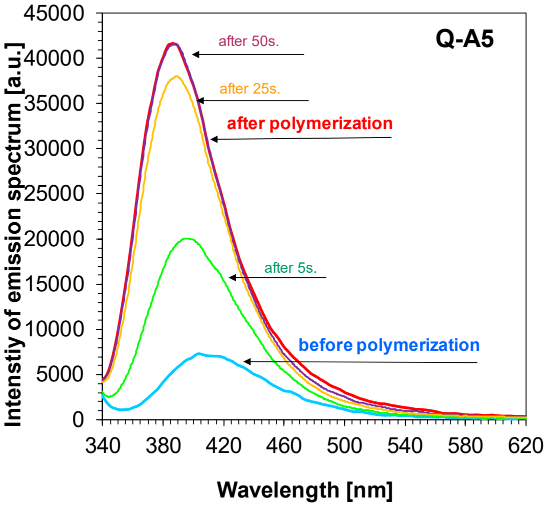

Figure S44. Changes of fluorescence spectra for probe Q-A5 during free radical photopolymerization of TMPTA monomer under irradiation 320 nm.

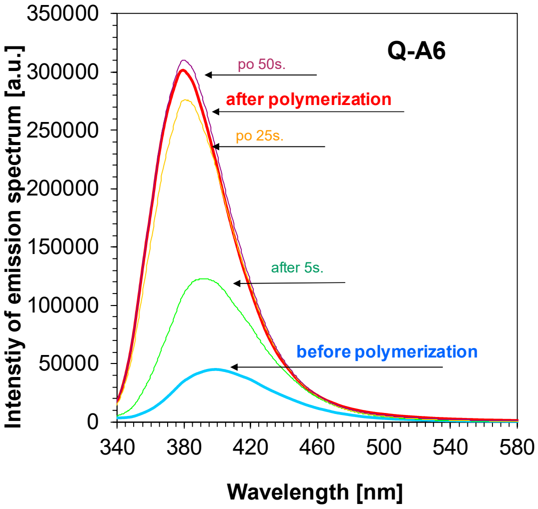

Figure S45. Changes of fluorescence spectra for probe Q-A6 during free radical photopolymerization of TMPTA monomer under irradiation 320 nm.

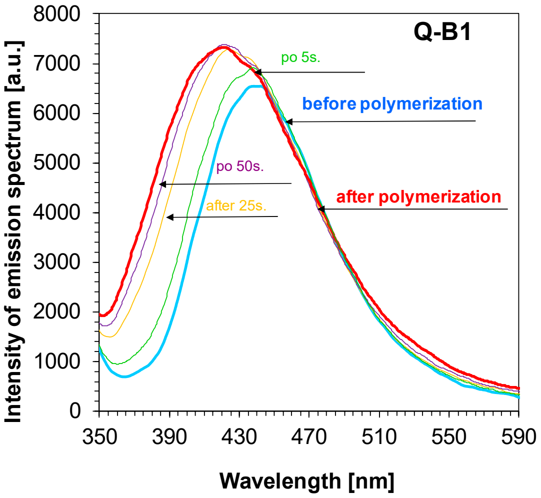

Figure S46. Changes of fluorescence spectra for probe Q-B1 during free radical photopolymerization of TMPTA monomer under irradiation 320 nm.

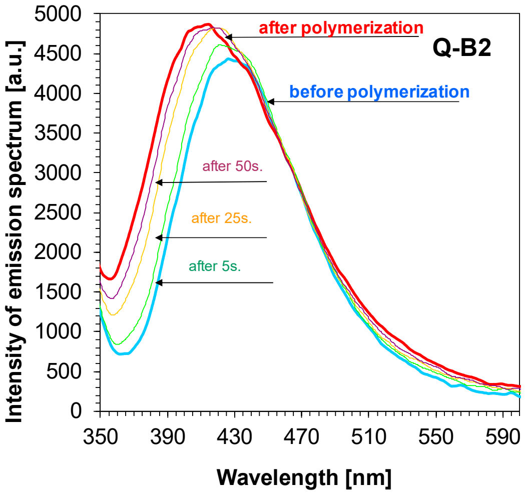

Figure S47. Changes of fluorescence spectra for probe Q-B2 during free radical photopolymerization of TMPTA monomer under irradiation 320 nm.

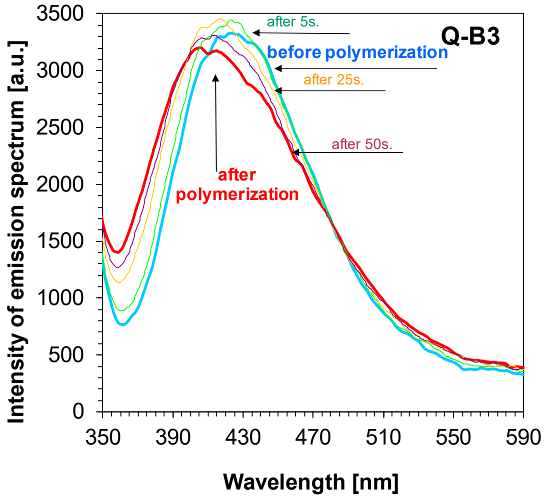

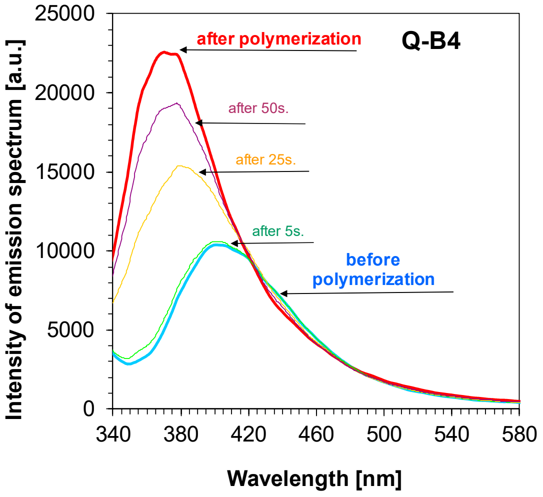

Figure S48. Changes of fluorescence spectra for probe Q-B3 during free radical photopolymerization of TMPTA monomer under irradiation 320 nm.

Figure S49. Changes of fluorescence spectra for probe Q-B4 during free radical photopolymerization of TMPTA monomer under irradiation 320 nm.

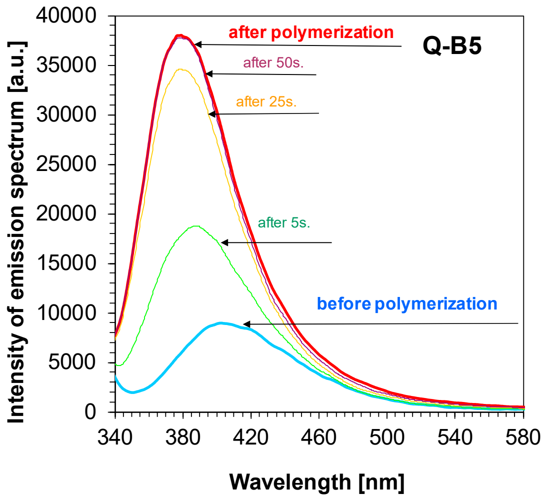

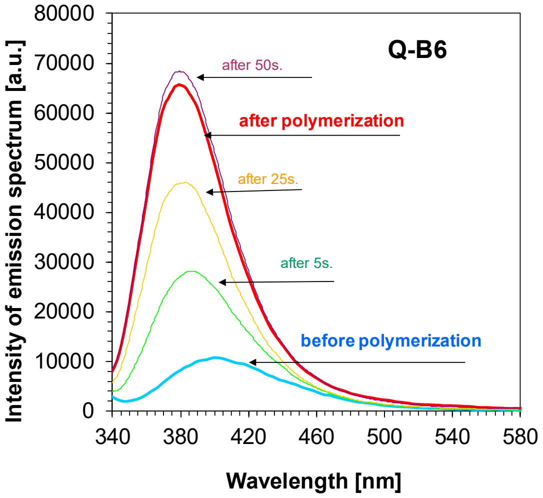

Figure S50. Changes of fluorescence spectra for probe Q-B5 during free radical photopolymerization of TMPTA monomer under irradiation 320 nm.

Figure S51. Changes of fluorescence spectra for probe Q-B6 during free radical photopolymerization of TMPTA monomer under irradiation 320 nm.

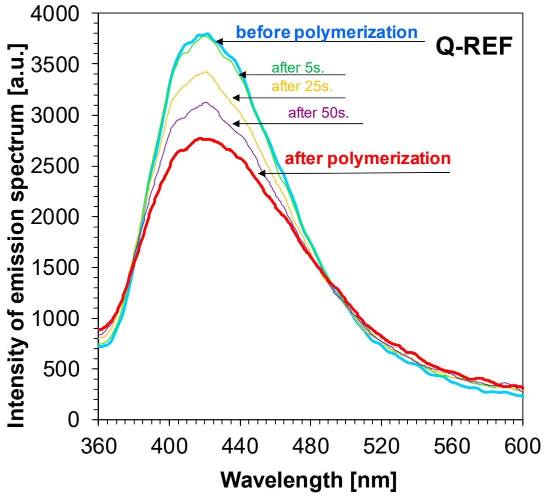

Figure S52. Changes of fluorescence spectra for probe Q-REF during free radical photopolymerization of TMPTA monomer under irradiation 320 nm.

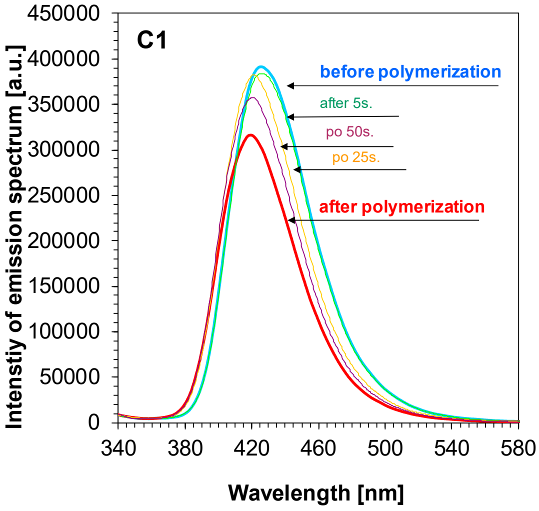

Figure S53. Changes of fluorescence spectra for probe C1 during free radical photopolymerization of TMPTA monomer under irradiation 320 nm.

3. 2. Applicability of 1,6-Diphenylquinolin-2-one Derivatives for On-line Monitoring Progress of Thiol-ene Photopolymerization Processes.

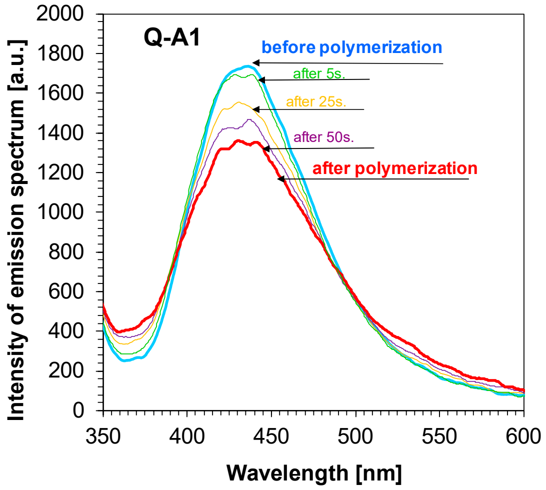

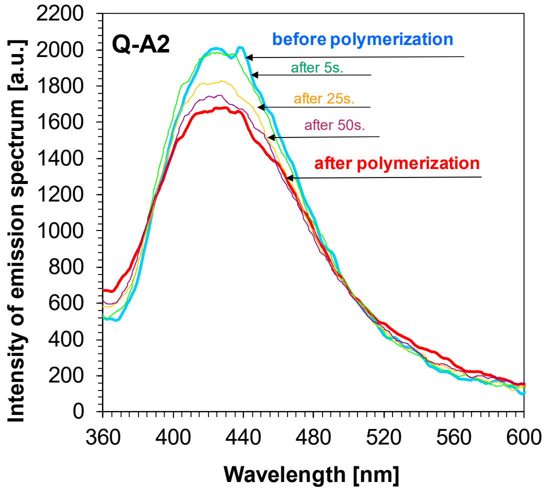

Figure S54. Changes of fluorescence spectra for probe Q-A1 during thiol-en photopolymerization of TMPTA/MERCAPTO (0,5/0,5% w/w) monomers under irradiation 320 nm.

Figure S55. Changes of fluorescence spectra for probe Q-A2 during thiol-en photopolymerization of TMPTA/MERCAPTO (0,5/0,5% w/w) monomers under irradiation 320 nm.

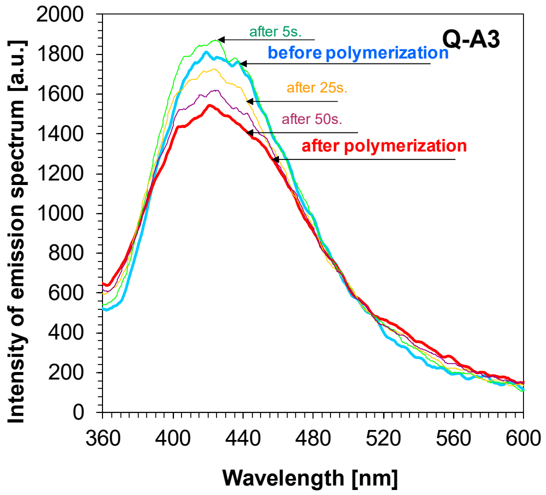

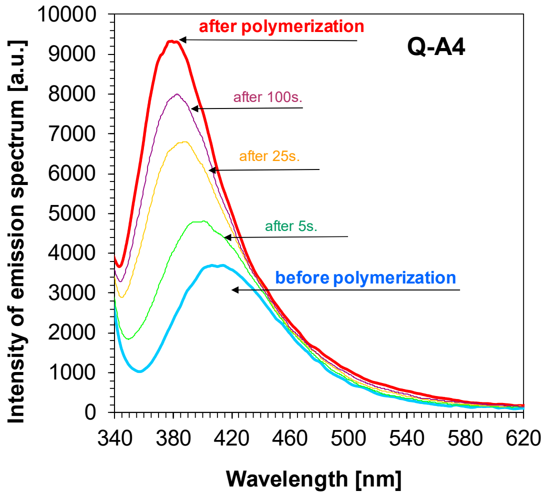

Figure S56. Changes of fluorescence spectra for probe Q-A3 during thiol-en photopolymerization of TMPTA/MERCAPTO (0,5/0,5% w/w) monomers under irradiation 320 nm.

Figure S57. Changes of fluorescence spectra for probe Q-A4 during thiol-en photopolymerization of TMPTA/MERCAPTO (0,5/0,5% w/w) monomers under irradiation 320 nm.

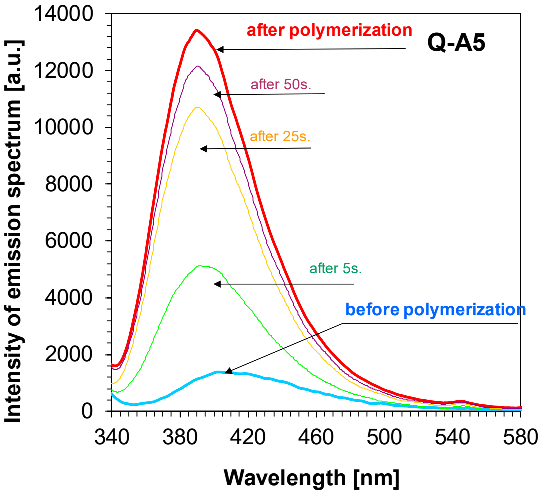

Figure S58. Changes of fluorescence spectra for probe Q-A5 during thiol-en photopolymerization of TMPTA/MERCAPTO (0,5/0,5% w/w) monomers under irradiation 320 nm.

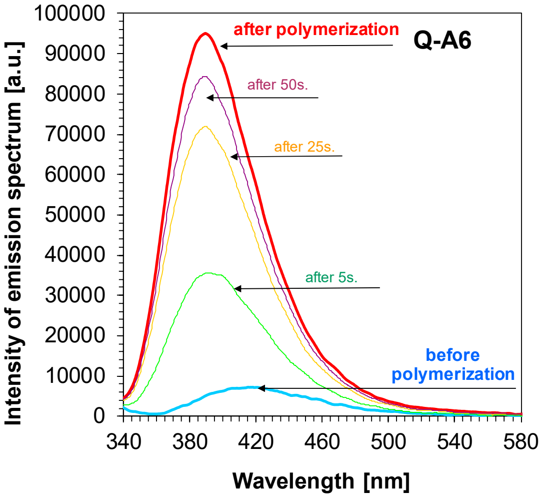

Figure S59. Changes of fluorescence spectra for probe Q-A6 during thiol-en photopolymerization of TMPTA/MERCAPTO (0,5/0,5% w/w) monomers under irradiation 320 nm.

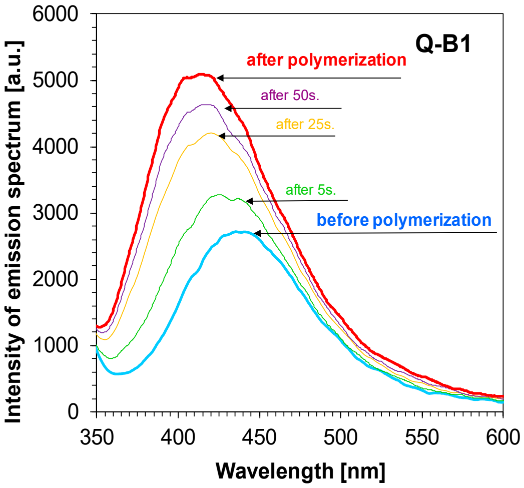

Figure S60. Changes of fluorescence spectra for probe Q-B1 during thiol-en photopolymerization of TMPTA/MERCAPTO (0,5/0,5% w/w) monomers under irradiation 320 nm.

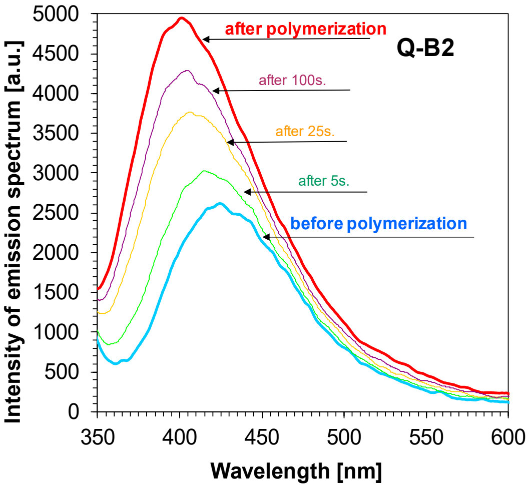

Figure S61. Changes of fluorescence spectra for probe Q-B2 during thiol-en photopolymerization of TMPTA/MERCAPTO (0,5/0,5% w/w) monomers under irradiation 320 nm.

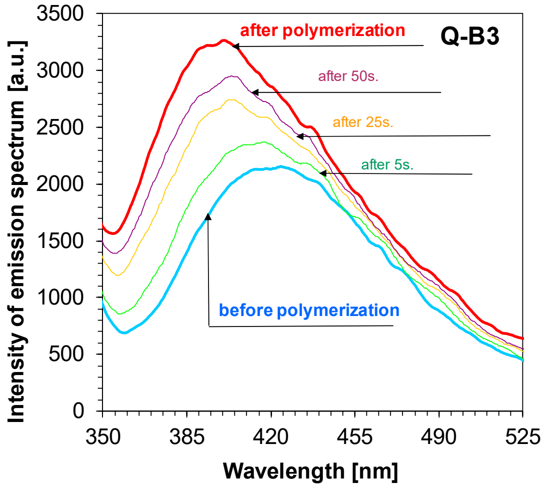

Figure S62. Changes of fluorescence spectra for probe Q-B3 during thiol-en photopolymerization of TMPTA/MERCAPTO (0,5/0,5% w/w) monomers under irradiation 320 nm.

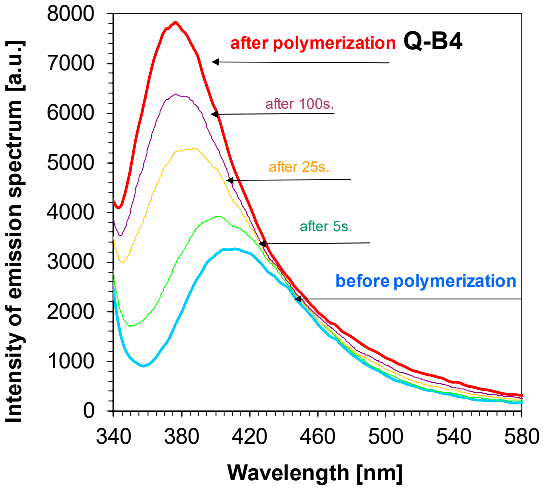

Figure S63. Changes of fluorescence spectra for probe Q-B4 during thiol-en photopolymerization of TMPTA/MERCAPTO (0,5/0,5% w/w) monomers under irradiation 320 nm.

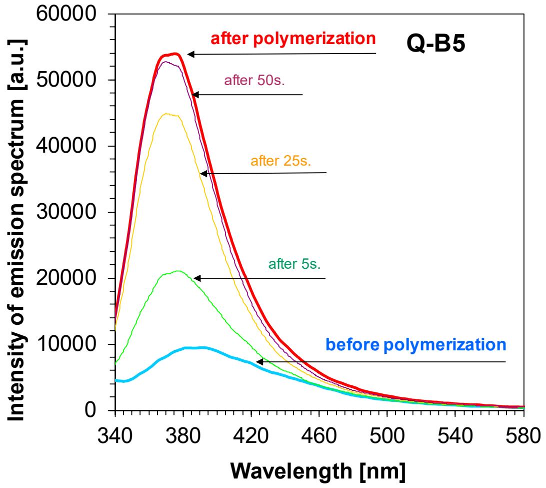

Figure S64. Changes of fluorescence spectra for probe Q-B5 during thiol-en photopolymerization of TMPTA/MERCAPTO (0,5/0,5% w/w) monomers under irradiation 320 nm.

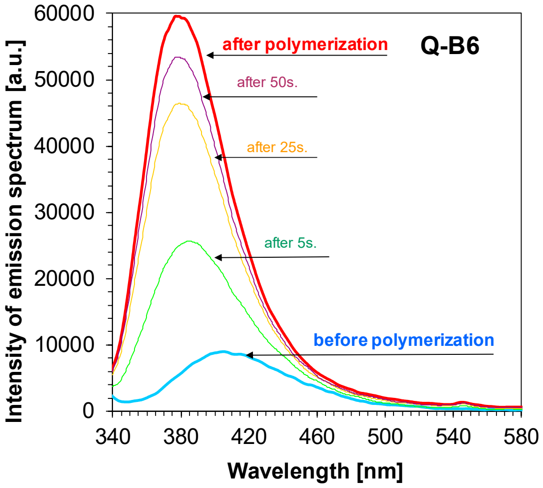

Figure S65. Changes of fluorescence spectra for probe Q-B6 during thiol-en photopolymerization of TMPTA/MERCAPTO (0,5/0,5% w/w) monomers under irradiation 320 nm.

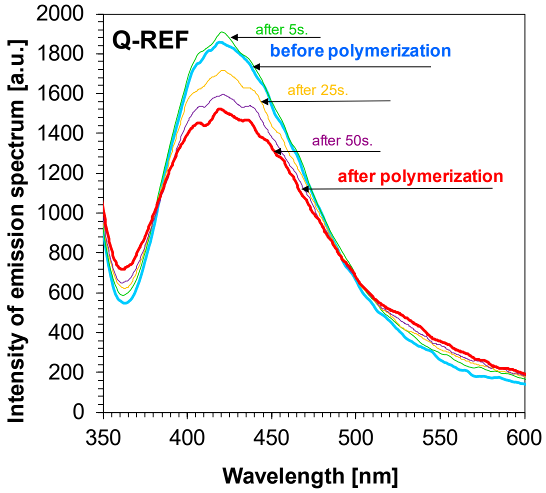

Figure S66. Changes of fluorescence spectra for probe Q-REF during thiol-en photopolymerization of TMPTA/MERCAPTO (0,5/0,5% w/w) monomers under irradiation 320 nm.

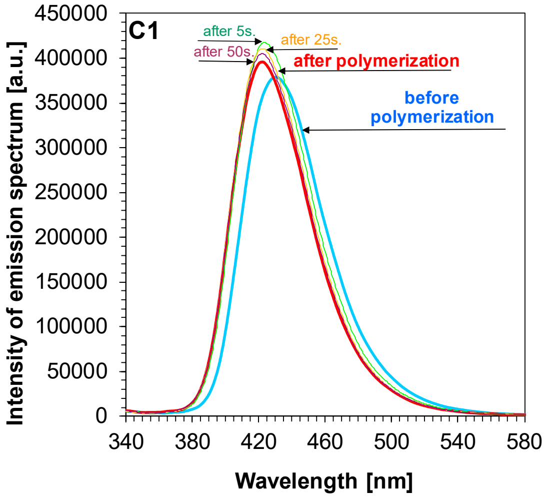

Figure S67. Changes of fluorescence spectra for probe C1 during thiol-en photopolymerization of TMPTA/MERCAPTO (0,5/0,5% w/w) monomers under irradiation 320 nm.

3. 3. Applicability of 1,6-Diphenylquinolin-2-one Derivatives for On-line Monitoring Progress of Cationic Photopolymerization Processes.

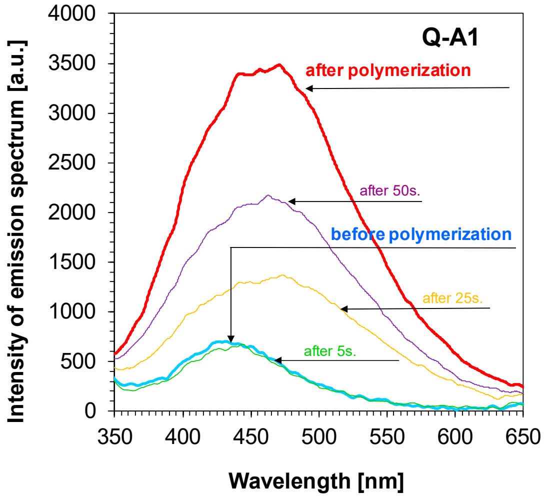

Figure S68. Changes of fluorescence spectra for probe Q-A1 during cationic photopolymerization of TEGDVE monomer under irradiation 320 nm.

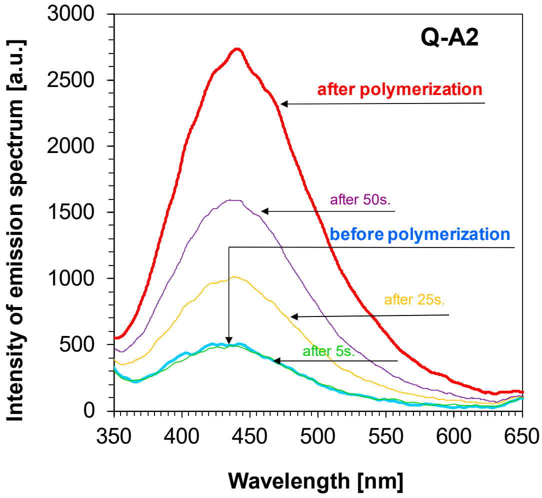

Figure S69. Changes of fluorescence spectra for probe Q-A2 during cationic photopolymerization of TEGDVE monomer under irradiation 320 nm.

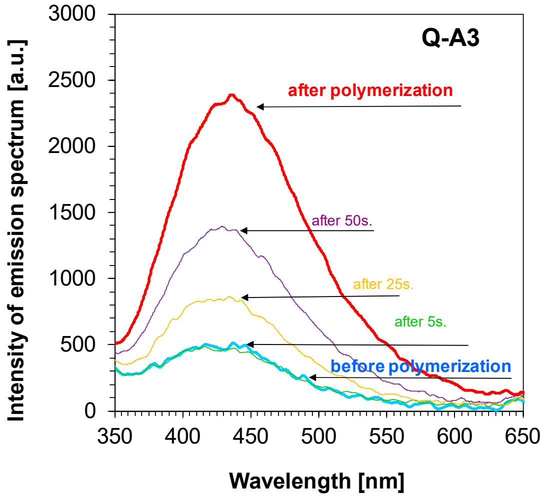

Figure S70. Changes of fluorescence spectra for probe Q-A3 during cationic photopolymerization of TEGDVE monomer under irradiation 320 nm.

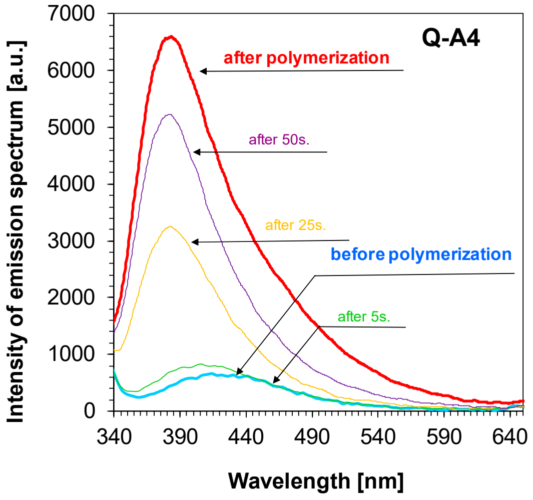

Figure S71. Changes of fluorescence spectra for probe Q-A4 during cationic photopolymerization of TEGDVE monomer under irradiation 320 nm.

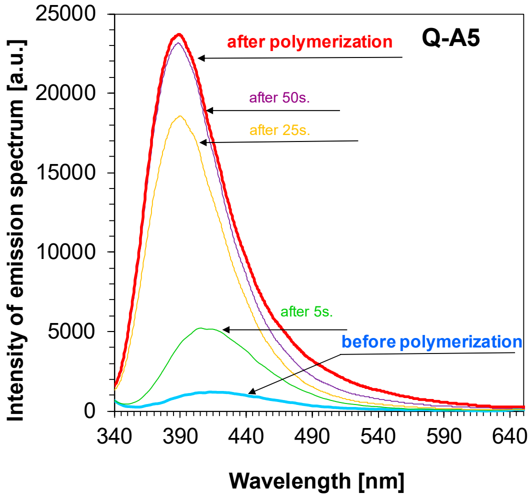

Figure S72. Changes of fluorescence spectra for probe Q-A5 during cationic photopolymerization of TEGDVE monomer under irradiation 320 nm.

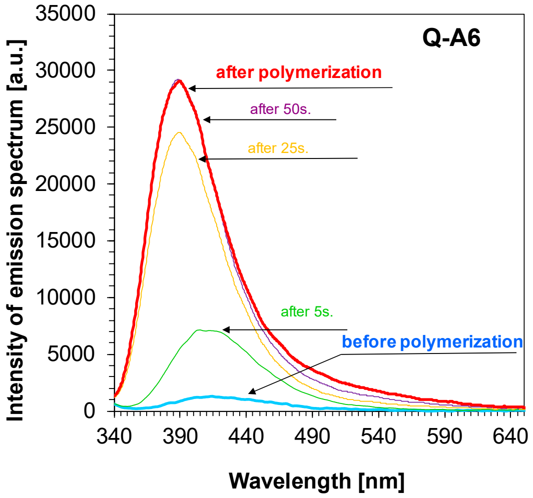

Figure S73. Changes of fluorescence spectra for probe Q-A6 during cationic photopolymerization of TEGDVE monomer under irradiation 320 nm.

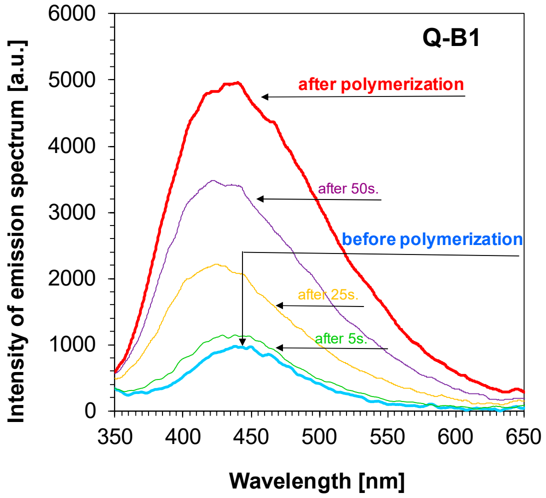

Figure S74. Changes of fluorescence spectra for probe Q-B1 during cationic photopolymerization of TEGDVE monomer under irradiation 320 nm.

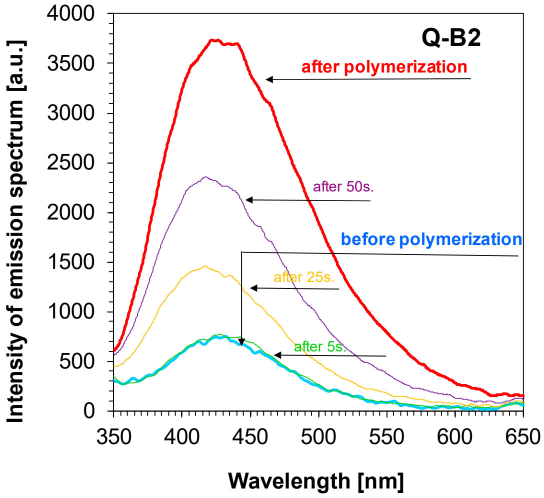

Figure S75. Changes of fluorescence spectra for probe Q-B2 during cationic photopolymerization of TEGDVE monomer under irradiation 320 nm.

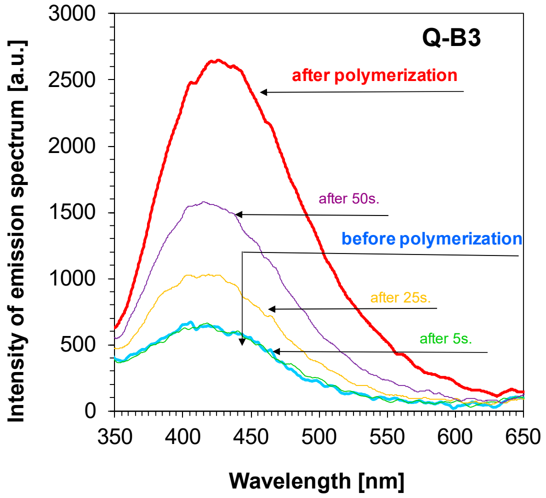

Figure S76. Changes of fluorescence spectra for probe Q-B3 during cationic photopolymerization of TEGDVE monomer under irradiation 320 nm.

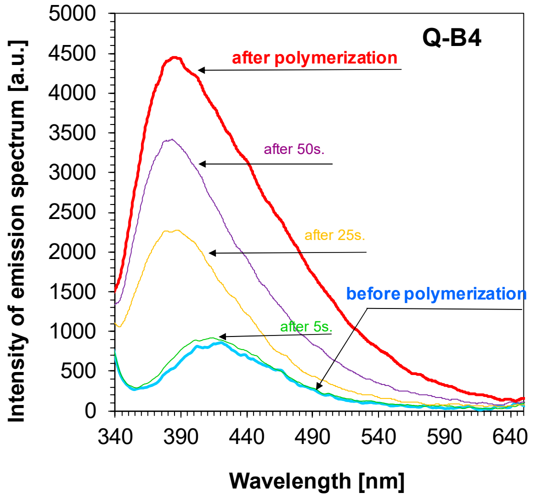

Figure S77. Changes of fluorescence spectra for probe Q-B4 during cationic photopolymerization of TEGDVE monomer under irradiation 320 nm.

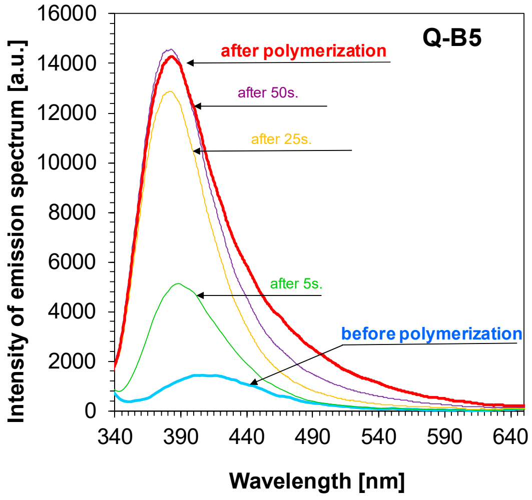

Figure S78. Changes of fluorescence spectra for probe Q-B5 during cationic photopolymerization of TEGDVE monomer under irradiation 320 nm.

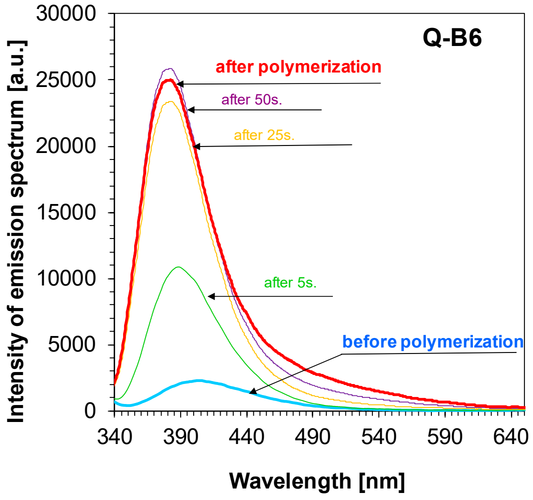

Figure S79. Changes of fluorescence spectra for probe Q-B6 during cationic photopolymerization of TEGDVE monomer under irradiation 320 nm.

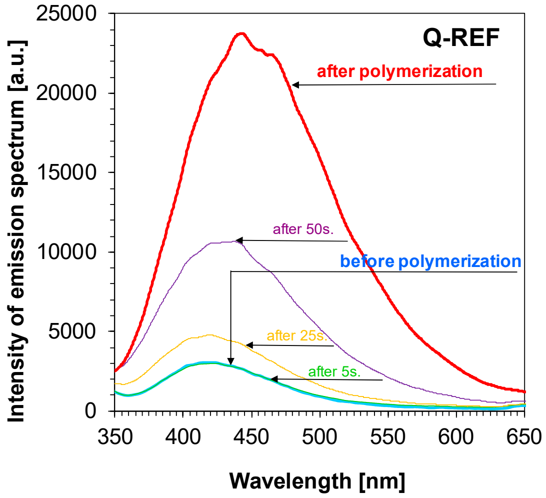

Figure S80. Changes of fluorescence spectra for probe Q-REF during cationic photopolymerization of TEGDVE monomer under irradiation 320 nm.

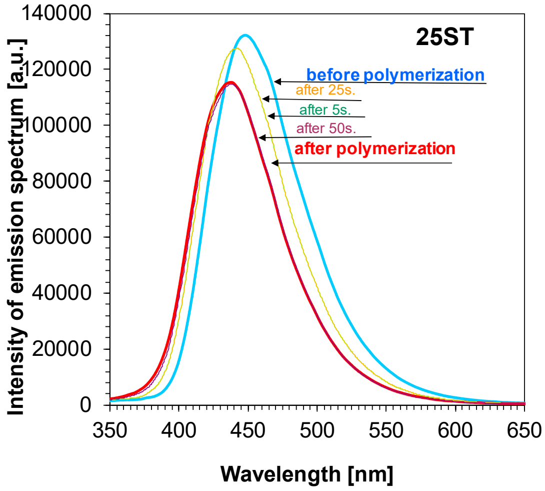

Figure S81. Changes of fluorescence spectra for probe 25ST during cationic photopolymerization of TEGDVE monomer under irradiation 320 nm.

4. Cyclic Voltammetry Curves Showing Oxidation Processes of 1,6-Diphenylquinolin-2-one Derivatives in Acetonitrile.

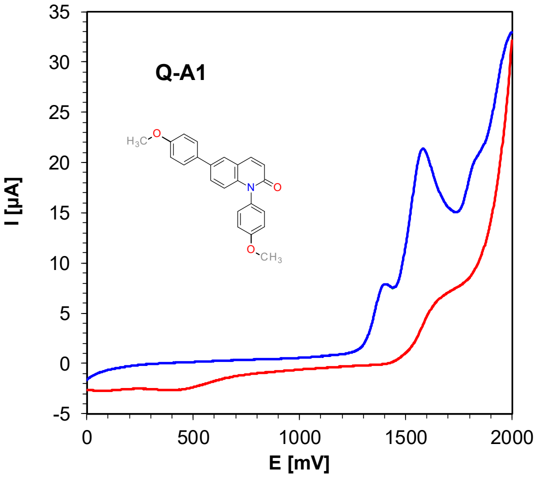

Figure S82. Cyclic voltammogram curves of the Q-A1 oxidation in acetonitrile.

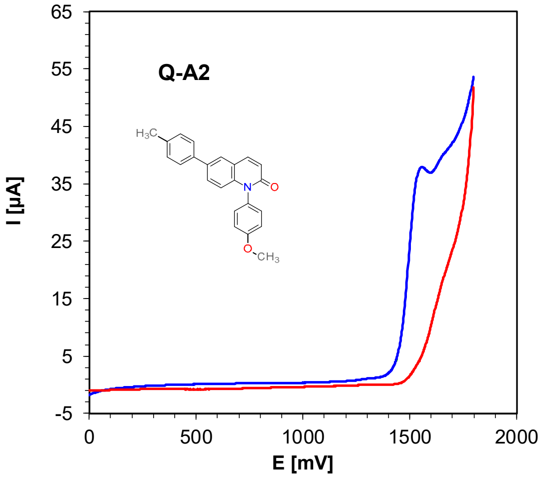

Figure S83. Cyclic voltammogram curves of the Q-A2 oxidation in acetonitrile.

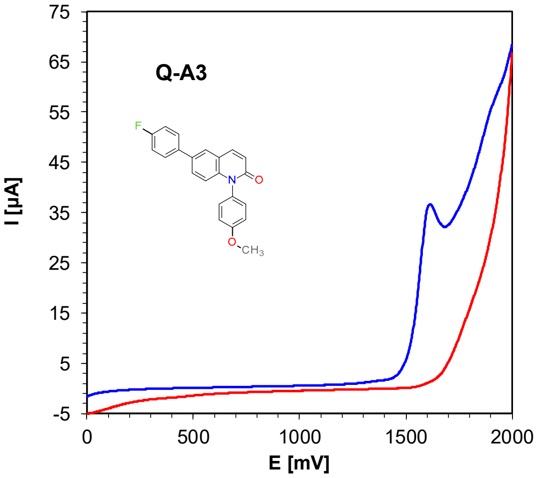

Figure S84. Cyclic voltammogram curves of the Q-A3 oxidation in acetonitrile.

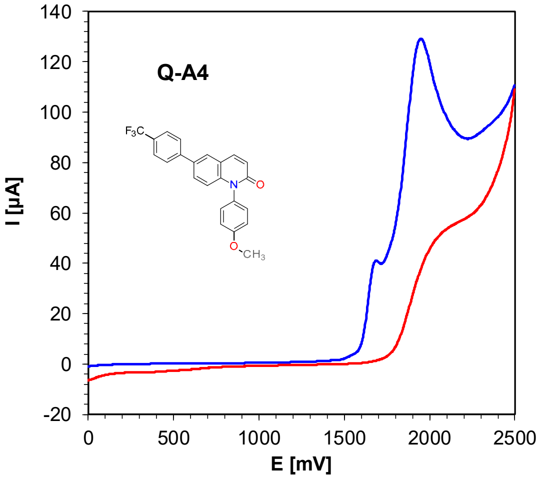

Figure S85. Cyclic voltammogram curves of the Q-A4 oxidation in acetonitrile.

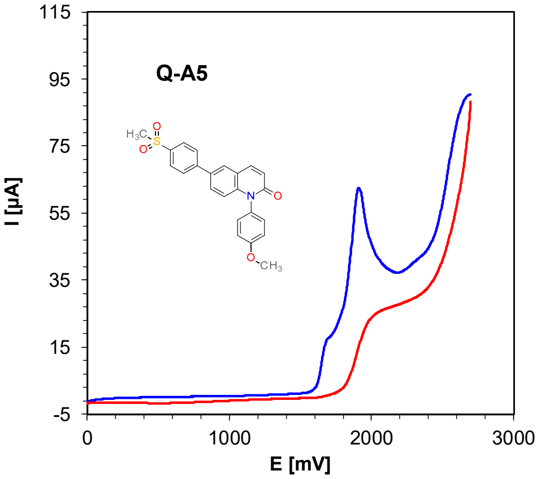

Figure S86. Cyclic voltammogram curves of the Q-A5 oxidation in acetonitrile.

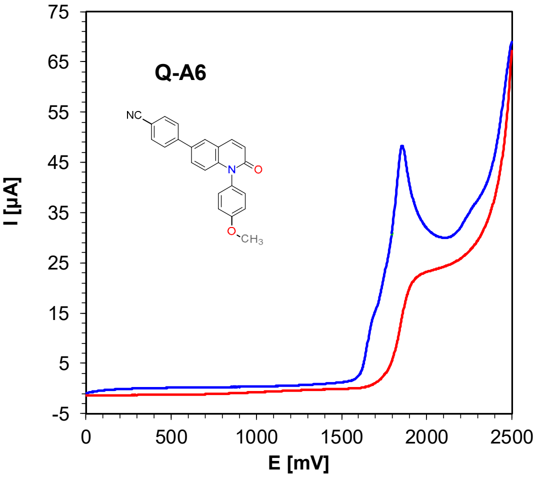

Figure S87. Cyclic voltammogram curves of the Q-A6 oxidation in acetonitrile.

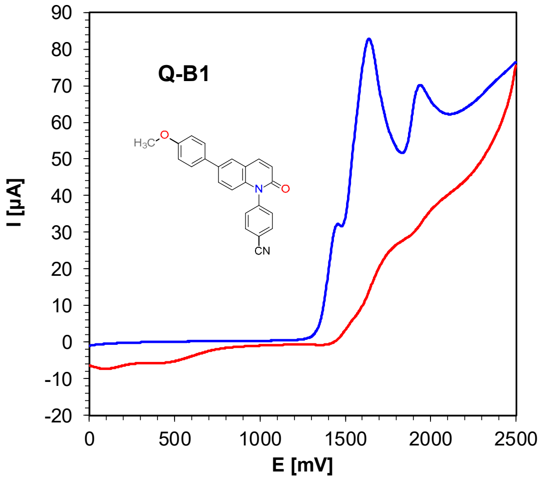

Figure S88. Cyclic voltammogram curves of the Q-B1 oxidation in acetonitrile.

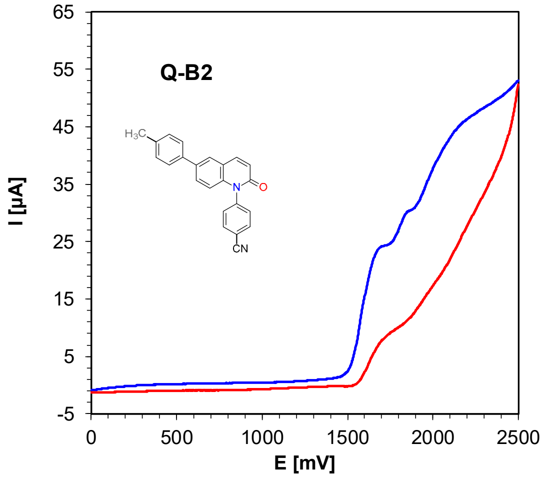

Figure S89. Cyclic voltammogram curves of the Q-B2 oxidation in acetonitrile.

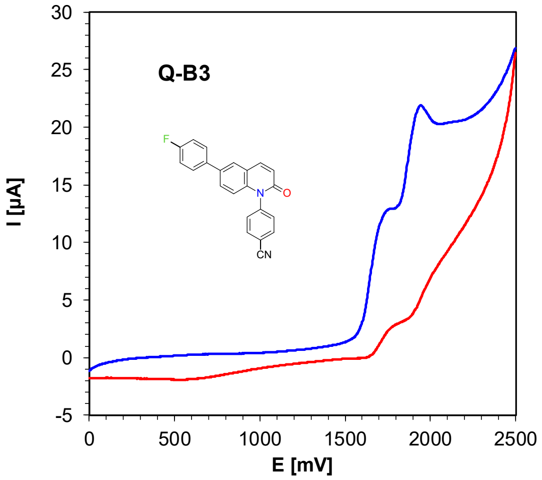

Figure S90. Cyclic voltammogram curves of the Q-B3 oxidation in acetonitrile.

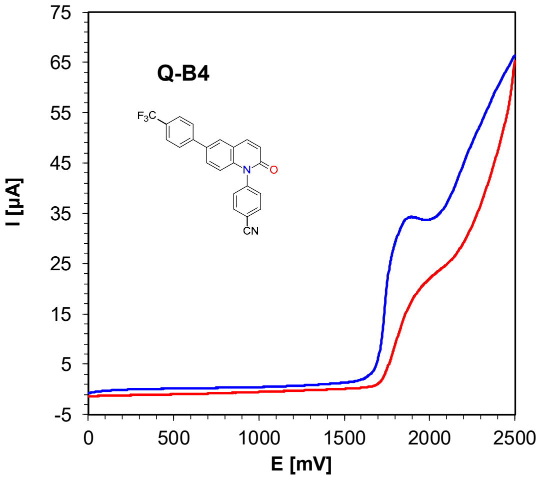

Figure S91. Cyclic voltammogram curves of the Q-B4 oxidation in acetonitrile.

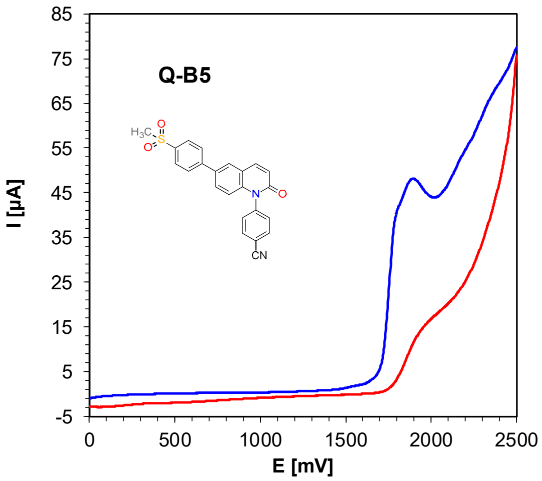

Figure S92. Cyclic voltammogram curves of the Q-B5 oxidation in acetonitrile.

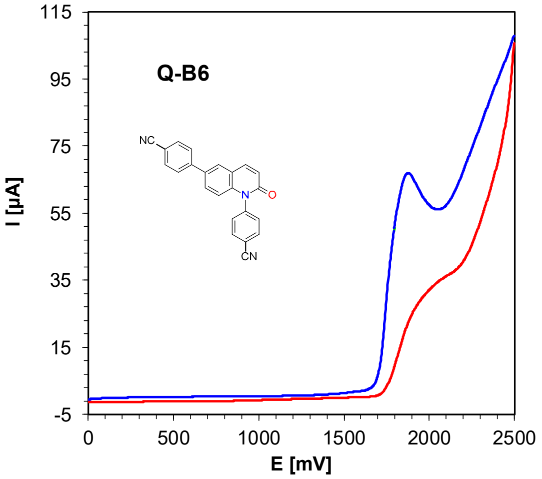

Figure S93. Cyclic voltammogram curves of the Q-B6 oxidation in acetonitrile.

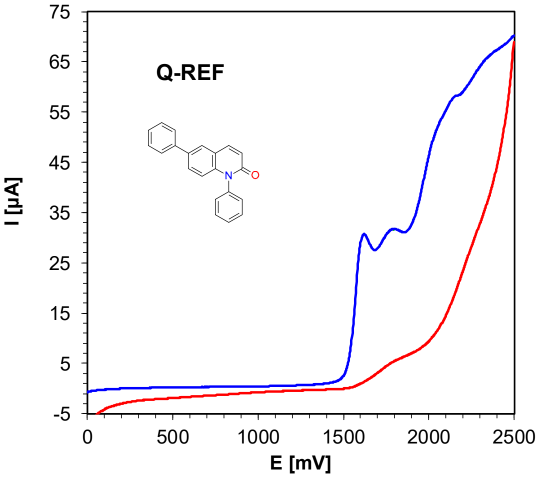

Figure S94. Cyclic voltammogram curves of the Q-REF oxidation in acetonitrile.

5. The Optimized Structures and HOMO and LUMO Orbitals of Investigated 1,6-Diphenylquinolin-2-one Derivatives Free Molecules Determined with the Use of uB3LYP/6-31G* Level of Theory

Compound	HOMO	LUMO
**Q-REF** 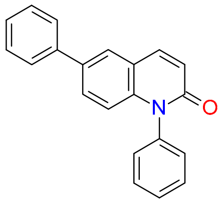 *1,6-difenylochinolin-2-one*	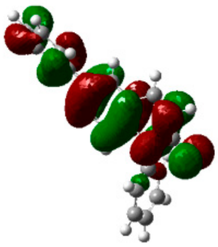	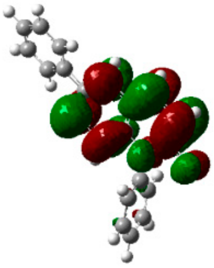
	**SERIE A**	
**Q-A1** 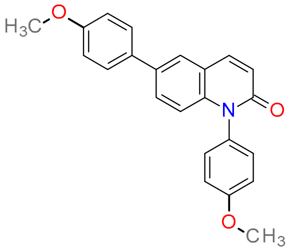 *1,6-bis(4-methoxyphenyl)-quinolin-2-one*	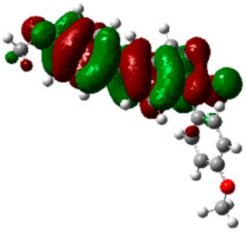	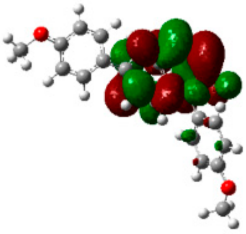

**Q-A2**<br>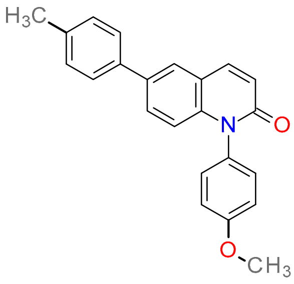<br><br>*1-(4-methoxyphenyl)-6-(4-methylphenyl)quinolin-2-one*	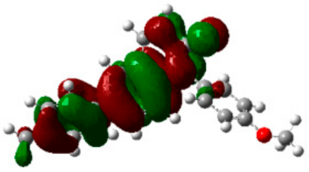	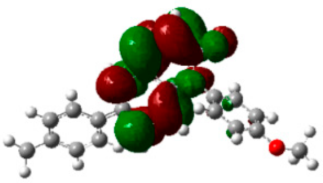
**Q-A3**<br>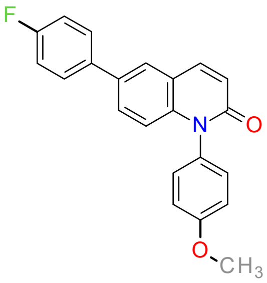<br><br>*1-(4-methoxyphenyl)-6-(4-fluorophenyl)quinolin-2-one*	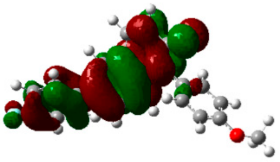	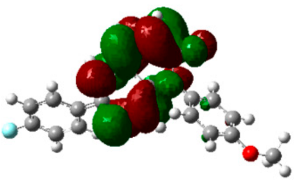
**Q-A4**<br>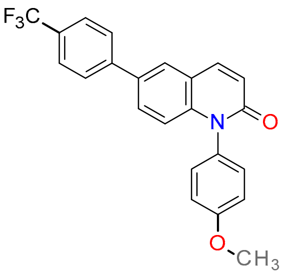<br><br>*1-(4-methoxyphenyl)-6-[4-(trifluoromethyl)phenyl)]-quinolin-2-one*	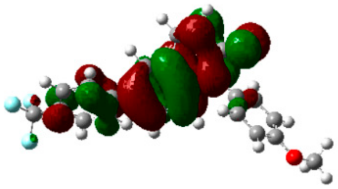	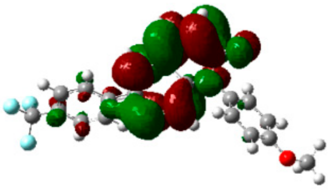
**Q-A5**<br>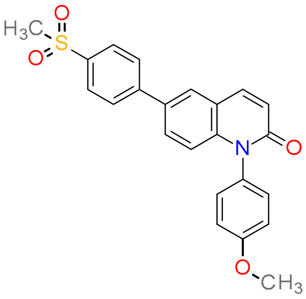<br><br>*1-(4-methoxyphenyl)-6-(4-metylsulfonylfenylo)quinolin-2-one*	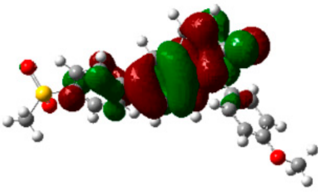	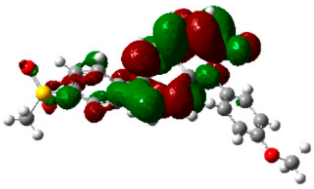

**Q-A6**<br>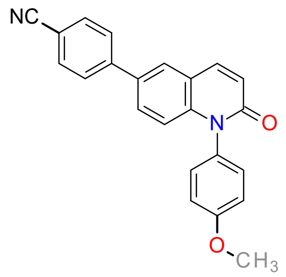<br>*1-(4-methoxyphenyl)-6-(4-nitrylofenylo)quinolin-2-one*	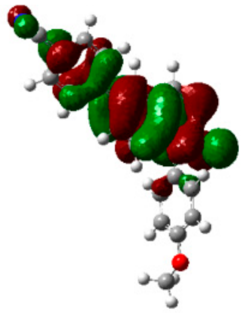	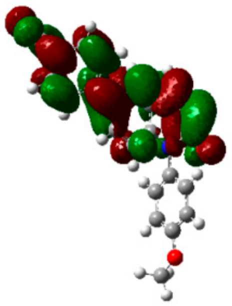
**SERIE B**		
**Q-B1**<br>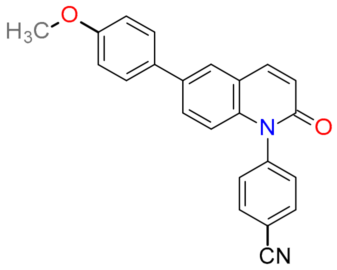<br>*1-(4-cyanophenyl)-6-(4-methoxyphenyl)quinolin-2-one*	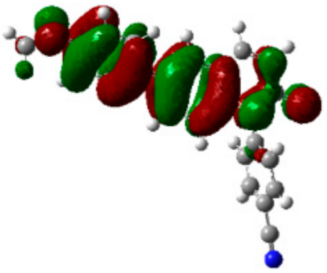	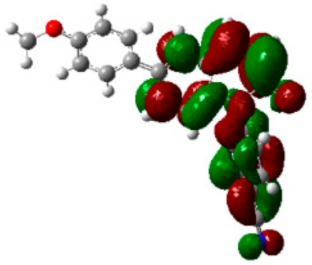
**Q-B2**<br>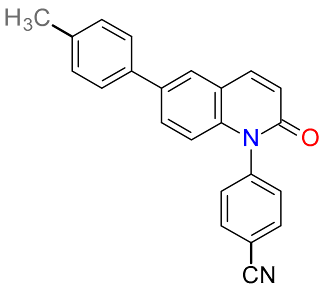<br>*1-(4-cyanophenyl)-6-(4-methylphenyl)quinolin-2-one*	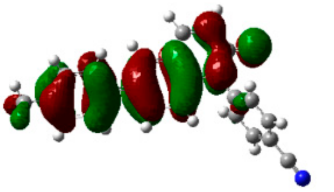	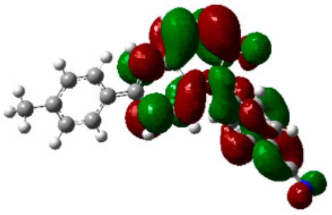
**Q-B3**<br>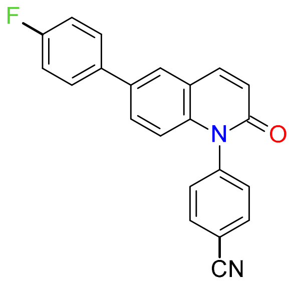<br>*1-(4-cyanophenyl)-6-(4-fluorophenyl)quinolin-2-one*	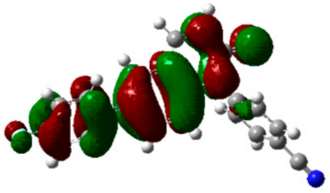	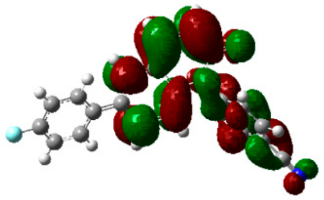

**Q-B4** 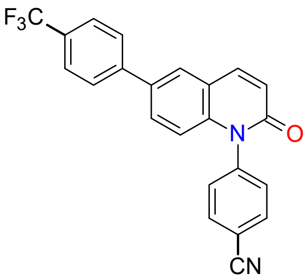 *1-(4-cyanophenyl)-6-(4-trifluoromethyl-phenyl)quinolin-2-one*	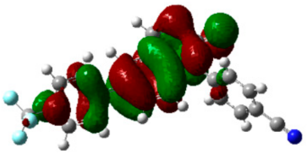	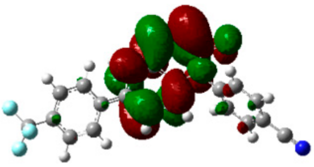
**Q-B5** 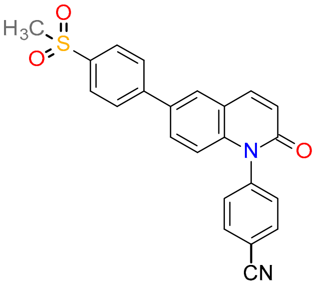 *1- (4-cyanophenyl) -6- (4-methylsulfonyl-phenyl) -quinolin-2-one*	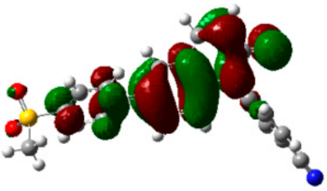	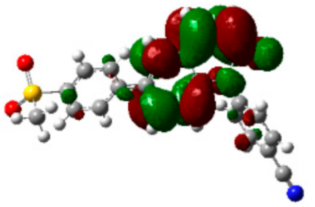
**Q-B6** 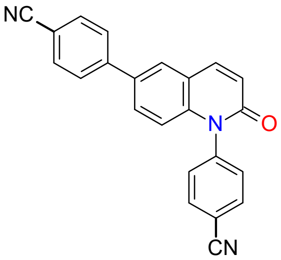 *1,6-bis (4-cyanophenyl) quinolin-2-one*	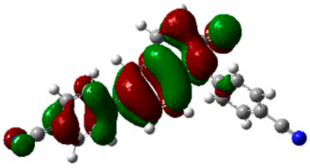	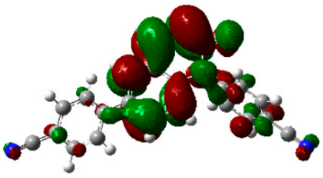

6. Applicability of the 1,6-Diphenylquinolin-2-one for On-line Monitoring Progress of Cationic Photopolymerization of Vinyl Monomer

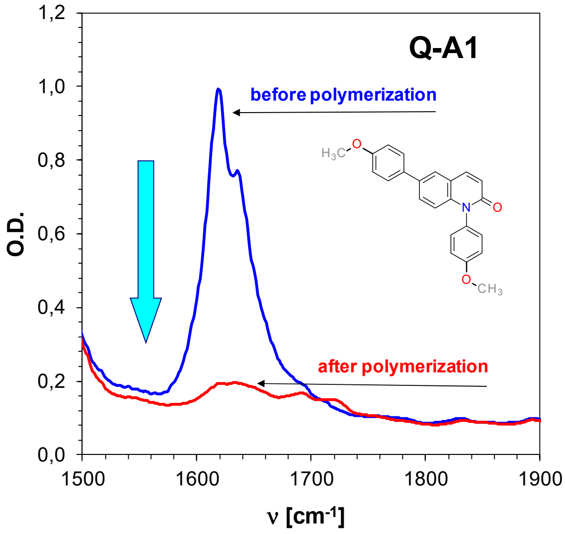

Figure S95. FT-IR spectra before and after photopolymerization of TEGDVE monomer under UV-LED 365 nm irradiation in composition with Q-A1 compound as the photosensitizer.

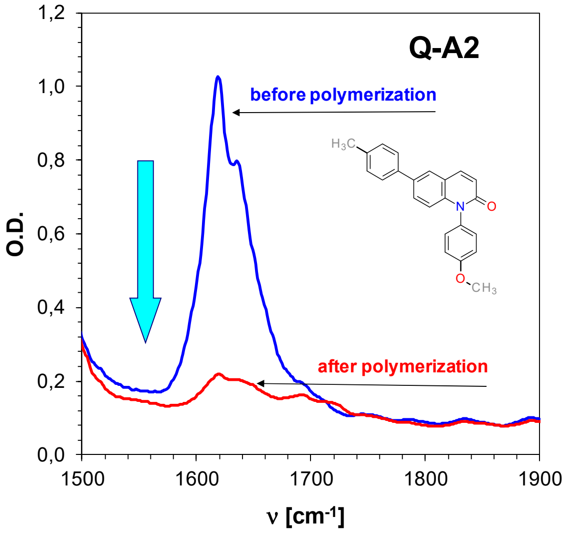

Figure S96. FT-IR spectra before and after photopolymerization of TEGDVE monomer under UV-LED 365 nm irradiation in composition with Q-A2 compound as the photosensitizer.

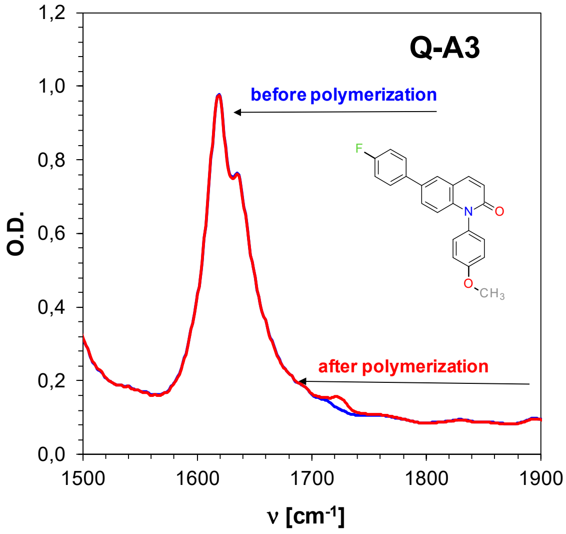

Figure S97. FT-IR spectra before and after photopolymerization of TEGDVE monomer under UV-LED 365 nm irradiation in composition with Q-A3 compound as the photosensitizer.

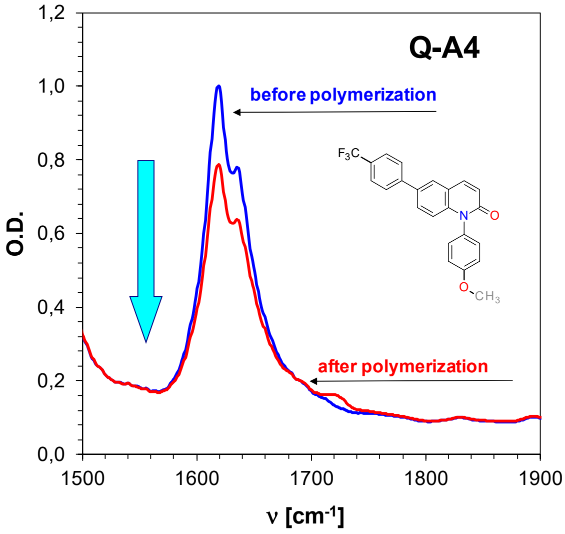

Figure S98. FT-IR spectra before and after photopolymerization of TEGDVE monomer under UV-LED 365 nm irradiation in composition with Q-A4 compound as the photosensitizer.

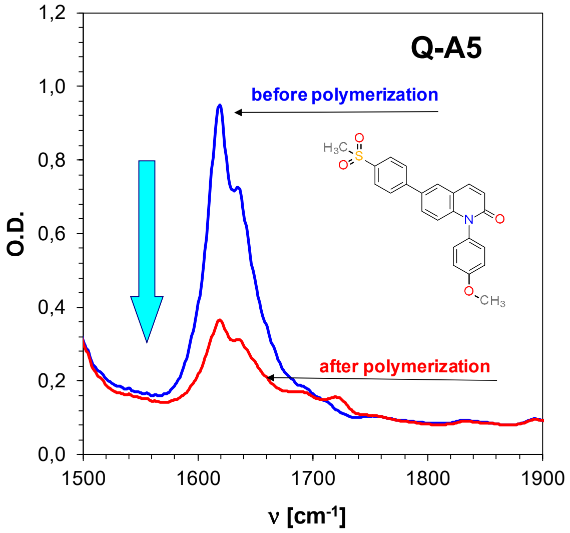

Figure S99. FT-IR spectra before and after photopolymerization of TEGDVE monomer under UV-LED 365 nm irradiation in composition with Q-A5 compound as the photosensitizer.

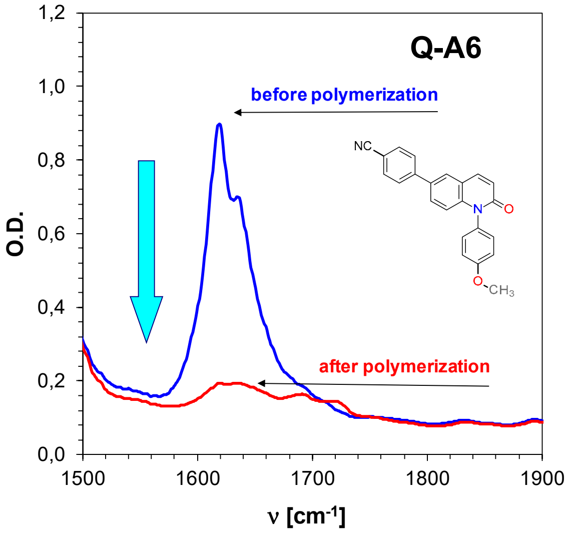

Figure S100. FT-IR spectra before and after photopolymerization of TEGDVE monomer under UV-LED 365 nm irradiation in composition with Q-A6 compound as the photosensitizer.

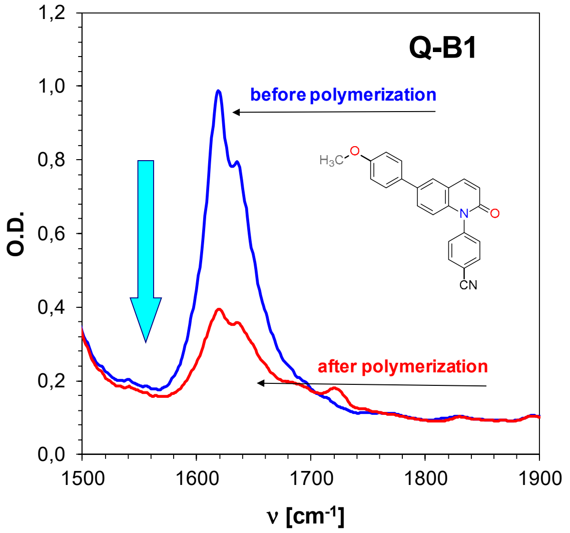

Figure S101. FT-IR spectra before and after photopolymerization of TEGDVE monomer under UV-LED 365 nm irradiation in composition with Q-B1 compound as the photosensitizer.

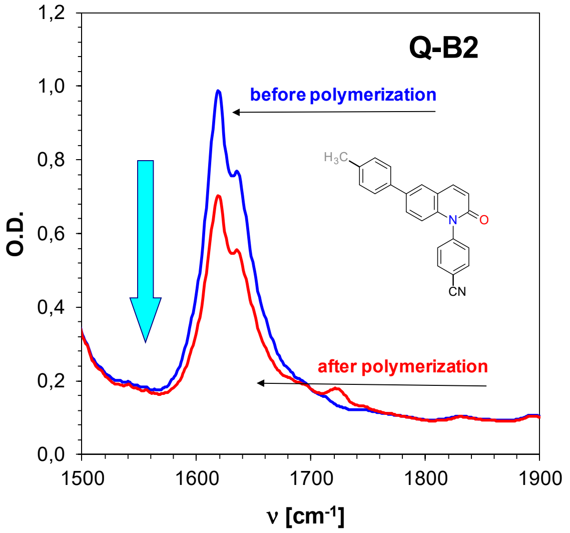

Figure S102. FT-IR spectra before and after photopolymerization of TEGDVE monomer under UV-LED 365 nm irradiation in composition with Q-B2 compound as the photosensitizer.

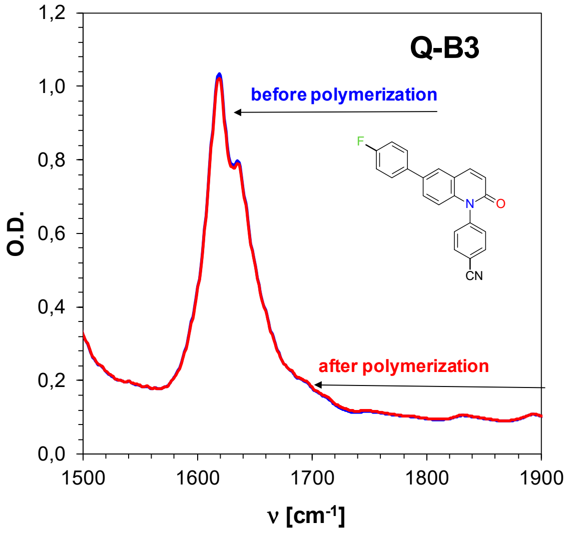

Figure S103. FT-IR spectra before and after photopolymerization of TEGDVE monomer under UV-LED 365 nm irradiation in composition with Q-B3 compound as the photosensitizer.

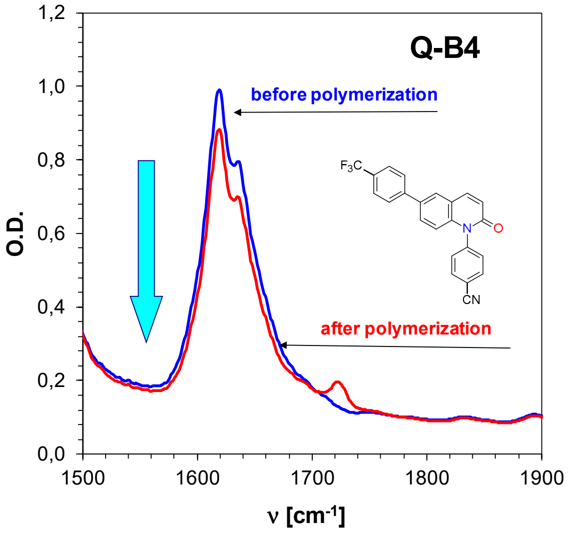

Figure S104. FT-IR spectra before and after photopolymerization of TEGDVE monomer under UV-LED 365 nm irradiation in composition with Q-B4 compound as the photosensitizer.

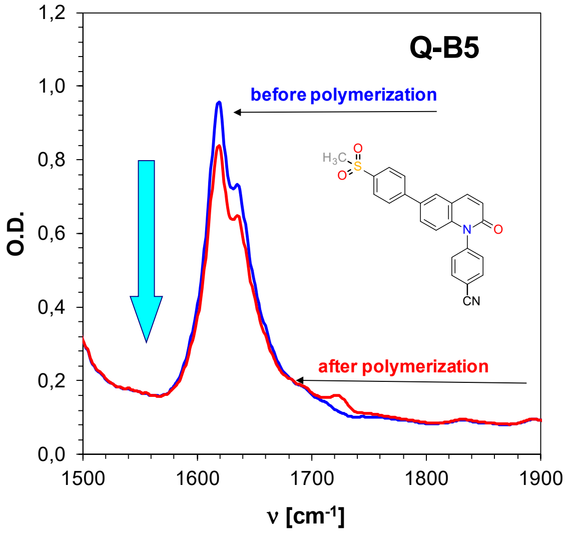

Figure S105. FT-IR spectra before and after photopolymerization of TEGDVE monomer under UV-LED 365 nm irradiation in composition with Q-B5 compound as the photosensitizer.

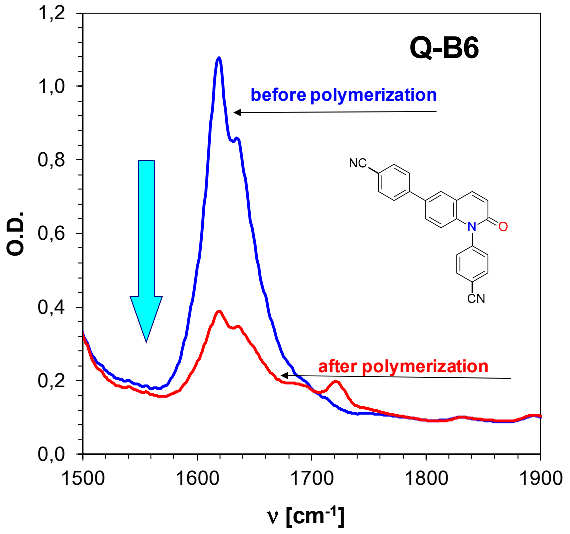

Figure S106. FT-IR spectra before and after photopolymerization of TEGDVE monomer under UV-LED 365 nm irradiation in composition with Q-B6 compound as the photosensitizer.

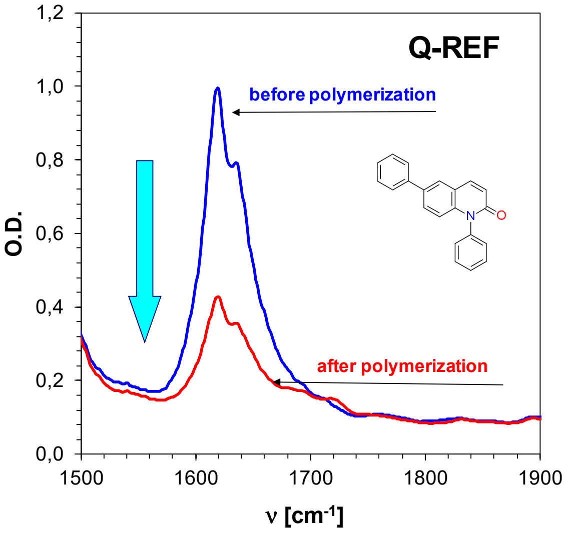

Figure S107. FT-IR spectra before and after photopolymerization of TEGDVE monomer under UV-LED 365 nm irradiation in composition with Q-REF compound as the photosensitizer.
